# Supplementary material for: Programmable DNA shell scaffolds for directional membrane budding
Source: Nat Commun. 2025 Oct 9;16:8972. doi: 10.1038/s41467-025-64298-x (PMC12511405; doi:10.1038/s41467-025-64298-x)
Supplement: Supplementary file 1 — Supplementary Information [file 41467_2025_64298_MOESM1_ESM.pdf]

Supplementary Information for

Programmable DNA shell scaffolds for directional membrane budding

Michael T. Pinner, Hendrik Dietz

Corresponding author: dietz@tum.de

Content:

Supplementary Figures 1 to 25
Supplementary Tables 1 to 26
Supplementary References

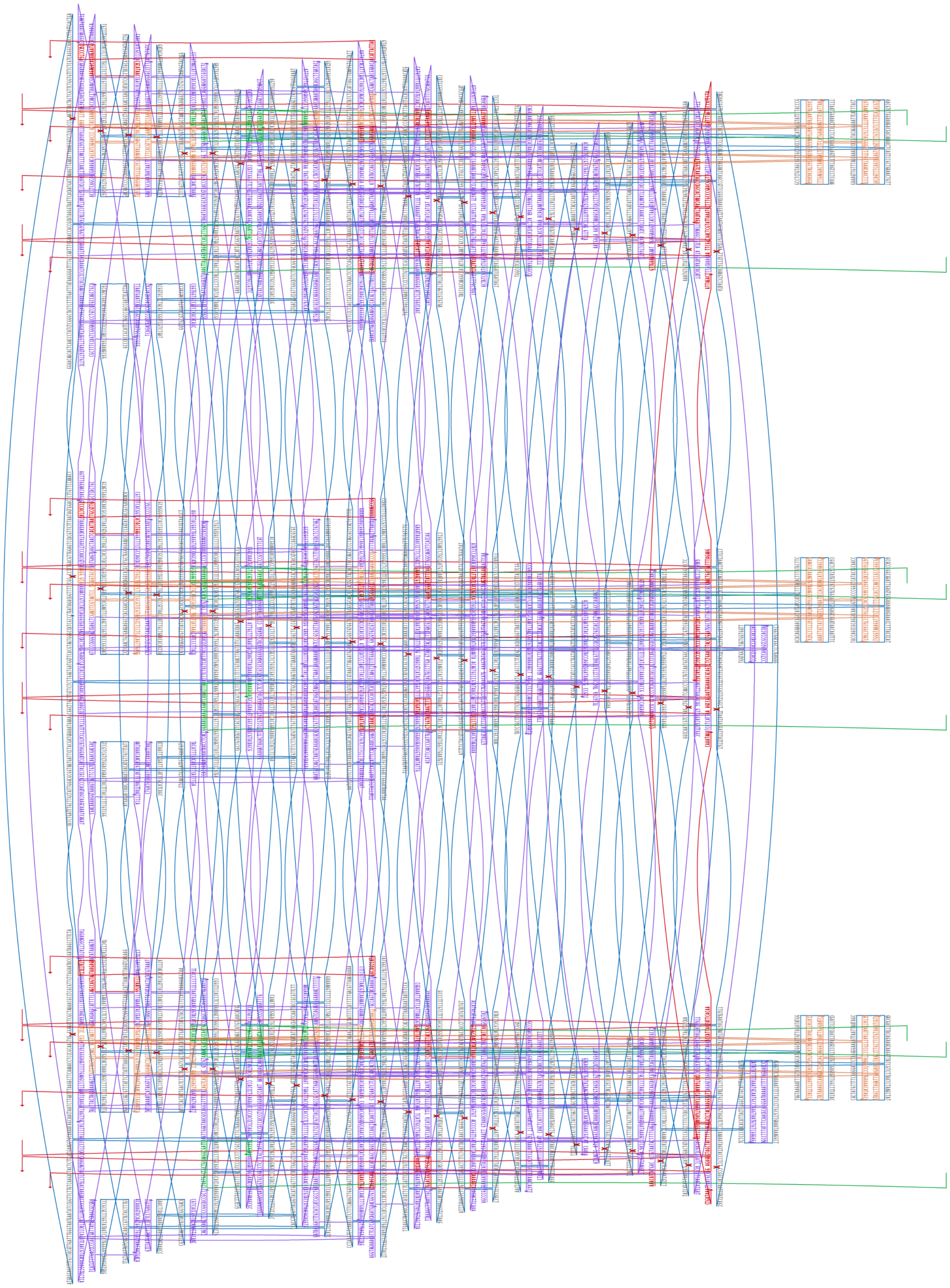

Supplementary Figure 1 | Triangle design map. Exemplary linker handles extending from the origami surface are shown in red (shell-outer face) and green (shell-inner face). The three main domains represent the three sides comprising the triangle. Protrusions used for triangle assembly are highlighted in orange, the shape-complementary recesses are visible as holes in the design.

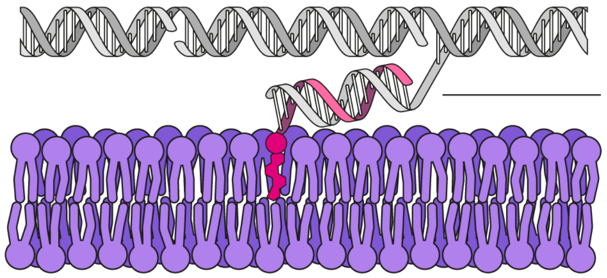

Supplementary Figure 2 | Attachment of origami triangles to vesicles via cholesterol-bearing oligonucleotides. Linker handles extending from the origami surface are hybridised to cholesterol-bearing oligonucleotides with complementary sequences. Cholesterol acts as a lipid membrane anchor to tether the triangles to lipid vesicles.

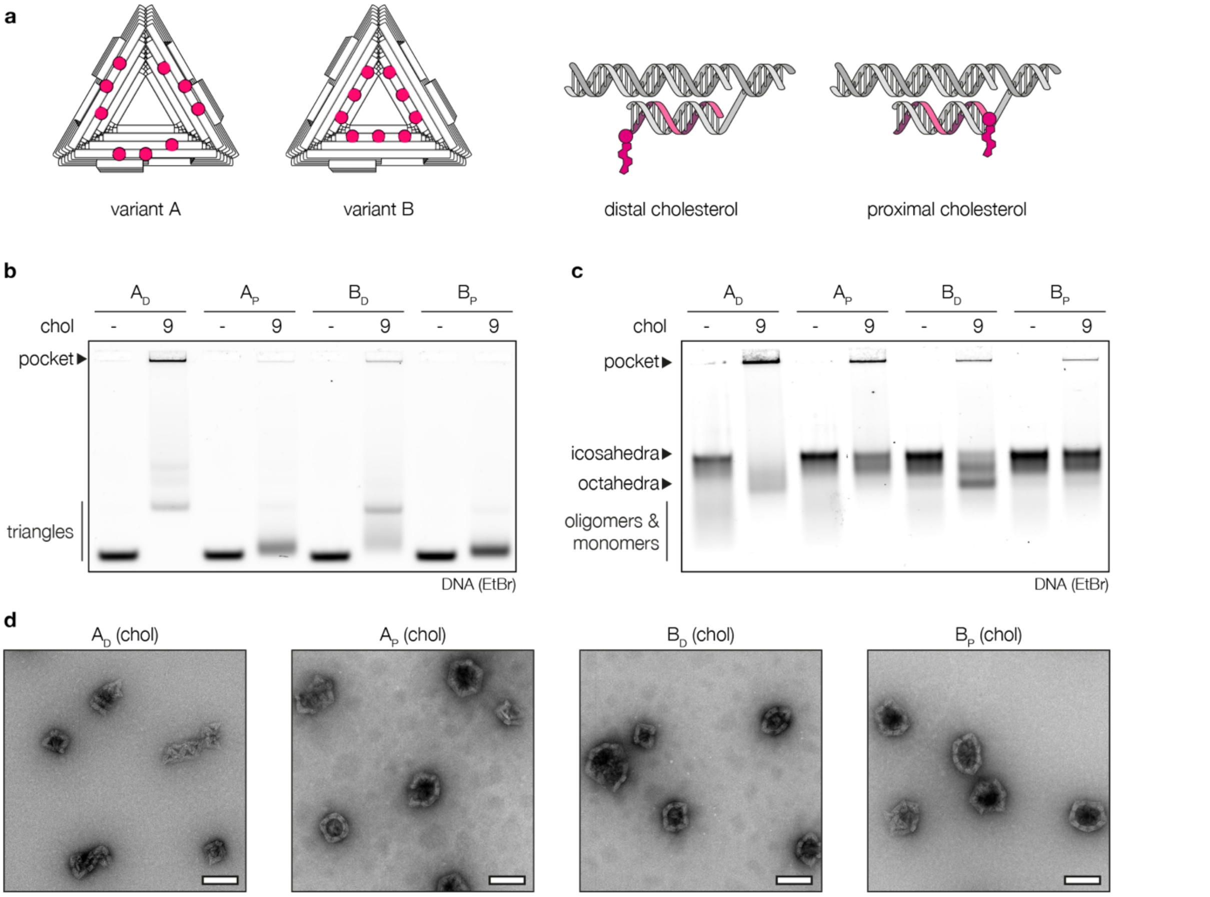

Supplementary Figure 3 | The influence of cholesterol position and orientation on origami triangles. a, Linker handle positions on two different triangle variants, and scheme of distal and proximal cholesterol configurations. The upper, white helix represents the triangle surface (side view), the pink strand represents the cholesterol-bearing oligo (chol-oligo). Cholesterol may be oriented facing away (distal) or towards (proximal) the origami surface. **b,** The overall reach of chol-oligos determines the migration behaviour of origami triangles. When cholesterols are positioned closer to the outer edge and the stacking contacts in a distal configuration (A_D), bands in an agarose gel appear shifted and smeared compared to the same triangles hybridised to unmodified (chol-free) oligos. This effect could be reduced by changing the orientation of the cholesterol to a proximal configuration with respect to the origami, such that the reach of the cholesterol is minimised (A_P). Chol-mediated interactions could be reduced further by positioning the linker handles closer to the centre of the structure, and thus further away from the stacking contacts (B_D & B_P). However, chol configuration appears to have a stronger effect than handle placement, and the order of triangle variants from most to least chol-mediated interactions is thus $A_D > B_D > A_P > B_P$. **c,** The reach of cholesterol influences the assembly of cholesterol-decorated triangles. Triangles hybridised to unmodified oligos assemble mostly into icosahedral species, but when chol-oligos are introduced, distal cholesterol (A_D, B_D) promotes the formation of primarily octahedral species and aggregates. Proximal cholesterol (A_P, B_P) does not noticeably influence the assembly behaviour, with most triangles forming icosahedra. As in b, the least chol-mediated interactions were observed in sample B_P, showing that reducing the reach of individual cholesterols by positioning them closer to the centre of the triangles and choosing proximal configurations reduces unwanted interactions. **d,** Representative TEM micrographs of the assemblies shown in c (chol-bearing triangles). Scale bars: 100 nm. Source data are provided as a Source Data file.

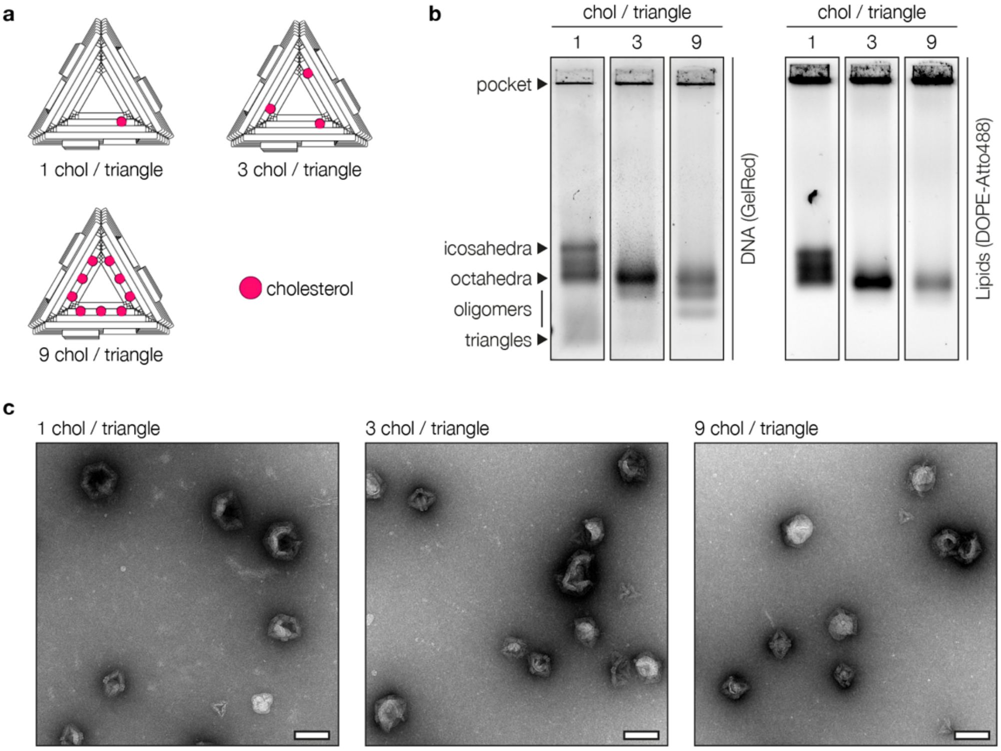

Supplementary Figure 4 | The influence of cholesterol count per triangle in assembly reactions containing GVs. a, Cholesterol positions on the bottom face of the triangles used in this experiment. **b,** Agarose gel of assembly reactions including triangles carrying either 1, 3 or 9 cholesterols and fluorescently-labelled GVs. Left: Gel scanned for GelRed, visualising DNA. Right: Gel scanned for Atto488, visualising DOPE-Atto488 labelled membranes. Whilst optimising cholesterol positioning on the triangle can reduce unexpected assembly behaviour, introducing lipid vesicles yet again shifts the assembly towards octahedra, the degree of which increases with the number of cholesterols per triangle. The colocalization of lipid and DNA bands suggests the formation of lipid-DNA hybrid structures. **c,** TEM images confirm the formation of lipid-DNA hybrid structures in all samples. However, whilst lipid vesicles commonly fill up the inner cavity of the DNA shells in the 3 & 9 chol / triangle samples, this was not always the case in the 1 chol / triangle sample. Scale bars: 100 nm. Source data are provided as a Source Data file.

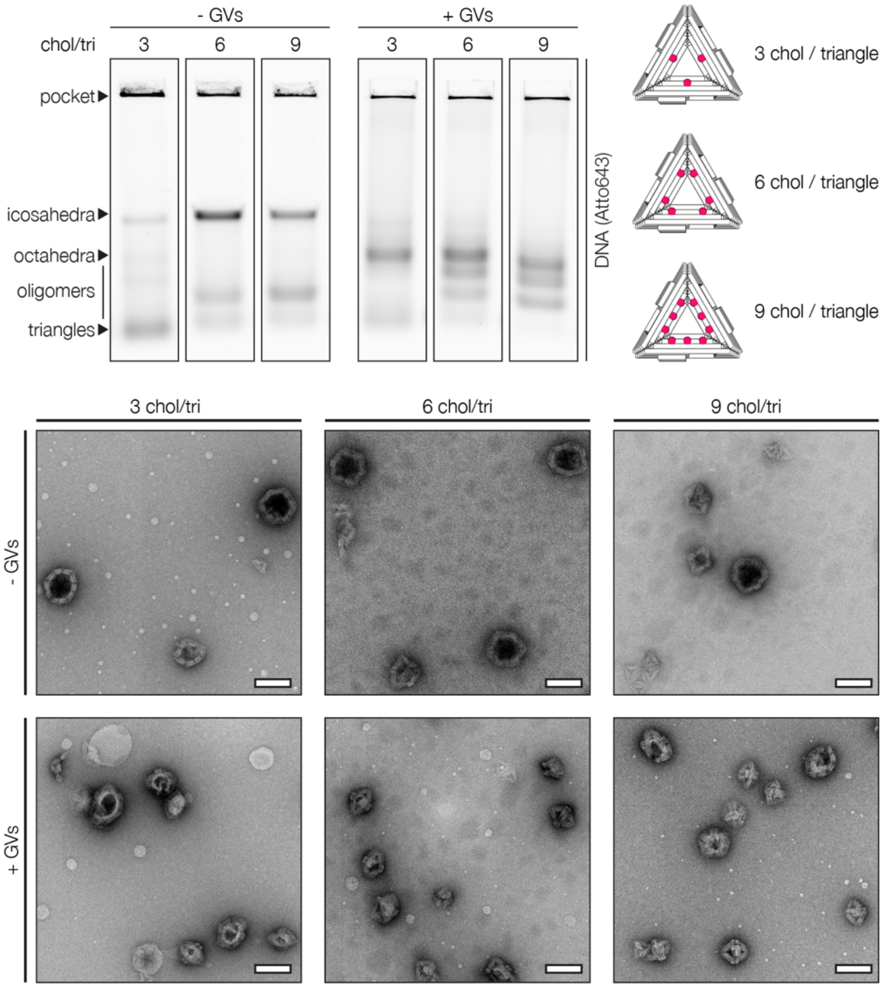

Supplementary Figure 5 | GVs shift the assembly of cholesterol-bearing triangles towards octahedral shells. The assembly behaviour of cholesterol-bearing triangles, tagged with Atto643 for visualisation, depends on the presence or absence of GVs. Without GVs, triangles mainly assemble into icosahedral shells as per their design. With GVs, however, most shells detected by agarose gel electrophoresis are octahedral. The abundance of smaller, intermediate species between closed shells and monomeric triangles increases with the number of cholesterols per triangle and mostly represents aggregated triangles. Top: Agarose gel showing shell assembly in absence or presence of GVs (DNA channel, Atto643). Bottom: TEM micrographs of the assembly reactions. Scale bars: 100 nm. Source data are provided as a Source Data file.

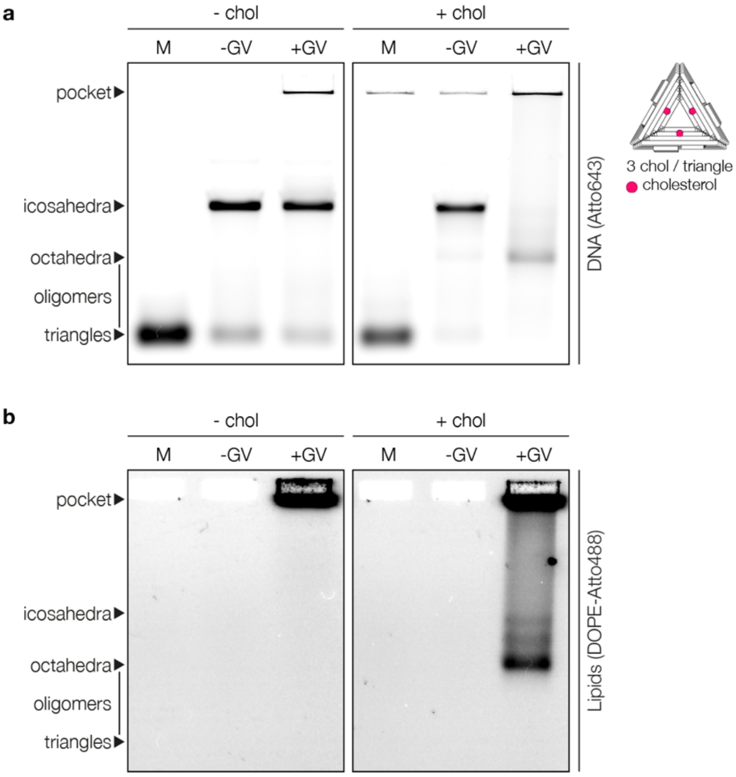

Supplementary Figure 6 | GVs only alter the assembly behaviour of chol-bearing triangles. a, Agarose gel of DNA shells made visible by Atto643-bearing oligonucleotides.Triangles assemble predominantly into icosahedral shells even if either GVs or chol-bearing oligonucleotides are present in the sample. However, if both GVs and chol-bearing oligonucleotides are present in a sample at the same time, the assembly of triangles is shifted towards octahedral species. **b,** The gel in a rescanned for visualisation of DOPE-Atto488 species included in the vesicle membranes. Only the sample containing chol-bearing triangles and GVs produced lipid bands coinciding with the DNA shell bands in a, therefore indicating that the octahedral shells found in a are mostly octahedral DNA-shell-coated vesicles (M: triangle monomers). Source data are provided as a Source Data file.

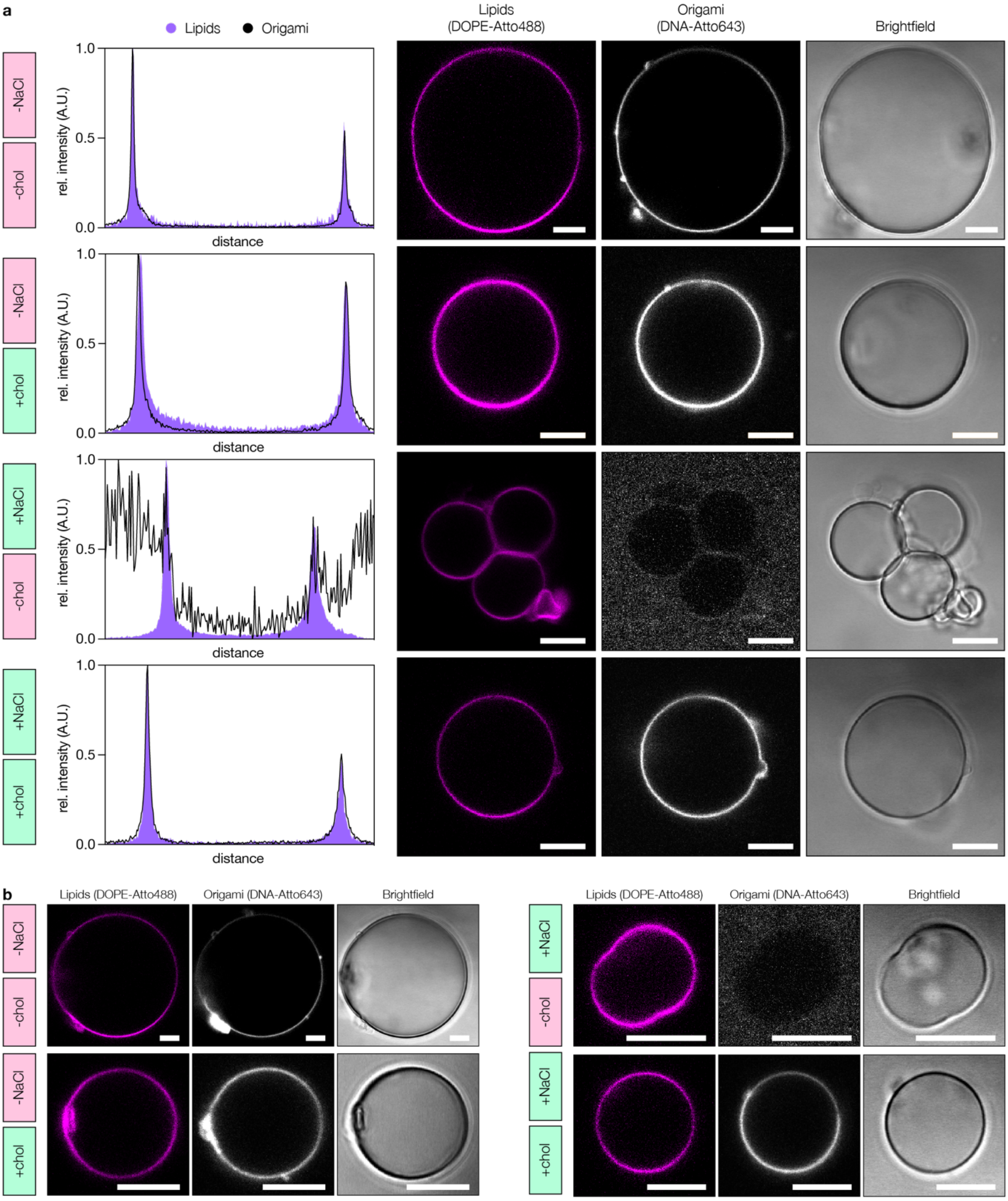

Supplementary Figure 7 | Sodium chloride prevents non-specific adsorption of DNA origami onto giant vesicles. a, Cross-sectional intensity profiles and channel-separated confocal microscopy images of giant vesicles mixed with DNA origami triangles. Conditions: +/- cholesterol-modified oligonucleotides (chol) and +/- 300 mM NaCl, as indicated. At low-salt conditions (5 mM $MgCl_2$, 5 mM NaCl), triangles adsorb onto lipid vesicles even if not hybridised to chol-oligos. By adding additional 300 mM NaCl, non-specific origami-vesicle association is suppressed without interfering with cholesterol-mediated association. **b,** Additional views. Scale bars: 10 µm

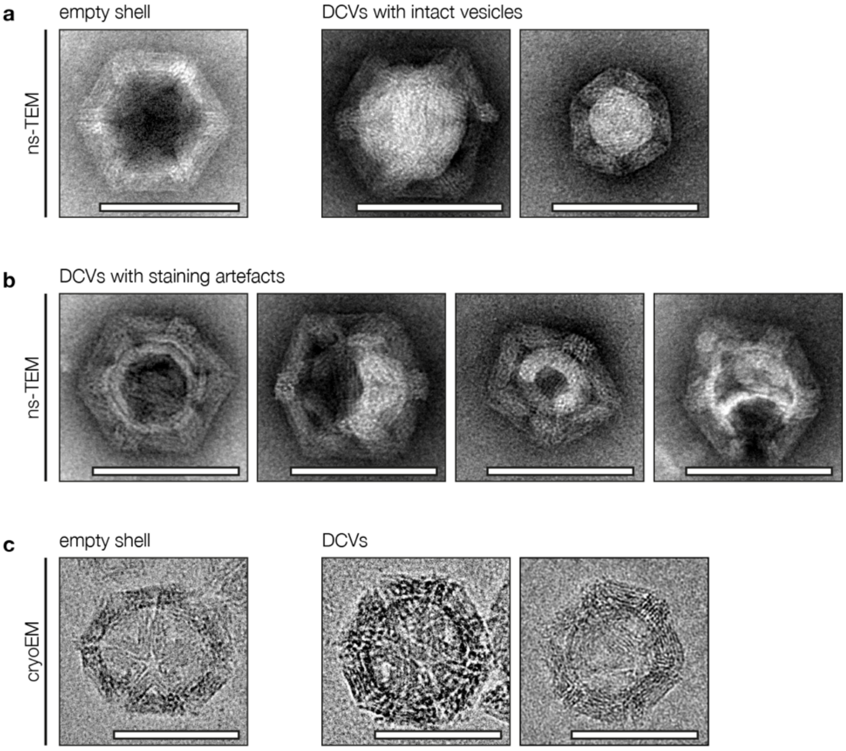

Supplementary Figure 8 | Appearance of DCVs in TEM micrographs. a, Uranium stain for negative stain TEM (ns-TEM) increases the contrast of biological samples, allowing for easy differentiation between different shell species. Empty shells accumulate stain within their cavity, rendering it black, while lipid vesicles within DCVs displaces the stain, rendering the cavity white. **b,** Staining and subsequent drying of sample grids commonly alters the appearance of lipid vesicles. Consequently, the vesicles within DCVs commonly appear distorted, incomplete or broken in ns-TEM. **c,** Cryogenic electron microscopy (cryoEM) allows imaging of DCVs in their native state without sample staining or drying steps. DCVs can be distinguished from empty shells by a dark ring lining the inner cavity of the DCV, representing the lipid vesicle. The artefacts shown in b are absent in cryoEM micrographs. All scale bars: 100 nm

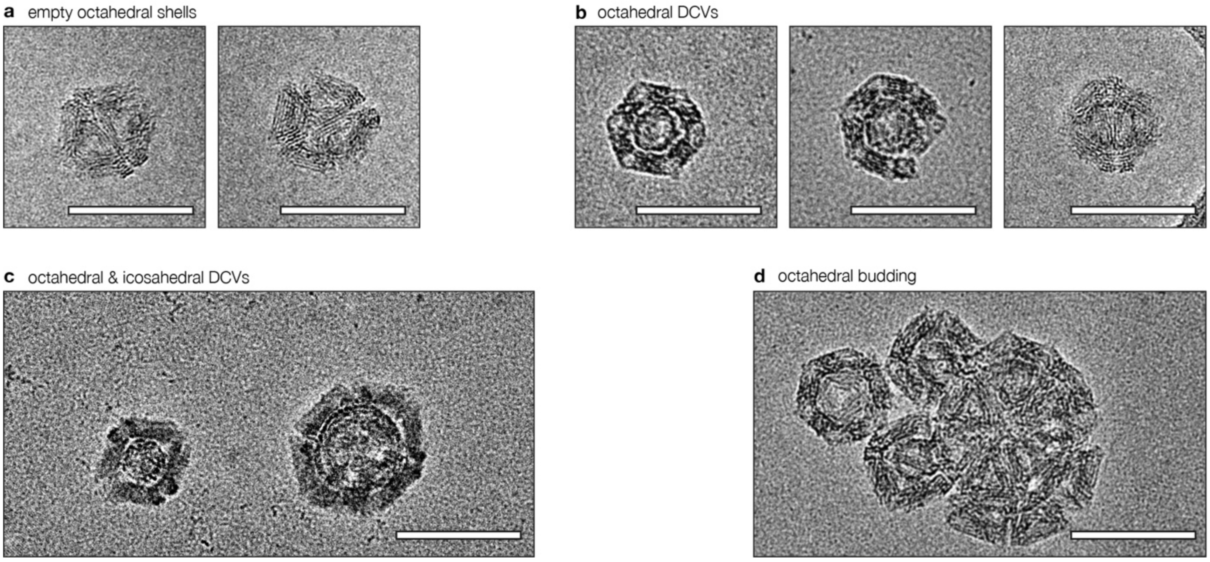

Supplementary Figure 9 | CryoEM images of octahedral DCVs. a, Appearance of empty octahedral shells. **b,** Octahedral DCVs. Differences in appearance are due to different orientations of the particles. **c,** Size comparison between an octahedral (left) and icosahedral (right) DCV. As icosahedral particles are composed of 20 triangles (versus 8 in octahedra), the inner cavity and the overall size of the particle are both larger than in octahedral species. **d,** Budding of two octahedral DCVs. To the left of the two triangle-covered buds is an empty octahedral shell. All scale bars: 100 nm.

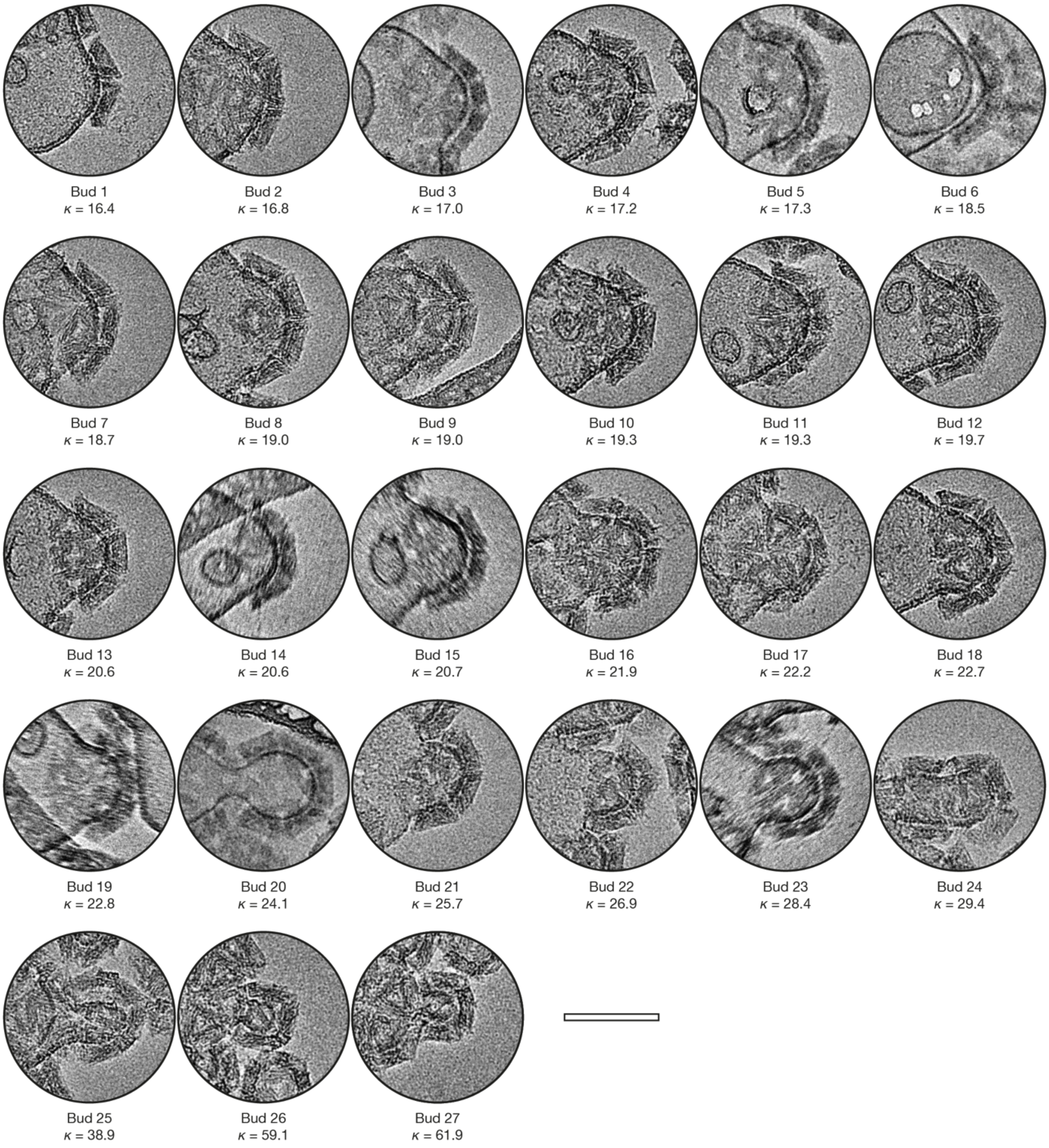

Supplementary Figure 10 | Curvature analysis of growing buds. CryoEM micrographs of outward-directed buds on LVs, sorted by increasing curvature from top to bottom. Larger triangle assemblies adopting icosahedral geometry (e.g., bud 20) induce shallower curvature near completion than similarly mature octahedral assemblies (e.g., buds 26 & 27). In contrast, early-stage assemblies, such as the triangle dimer shown in bud 1, induce only a small bump in the vesicle membrane, illustrating the coupling between curvature maturation and shell completion. Most buds shown lie in between both extremes and differ only slightly in their curvature values. Curvatures (κ, in μm^{-1}) were measured by curve fitting using Kappa (Fiji) and correspond to the inverse radius of the osculating circle approximating the membrane curvature underneath the triangle coat. As buds may be viewed at an angle, measured values may underestimate true curvature. Scale bar: 100 nm (all images).

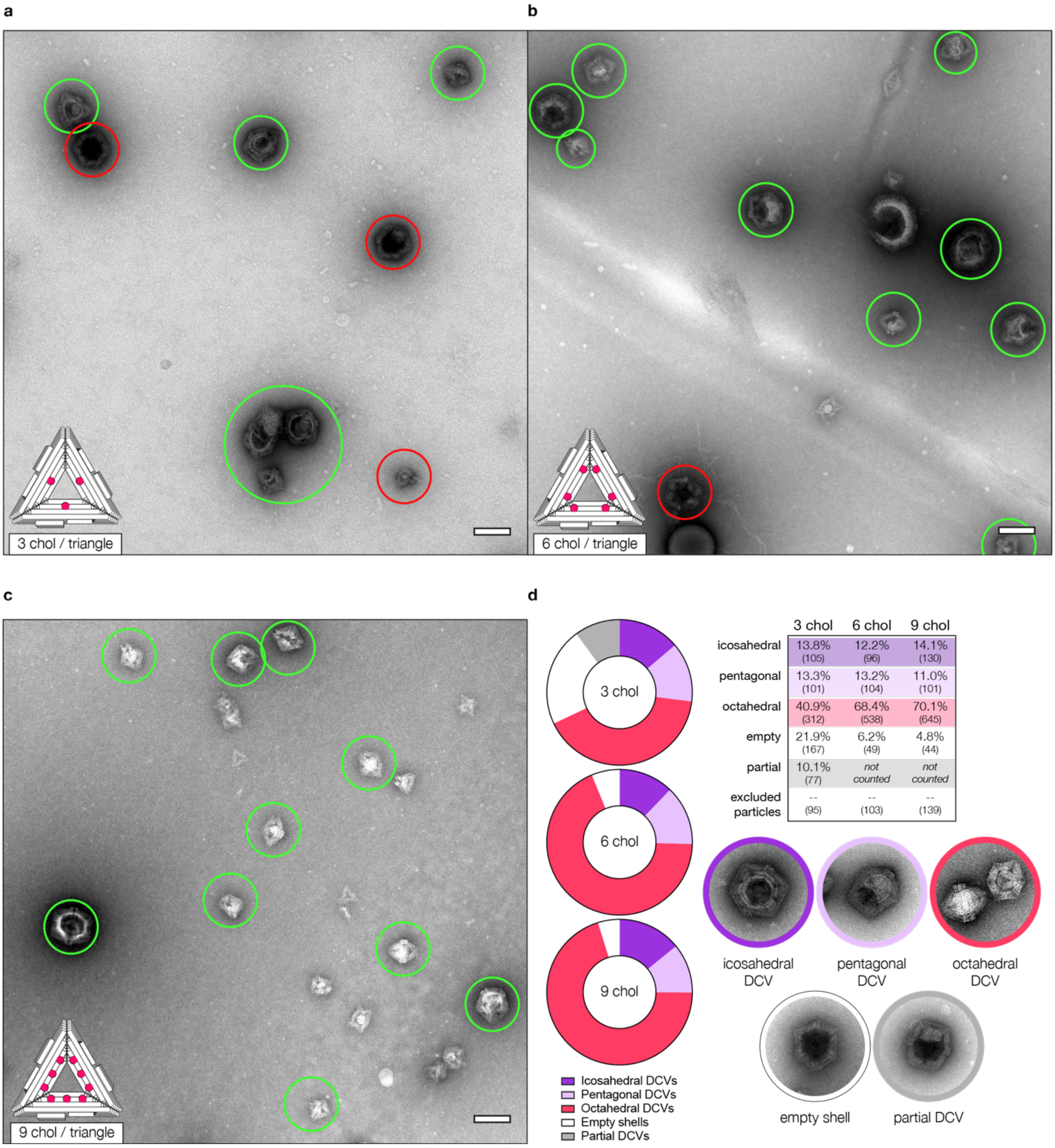

	3 chol	6 chol	9 chol
icosahedral	13.8% (105)	12.2% (96)	14.1% (130)
pentagonal	13.3% (101)	13.2% (104)	11.0% (101)
octahedral	40.9% (312)	68.4% (538)	70.1% (645)
empty	21.9% (167)	6.2% (49)	4.8% (44)
partial	10.1% (77)	*not counted*	*not counted*
excluded particles	-- (95)	-- (103)	-- (139)

Supplementary Figure 11 | DCVs obtained from triangles with 3, 6 or 9 cholesterols. Full fields of view from negatively stained TEM micrographs of DCV samples formed using triangles with **a,** 3; **b,** 6; or **c,** 9 cholesterols. Irregular particles (e.g., unusually large or ambiguous shapes) and free monomers or oligomers were not marked. All images were acquired under identical conditions. Scale bars: 100 nm. **d,** Frequency of particle subspecies by cholesterol count. Besides octahedral and icosahedral DCVs, pentagonal DCVs—likely representing scarred icosahedra—were consistently observed. Lower cholesterol content resulted in more empty shells, while higher counts promoted the formation of octahedral DCVs. Triangles with 3 cholesterols also produced a distinct population of incomplete DCVs with small internal vesicles (partial DCVs), less prevalent at higher cholesterol numbers. Although some may be staining artefacts, their enrichment in low-cholesterol samples suggests weaker membrane binding under these conditions. Particles with ambiguous or unclear morphology were excluded from the analysis. Numbers in brackets indicate absolute particle counts (n).

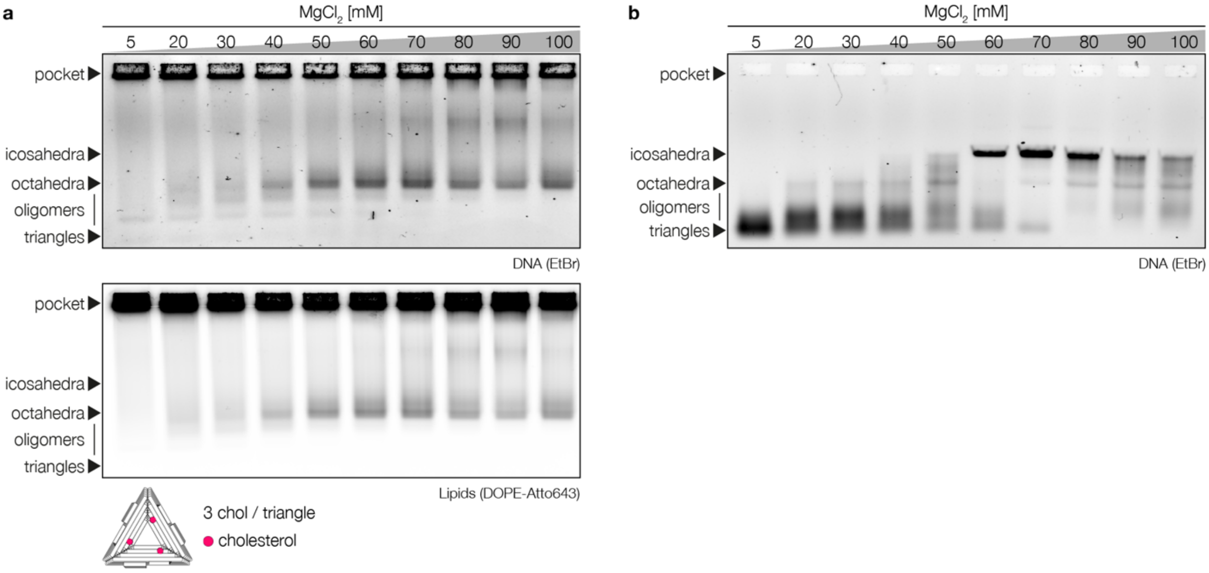

Supplementary Figure 12 | Magnesium screen of membrane-bound and vesicle-free assembly reactions. a, Agarose gel of triangle assembly on GVs. While DCVs may also form at lower magnesium concentrations, assembly is most efficient between 60-70 mM $MgCl_2$ (in 300 mM NaCl). Monomeric triangles remain bound to GVs which are retained in the gel pockets. Faint bands of sandwich dimers—cholesterol-mediated dimers—are visible even at low Mg^{2+} levels. Above the assembly optimum, DCVs form slightly less efficiently. **b,** Agarose gel of vesicle-free triangle assembly. At the magnesium optimum, triangles assemble efficiently into icosahedra, but above and below it, a mix of octahedra and intermediates dominates. Source data are provided as a Source Data file.

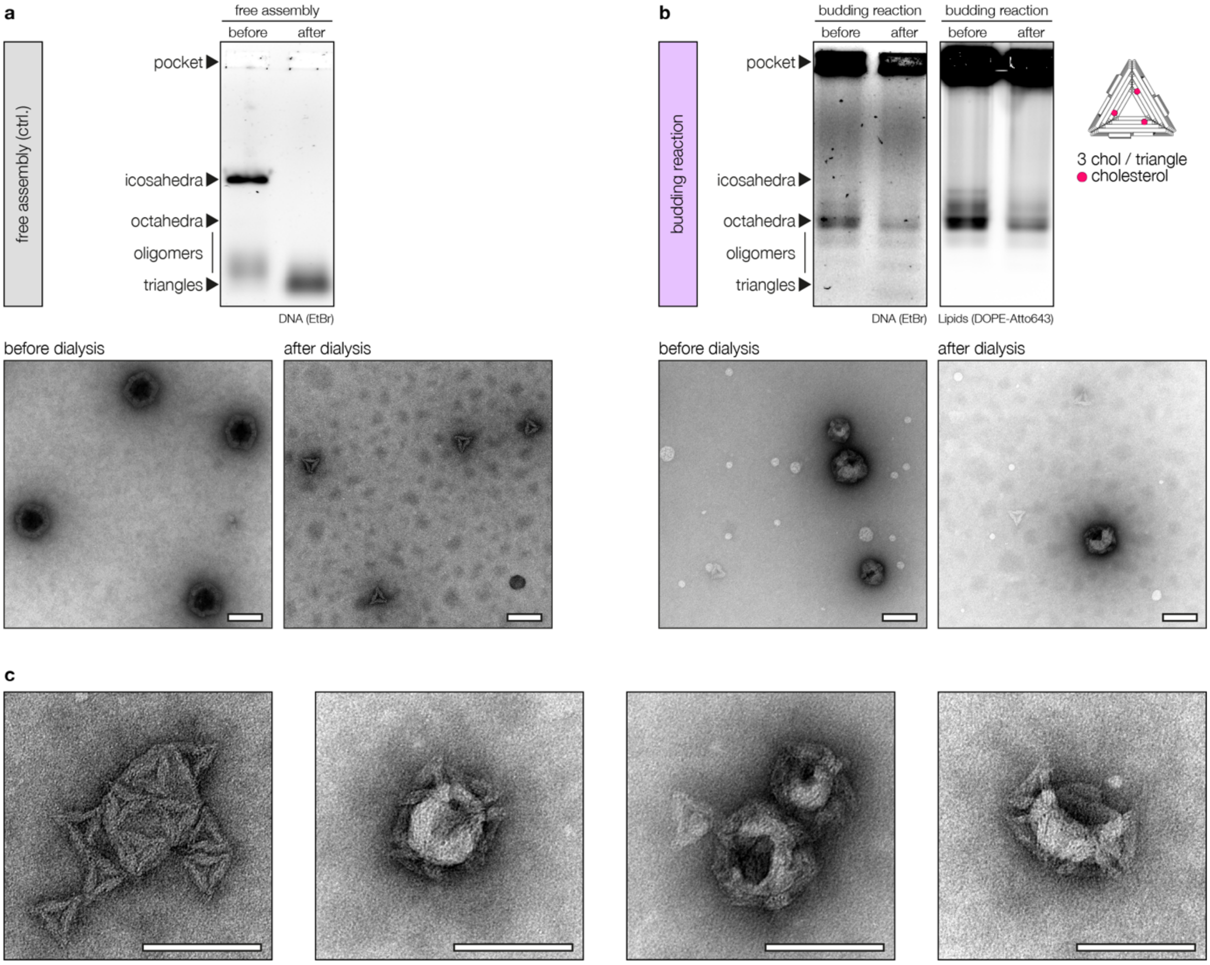

Supplementary Figure 13 | Stability of DCVs under low-salt conditions. To assess the structural stability of DCVs in reduced ionic strength, both free triangle assemblies (without vesicles) and budding reactions were dialysed against sodium buffer, lowering the $MgCl_2$ concentration from 60 mM to 5 mM. **a,** Stability of free shells. Top: Agarose gel electrophoresis (EtBr-stained) showing DNA shells before and after dialysis. Under high-salt conditions, triangles efficiently assembled into icosahedral shells. Upon Mg^{2+} depletion, the shells disassembled into monomeric triangles. Bottom: TEM micrographs corroborate the gel data, showing intact shells before dialysis and monomers afterwards. **b,** Stability of DCVs. Top: Agarose gel showing DCVs before and after dialysis (left: DNA channel (EtBr); right: lipid channel (DOPE-Atto643)). While DCVs persist post-dialysis, a laddering pattern beneath the octahedral DCV band in the DNA channel suggests partial destabilisation. Bottom: TEM images confirm the co-existence of intact DCVs and monomeric triangles post-dialysis. **c,** Close-up TEM micrographs of dialysed DCVs. Free triangles tend to cluster and appear associated with lipid patches. DCVs remain present but appear structurally compromised and less ordered compared to those kept at assembly conditions. All scale bars: 100 nm. Source data are provided as a Source Data file.

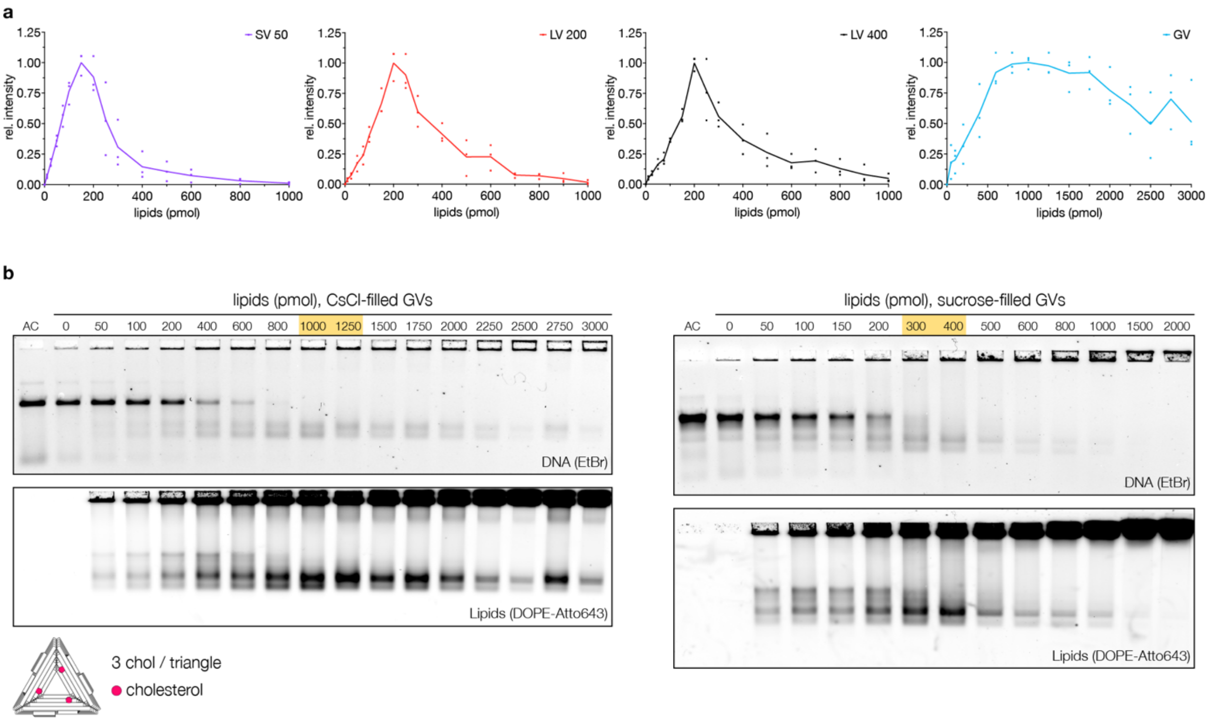

Supplementary Figure 14 | DCV budding as a function of lipid quantity and vesicle size. a, Normalised DCV yields (lipid band intensities of octahedral and icosahedral DCVs, combined) from vesicles of varying sizes, quantified via agarose gel electrophoresis (n = 3; n = 2 for 1000 pmol of SV50 & LV200). Budding reactions were prepared by adding the indicated quantities of lipids in the form of vesicles, ranging from SVs (⌀ 50 nm), LVs (⌀ 200 & 400 nm), to GVs, with a constant amount of triangles (4.5 µl at 15 nM). DCVs are formed most efficiently between 100-200 pmol lipids for all tested vesicle sizes except GVs (note the broader range of the x-axis for GVs). This deviation may result from lamellarity differences, as multilamellar vesicles contribute less accessible membrane area per pmol lipid. Extruding GVs to produce smaller vesicles likely reduced their lamellarity[25]. Data points were obtained from independent experiments using freshly prepared vesicles; the curves indicate their mean. Source data are provided as a Source Data file. **b,** Agarose gels probing DCV yields at varying amounts of lipids. Left: Exemplary gel of budding reactions using caesium buffer-filled GVs used to obtain the curves in a and b. Right: Budding from GVs filled with sucrose buffer, showing a lower lipid optimum. The absence of salts in sucrose buffer may yield fewer multilamellar vesicles, increasing the accessible membrane area per pmol lipid. Yellow-marked lanes indicate optima. Bottom: Cholesterol placement on the triangle surface. Source data are provided as a Source Data file.

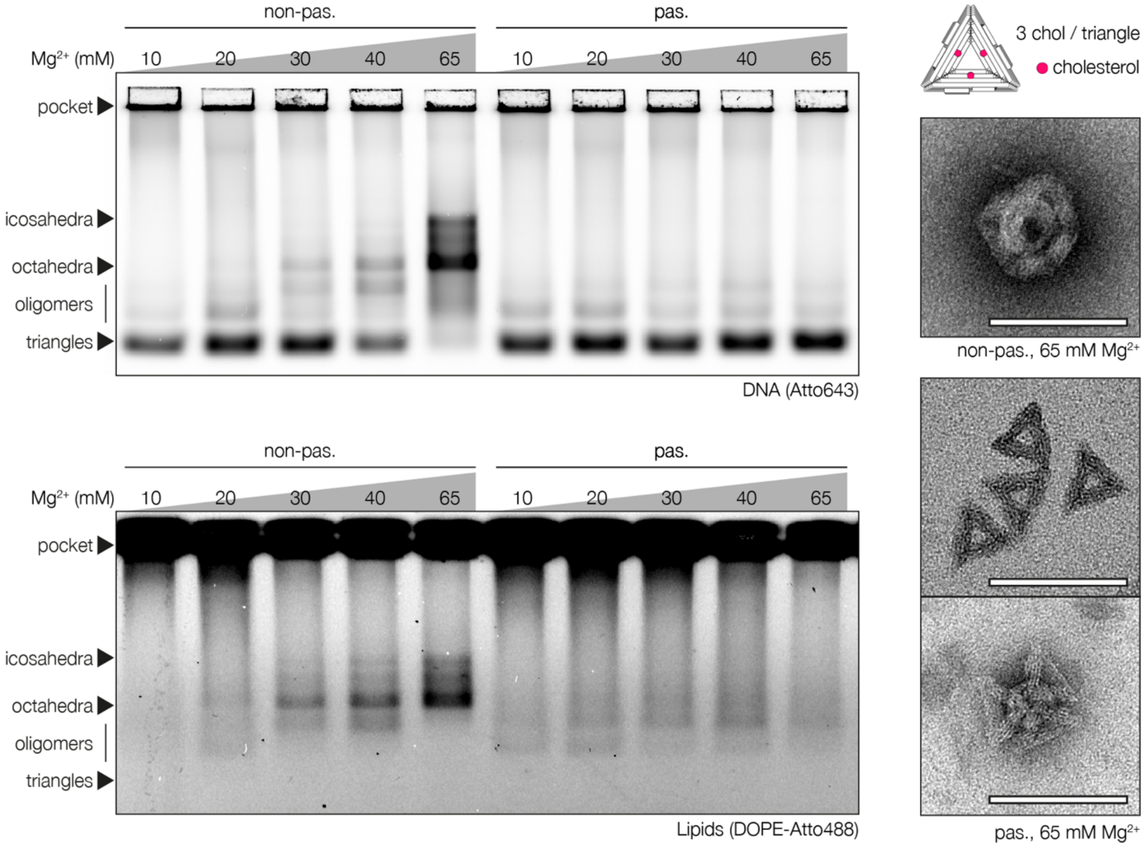

Supplementary Figure 15 | Triangle assembly is the driving force behind DCV formation. The importance of shell assembly for DCV formation was studied by incubating triangles at various $MgCl_2$ concentrations at (65 mM) or below (10-40 mM) the typical threshold concentration for shell assembly. For visualisation by agarose gel electrophoresis, the triangles were hybridised to Atto643 bearing oligonucleotides, and the GVs contained a fraction of Atto488 labelled DOPE. GVs were added at half the ideal amount for DCV formation to retain a fraction of free monomeric triangles. Two triangle species were prepared: A non-passivated version with blunt-ended stacking contacts and a passivated version where staple oligonucleotides at the base stacking contacts were extended by additional unpaired thymidines. Whereas the non-passivated versions formed DCVs, as seen by the bands in the lipid channel (lower panel) colocalising with the shells in the DNA channel (upper panel), only faint bands below the typical height of DNA shells were found in samples containing the passivated version. TEM analysis (right panels) revealed that while the passivated triangle samples contained mostly monomeric triangles, there was a small number of aggregated triangles held together by small vesicles, likely representing the faint bands. The lack of DCVs in samples containing passivated triangles highlights the importance of shell assembly for DCV formation. Scale bars: 100 nm. Source data are provided as a Source Data file.

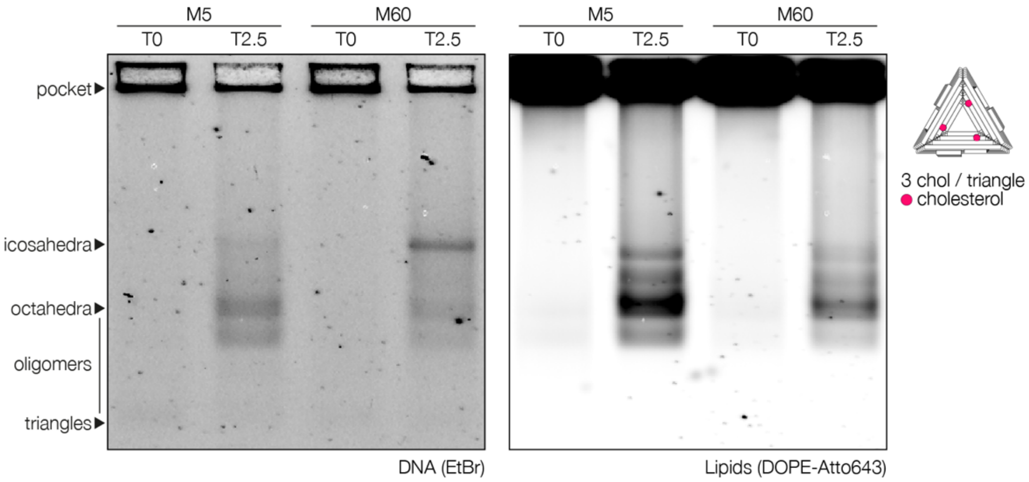

Supplementary Figure 16 | Shell preassembly reduces DCV yields. To assess how preassembled structures affect DCV formation, cholesterol-functionalized origami triangles were preincubated without GVs for 4 h at 37 °C in either low (5 mM) or high (60 mM) $MgCl_2$ conditions—promoting monomeric or assembled states, respectively. Following this, GVs containing a small fraction of DOPE-Atto643 were added. In the 5 mM condition, $MgCl_2$ was then adjusted to 60 mM to allow DCV formation. Samples were analysed by agarose gel electrophoresis at two timepoints: immediately after GV addition (t = 0 d), and after 2.5 d of incubation at 37 °C. At t = 0 d, both conditions showed similarly faint lipid band intensities, indicating comparable vesicle binding. However, after incubation, the 5 mM preincubation condition produced noticeably stronger lipid signals, suggesting higher DCV yields. High-Mg^{2+} preincubation favoured the formation of icosahedra, while the low-Mg^{2+} condition supported greater formation of octahedra upon membrane addition. The icosahedral band in the DNA channel of the sample preincubated at 60 mM Mg^{2+} does not translate into the lipid channel, indicating that these are empty shells and not DCVs. These observations support the idea that membrane-bound assembly alters the structural dynamics (see also Supplementary Figure 6) and is key for efficient DCV production. Reduced yields in the preassembled samples likely result from steric constraints that hinder binding of larger intermediates to vesicles or prevent the induction of sufficient curvature. Nonetheless, the detection of DCVs even in these conditions implies that small oligomers—such as dimers—can still engage with membranes and complete shell formation. Larger assemblies may also bind but fail to mature into buds due to limited curvature or monomer availability. Source data are provided as a Source Data file.

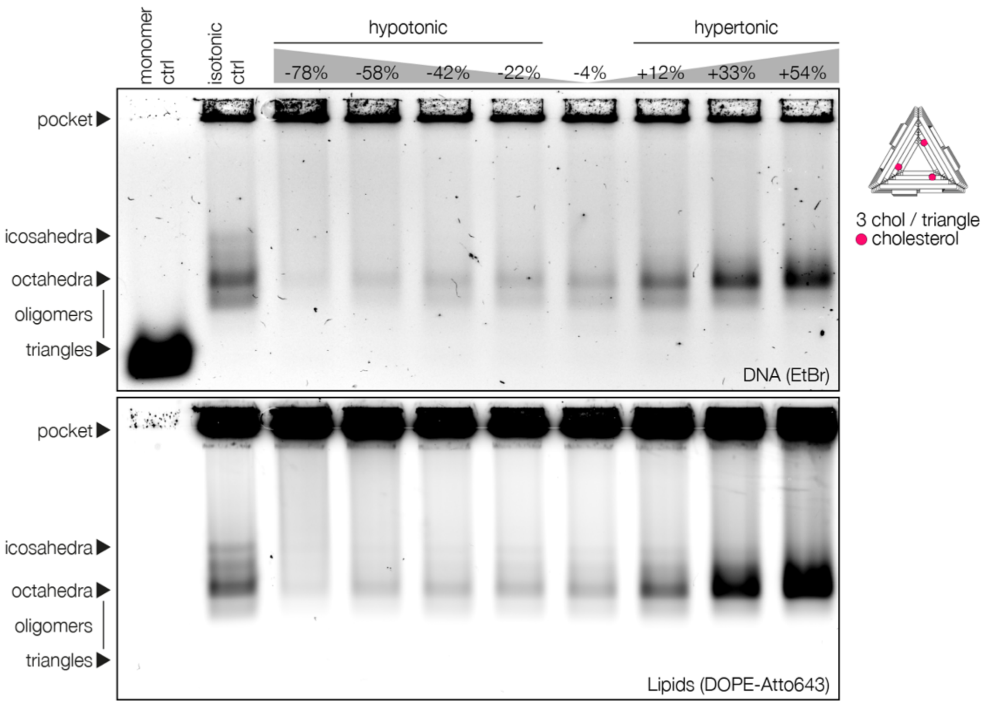

Supplementary Figure 17 | Budding assays at varying tonicities (channel-separated). Agarose gel of budding assays ran at different tonicities and visualised using two fluorophores. The top image shows signal obtained from DNA origami stained by ethidium bromide, while the bands in the bottom image stem from Atto643-labelled DOPE species included in the lipid mixture of the GVs. Budding efficiency strongly correlates with tonicity, working best under hypertonic conditions and coming to a near halt at the most hypotonic condition tested. Refer to Fig. 3a for a merged-colour image. Source data are provided as a Source Data file.

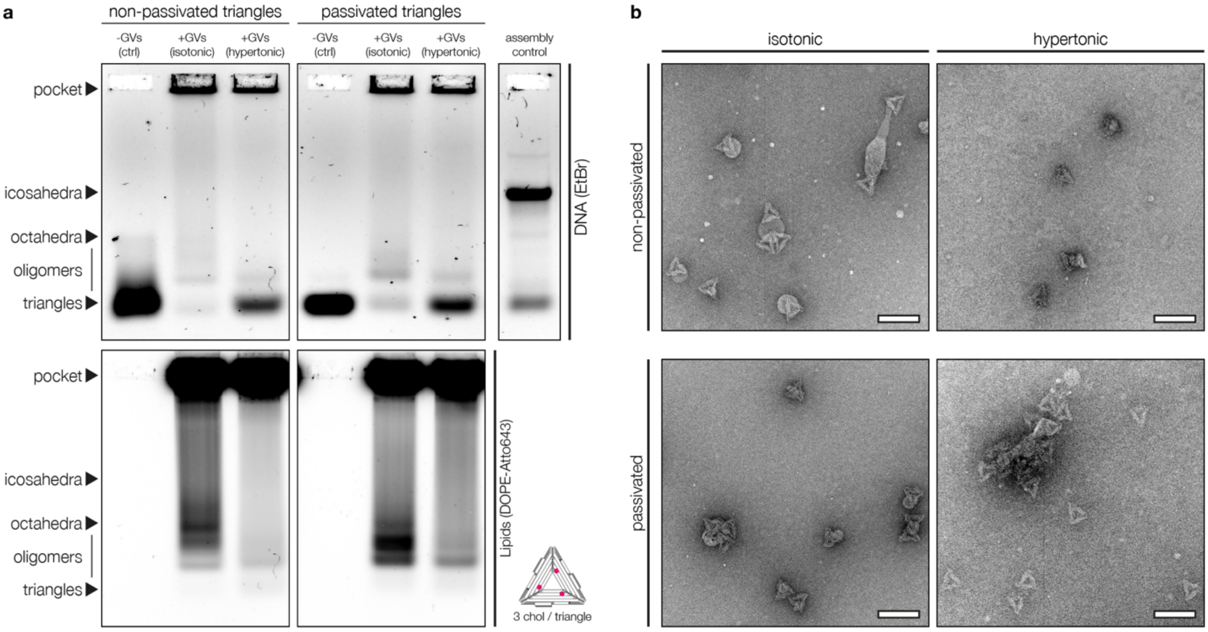

Supplementary Figure 18 | Effects of hypertonic conditions on membrane-bound triangles. a, Agarose gel of GV-bound triangles under isotonic (5 mM $MgCl_2$, 300 mM NaCl) or hypertonic (700 mM glycine, 31 mM NaCl, 5 mM $MgCl_2$) conditions. Both assembly-competent (left) and passivated triangles (middle) were tested and compared to a vesicle-free assembly control (right). Under hypertonic conditions, increased triangle signal in the gel and a stronger monomer band suggest reduced membrane coupling, possibly due to glycine interfering with chol-oligo insertion or sequestration by micelles. DNA-lipid hybrids appear as smears or bands in the lipid channel. **b,** TEM of the same samples. At isotonic conditions, triangles often bind to micelles, small vesicles, or membrane patches in a disordered fashion, without forming shells. At hypertonic conditions, triangles are mostly monomeric and membrane-detached, consistent with weakened chol-mediated binding. Budding under hypertonic conditions as discussed in Fig. 3a & Supplementary Figure 17 may thus partially rely on both avidity and electrostatic attraction between vesicles and DNA triangles to strengthen membrane-tethering in presence of glycine. Although this data suggests the possibility of lipid extraction by a non-budding mechanism, an artefactual origin appears equally likely. Scale bars: 100 nm. Source data are provided as a Source Data file.

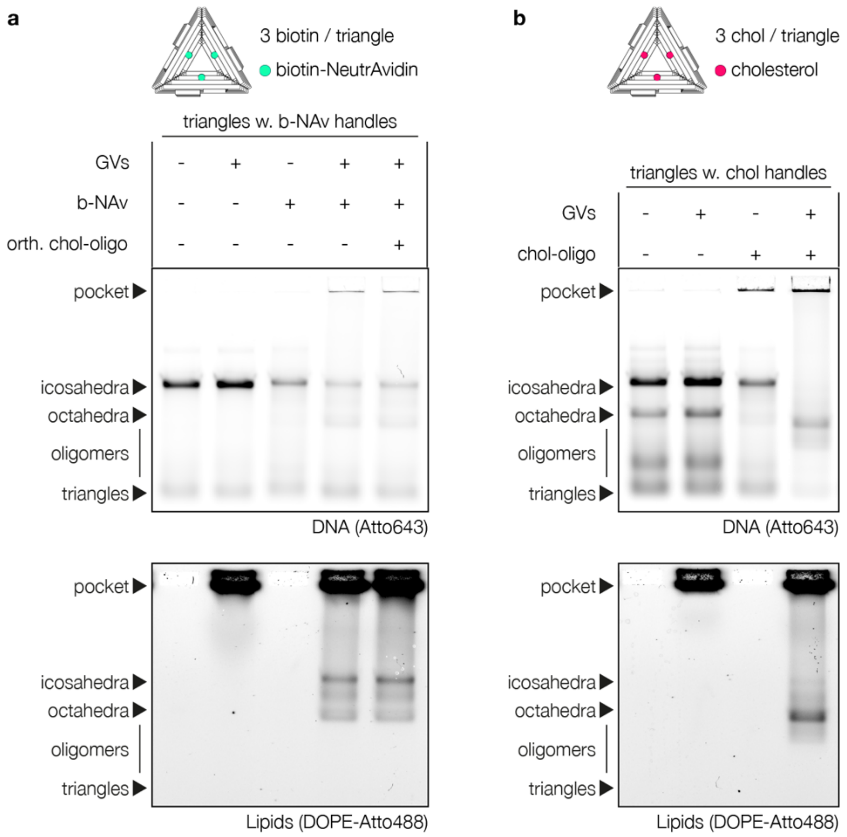

Supplementary Figure 19 | Membrane anchor screen (channel-separated). a, Agarose gel of DCVs prepared by using biotin-NeutrAvidin (b-NAv) interactions to connect origami triangles (Atto643, top image) and GVs (DOPE-Atto488, bottom image) containing a fraction of biotinylated DOPE. Linker handles on the triangles were complementary to b-NAv-oligos, but not to chol-oligos. Lipid material remains in the pockets unless triangles are mixed with b-NAv-oligos and GVs. The bands in the lipid channel colocalise with polyhedral shells but not with monomeric triangles, confirming the formation of DCVs. The addition of orthogonal chol-oligos did not improve yields, showing that the insertion of cholesterols into the bilayer is not a requirement. Gel pockets were oversaturated for better band visualisation. Refer to Fig. 3b for a merged-colour image. **b,** DCVs prepared under the same conditions and run on the same gel as in a, using triangles with linker handles complementary to chol-oligos. Comparison of shell band intensities in the lipid channel indicates that cholesterol-anchored triangles produce slightly higher overall DCV yields, while b-NAv anchoring results in a greater proportion of icosahedral species. Notably, the monomer band is much fainter in the chol-mediated reaction compared to b-NAv, suggesting lower vesicle coverage — or alternatively, less aggregation — in the b-NAv samples under otherwise identical conditions. Source data are provided as a Source Data file.

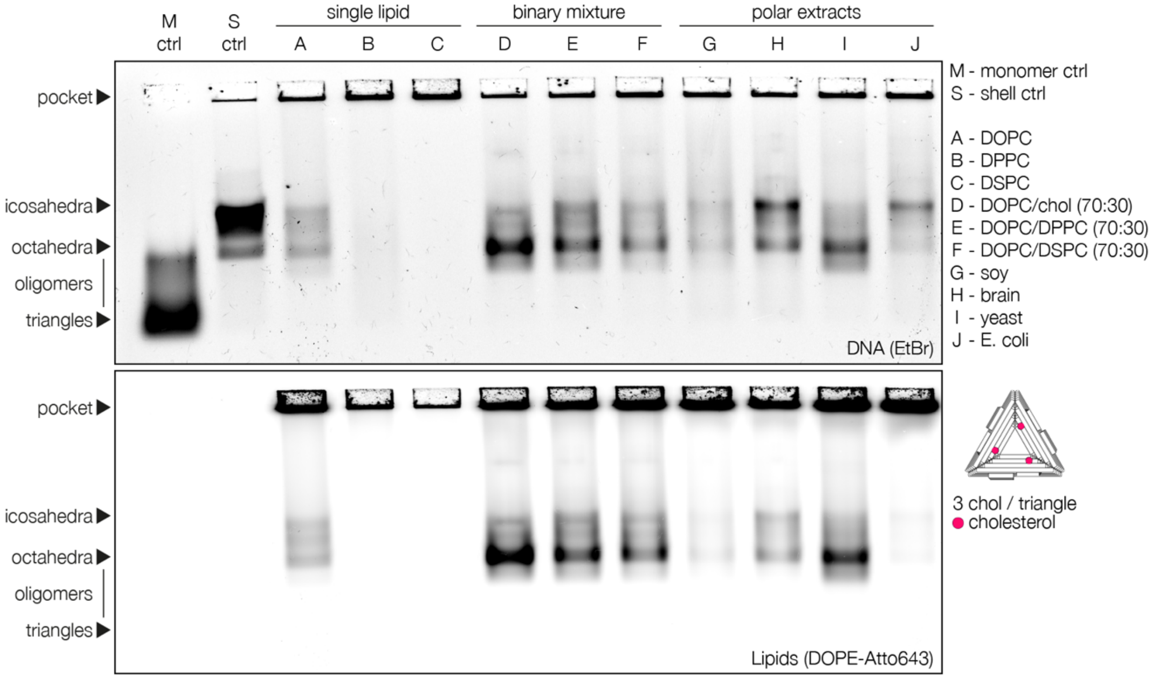

Supplementary Figure 20 | Budding assay using giant vesicles of varying lipid composition (channel-separated). Agarose gel of DCVs obtained from GVs of various lipid compositions. All compositions tested allowed budding of DCVs at varying efficiencies, except for GVs composed entirely of high-melting lipids. Low-grade assembly of the monomer ctrl is caused by the gel running conditions at 20 mM $MgCl_2$. Top image: DNA (EtBr); Bottom image: Lipids (DOPE-Atto643); Refer to Fig. 3c for a merged-colour image. Source data are provided as a Source Data file.

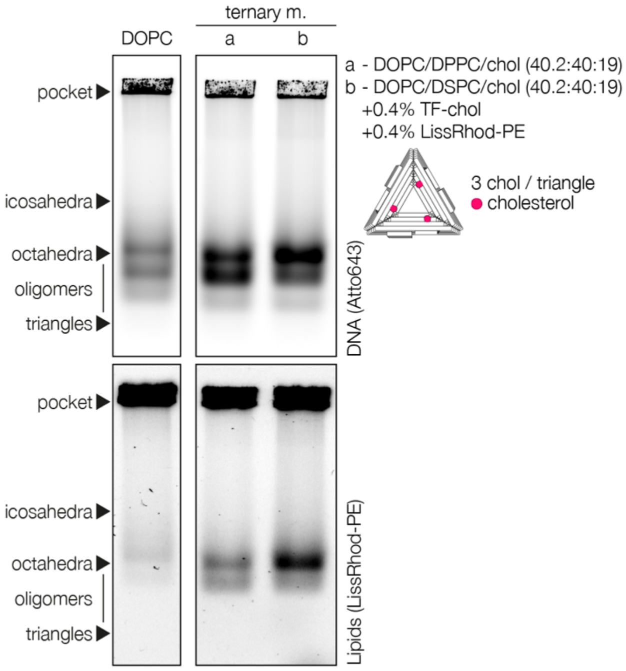

Supplementary Figure 21 | Budding assay from phase-separated vesicles (channel-separated). Agarose gel comparing DCVs obtained from GVs composed of DOPC or phase-separated ternary lipid mixtures. Phase-separated vesicles yielded more DCVs, suggesting an influence of phase boundaries on the budding mechanism. Top images: DNA (Atto643); Bottom images: Lipids (LissRhod-PE); Refer to Fig. 3d for a merged-colour image. Source data are provided as a Source Data file.

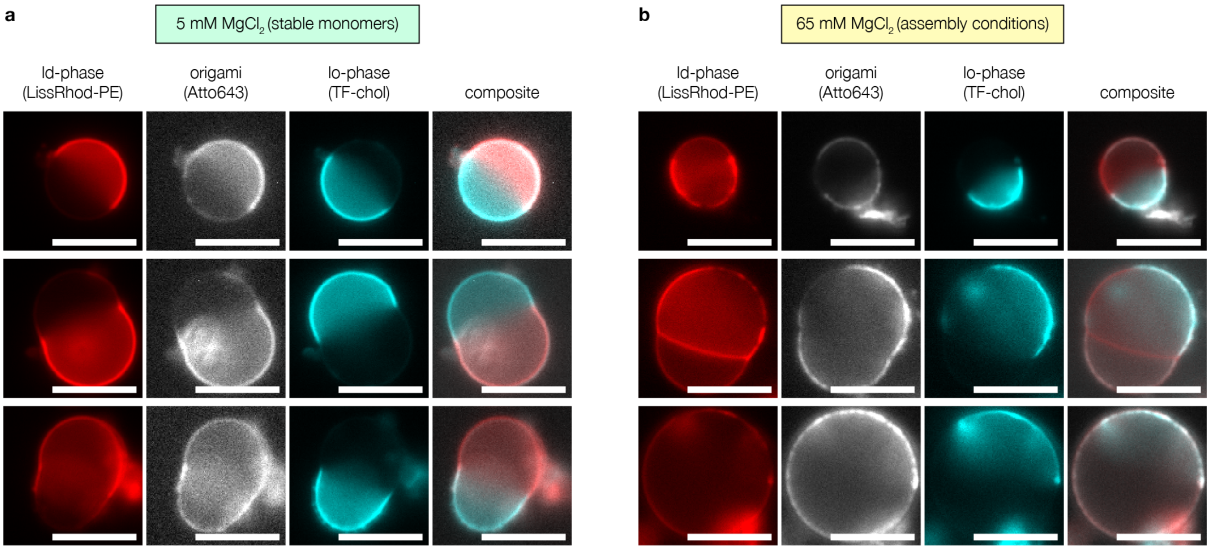

Supplementary Figure 22 | Membrane tethering of chol-functionalised triangles on phase-separated GVs as a function of $MgCl_2$ concentration. a, Fluorescence microscopy images of GVs with phase-separated membranes (red: ld-phase, LissRhod-PE; cyan: lo-phase, TF-chol) and DNA triangles (white, Atto643). At low $MgCl_2$ (5 mM), triangles preferentially (but not exclusively) localise to the ld-phase, consistent with previous reports[1]. **b,** At assembly conditions (65 mM $MgCl_2$), triangles increasingly localise to the lo-phase. Not all vesicles showed this reversal, possibly due to high triangle density hindering phase separation. For improved triangle visibility, the sample in b contained 5× more triangles than in a. Scale bars: 10 μm.

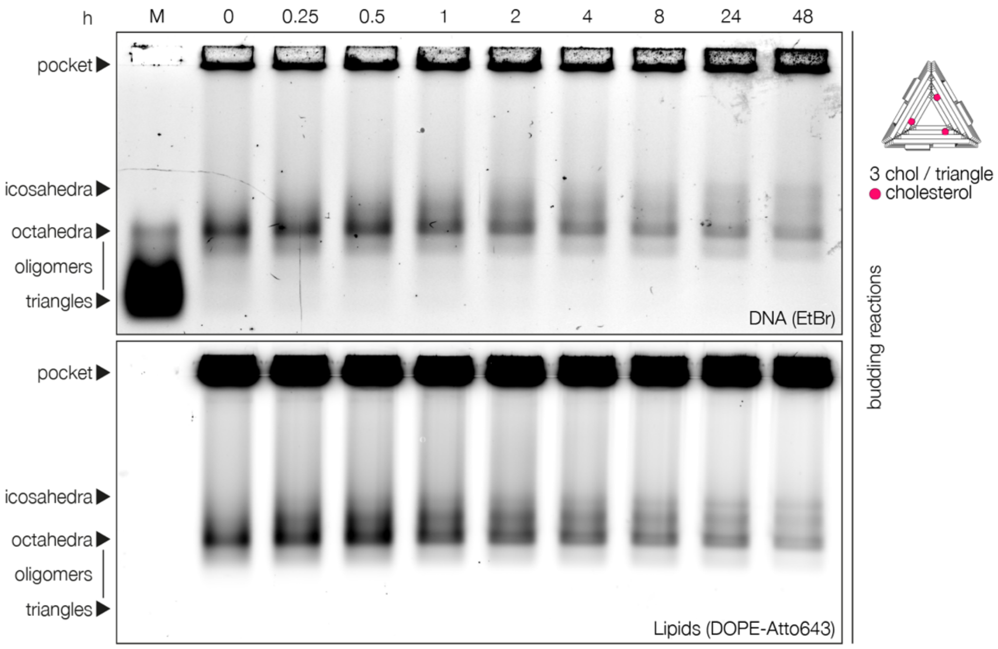

Supplementary Figure 23 | Budding kinetics (channel-separated). DCV budding occurs instantly and yields mostly octahedral species, but at longer incubation times a notable fraction of icosahedral DCVs is formed. Low-grade assembly of the monomer ctrl is caused by the gel running conditions at 20 mM $MgCl_2$. Top image: DNA (EtBr); Bottom image: Lipids (LissRhod-PE); Refer to Fig. 3e for a merged-colour image. Source data are provided as a Source Data file.

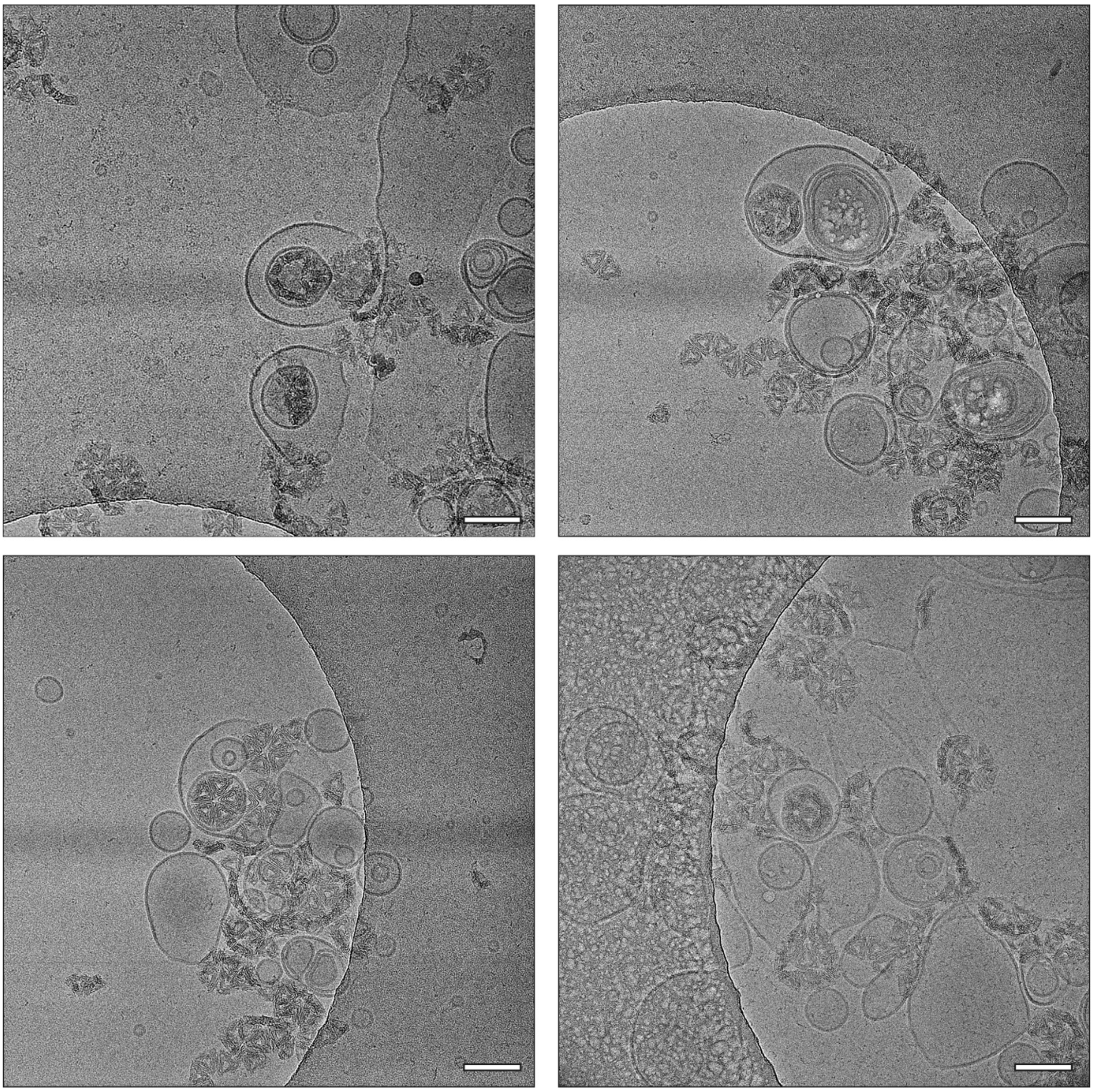

Supplementary Figure 24 | CryoEM images of VCDs, full fields-of-view. Scale bars: 100 nm.

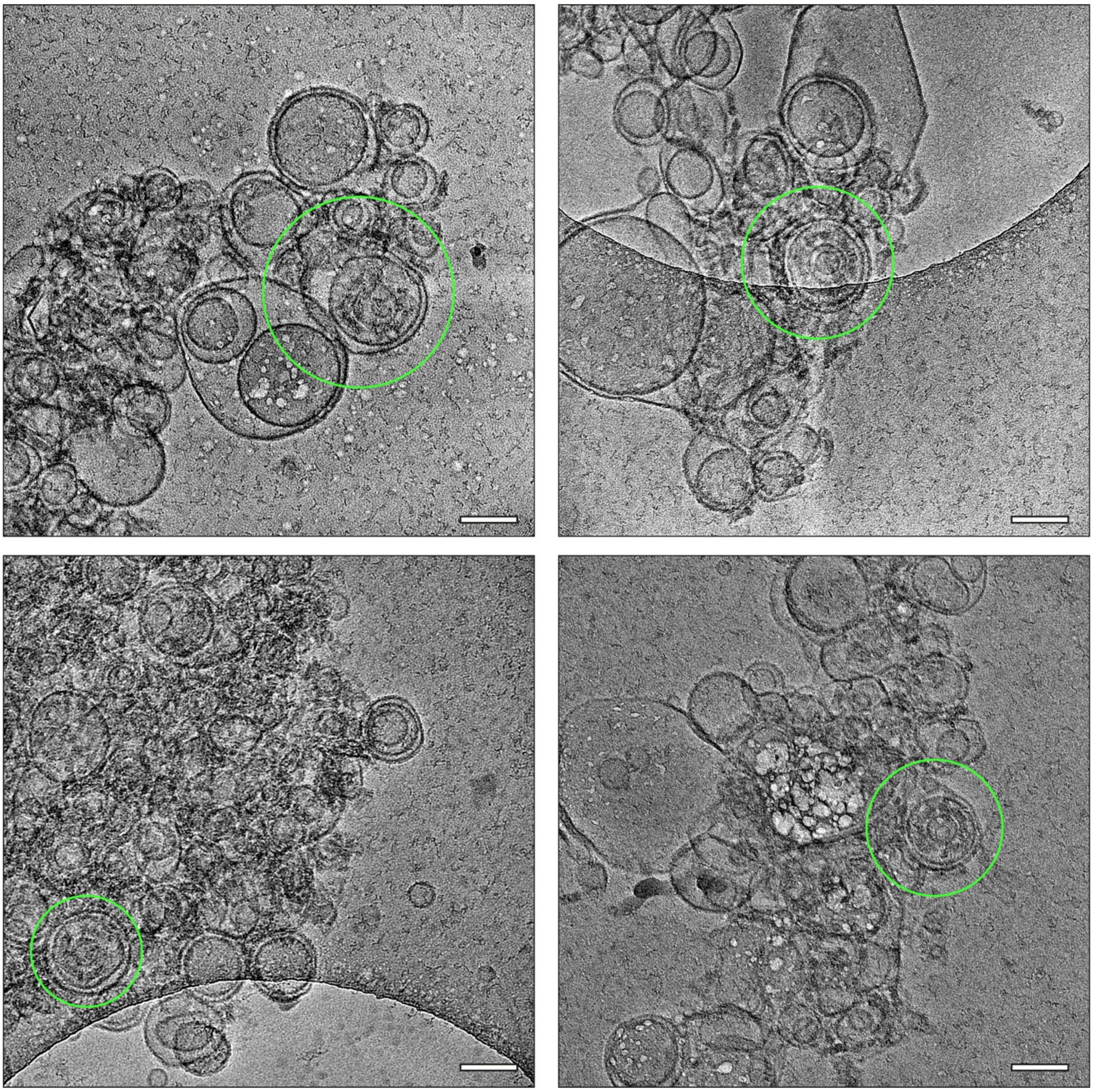

Supplementary Figure 25 | CryoEM images of VCDCVs, full fields-of-view. VCDCVs were produced by adding concentrated DCVs to LVs covered with chol-oligos integrated into them. Unlike VCD budding reactions, in which triangles are first bound to vesicles in a monomeric state, VCDCV budding requires mixing of DCVs and LVs at high magnesium conditions (60 mM) to ensure shell stability. These conditions promoted budding of DCVs into the freshly added, oligo-coated LVs, but also caused aggregation mediated by electrostatic interactions, DCVs binding to two LVs simultaneously, and pairing of residual single-stranded linker handles on the triangles, lowering the yield of VCDCVs. Optimisation of linker handles on the triangle surface and improving the DCV-LV coupling strategy may improve yields. Scale bars: 100 nm.

sc8064 sequence
GGCAATGACCTGATAGCCTTTGTAGATCTCTCAAAAATAGCTACCCTCTCCGGCATTAATTTATCAGCTAGAACGGTTGAATATCATATTGATGGTGATTTGACTGTCTCCG
GCCTTTCTCACCCTTTTGAATCTTTACCTACACATTACTCAGGCATTGCATTTAAAATATATGAGGGTTCTAAAAATTTTTATCCTTGCGTTGAAATAAAGGCTTCTCCCGC
AAAAGTATTACAGGGTCATAATGTTTTTGGTACAACCGATTTAGCTTTATGCTCTGAGGCTTTATTGCTTAATTTTGCTAATTCTTTGCCTTGCCTGTATGATTTATTGGAT
GTTAATGCTACTACTATTAGTAGAATTGATGCCACCTTTTCAGCTCGCGCCCCAAATGAAAATATAGCTAAACAGGTTATTGACCATTTGCGAAATGTATCTAATGGTCAAA
CTAAATCTACTCGTTCGCAGAATTGGGAATCAACTGTTATATGGAATGAAACTTCCAGACACCGTACTTTAGTTGCATATTTAAAACATGTTGAGCTACAGCATTATATTCA
GCAATTAAGCTCTAAGCCATCCGCAAAAATGACCTCTTATCAAAAGGAGCAATTAAAGGTACTCTCTAATCCTGACCTGTTGGAGTTTGCTTCCGGTCTGGTTCGCTTTGAA
GCTCGAATTAAAACGCGATATTTGAAGTCTTTCGGGCTTCCTCTTAATCTTTTTGATGCAATCCGCTTTGCTTCTGACTATAATAGTCAGGGTAAAGACCTGATTTTTGATT
TATGGTCATTCTCGTTTTCTGAACTGTTTAAAGCATTTGAGGGGGATTCAATGAATATTTATGACGATTCCGCAGTATTGGACGCTATCCAGTCTAAACATTTTACTATTAC
CCCCTCTGGCAAAACTTCTTTTGCAAAAGCCTCTCGCTATTTTGGTTTTTATCGTCGTCTGGTAAACGAGGGTTATGATAGTGTTGCTCTTACTATGCCTCGTAATTCCTTT
TGGCGTTATGTATCTGCATTAGTTGAATGTGGTATTCCTAAATCTCAACTGATGAATCTTTCTACCTGTAATAATGTTGTTCCGTTAGTTCGTTTTATTAACGTAGATTTTT
CTTCCCAACGTCCTGACTGGTATAATGAGCCAGTTCTTAAAATCGCATAAGGTAATTCACAATGATTAAAGTTGAAATTAAACCATCTCAAGCCCAATTTACTACTCGTTCT
GGTGTTTCTCGTCAGGGCAAGCCTTATTCACTGAATGAGCAGCTTTGTTACGTTGATTTGGGTAATGAATATCCGGTTCTTGTCAAGATTACTCTTGATGAAGGTCAGCCAG
CCTATGCGCCTGGTCTGTACACCGTTCATCTGTCCTCTTTCAAAGTTGGTCAGTTCGGTTCCCTTATGATTGACCGTCTGCGCCTCGTTCCGGCTAAGTAACATGGAGCAGG
TCGCGGATTTCGACACAATTTATCAGGCGATGATACAAATCTCCGTTGTACTTTGTTTCGCGCTTGGTATAATCGCTGGGGGTCAAAGATGAGTGTTTTAGTGTATTCTTTT
GCCTCTTTCGTTTTAGGTTGGTGCCTTCGTAGTGGCATTACGTATTTTACCCGTTTAATGGAAACTTCCTCATGAAAAAGTCTTTAGTCCTCAAAGCCTCTGTAGCCGTTGC
TACCCTCGTTCCGATGCTGTCTTTCGCTGCTGAGGGTGACGATCCCGCAAAAGCGGCCTTTAACTCCCTGCAAGCCTCAGCGACCGAATATATCGGTTATGCGTGGGCGATG
GTTGTTGTCATTGTCGGCGCAACTATCGGTATCAAGCTGTTTAAGAAATTCACCTCGAAAGCAAGCTGATAAACCGATACAATTAAAGGCTCCTTTTGGAGCCTTTTTTTTG
GAGATTTTCAACGTGAAAAAATTATTATTCGCAATTCCTTTAGTTGTTCCTTTCTATTCTCACTCCGCTGAAACTGTTGAAAGTTGTTTAGCAAAATCCCATACAGAAAATT
CATTTACTAACGTCTGGAAAGACGACAAAACTTTAGATCGTTACGCTAACTATGAGGGCTGTCTGTGGAATGCTACAGGCGTTGTAGTTTGTACTGGTGACGAAACTCAGTG
TTACGGTACATGGGTTCCTATTGGGCTTGCTATCCCTGAAAATGAGGGTGGTGGCTCTGAGGGTGGCGGTTCTGAGGGTGGCGGTTCTGAGGGTGGCGGTACTAAACCTCCT
GAGTACGGTGATACACCTATTCCGGGCTATACTTATATCAACCCTCTCGACGGCACTTATCCGCCTGGTACTGAGCAAAACCCCGCTAATCCTAATCCTTCTCTTGAGGAGT
CTCAGCCTCTTAATACTTTCATGTTTCAGAATAATAGGTTCCGAAATAGGCAGGGGGCATTAACTGTTTATACGGGCACTGTTACTCAAGGCACTGACCCCGTTAAAACTTA
TTACCAGTACACTCCTGTATCATCAAAAGCCATGTATGACGCTTACTGGAACGGTAAATTCAGAGACTGCGCTTTCCATTCTGGCTTTAATGAGGATTTATTTGTTTGTGAA
TATCAAGGCCAATCGTCTGACCTGCCTCAACCTCCTGTCAATGCTGGCGGCGGCTCTGGTGGTGGTTCTGGTGGCGGCTCTGAGGGTGGTGGCTCTGAGGGTGGCGGTTCTG
AGGGTGGCGGCTCTGAGGGAGGCGGTTCCGGTGGTGGCTCTGGTTCCGGTGATTTTGATTATGAAAAGATGGCAAACGCTAATAAGGGGGCTATGACCGAAAATGCCGATGA
AAACGCGCTACAGTCTGACGCTAAAGGCAAACTTGATTCTGTCGCTACTGATTACGGTGCTGCTATCGATGGTTTCATTGGTGACGTTTCCGGCCTTGCTAATGGTAATGGT
GCTACTGGTGATTTTGCTGGCTCTAATTCCCAAATGGCTCAAGTCGGTGACGGTGATAATTCACCTTTAATGAATAATTTCCGTCAATATTTACCTTCCCTCCCTCAATCGG
TTGAATGTCGCCCTTTTGTCTTTGGCGCTGGTAAACCATATGAATTTTCTATTGATTGTGACAAAATAAACTTATTCCGTGGTGTCTTTGCGTTTCTTTTATATGTTGCCAC
CTTTATGTATGTATTTTCTACGTTTGCTAACATACTGCGTAATAAGGAGTCTTAATCATGCCAGTTCTTTTGGGTATTCCGTTATTATTGCGTTTCCTCGGTTTCCTTCTGG
TAACTTTGTTCGGCTATCTGCTTACTTTTCTTAAAAAGGGCTTCGGTAAGATAGCTATTGCTATTTCATTGTTTCTTGCTCTTATTATTGGGCTTAACTCAATTCTTGTGGG
TTATCTCTCTGATATTAGCGCTCAATTACCCTCTGACTTTGTTCAGGGTGTTCAGTTAATTCTCCCGTCTAATGCGCTTCCCTGTTTTTATGTTATTCTCTCTGTAAAGGCT
GCTATTTTCATTTTTGACGTTAAACAAAAAATCGTTTCTTATTTGGATTGGGATAAATAATATGGCTGTTTATTTTGTAACTGGCAAATTAGGCTCTGGAAAGACGCTCGTT
AGCGTTGGTAAGATTCAGGATAAAATTGTAGCTGGGTGCAAAATAGCAACTAATCTTGATTTAAGGCTTCAAAACCTCCCGCAAGTCGGGAGGTTCGCTAAAACGCCTCGCG
TTCTTAGAATACCGGATAAGCCTTCTATATCTGATTTGCTTGCTATTGGGCGCGGTAATGATTCCTACGATGAAAATAAAAACGGCTTGCTTGTTCTCGATGAGTGCGGTAC
TTGGTTTAATACCCGTTCTTGGAATGATAAGGAAAGACAGCCGATTATTGATTGGTTTCTACATGCTCGTAAATTAGGATGGGATATTATTTTTCTTGTTCAGGACTTATCT
ATTGTTGATAAACAGGCGCGTTCTGCATTAGCTGAACATGTTGTTTATTGTCGTCGTCTGGACAGAATTACTTTACCTTTTGTCGGTACTTTATATTCTCTTATTACTGGCT
CGAAAATGCCTCTGCCTAAATTACATGTTGGCGTTGTTAAATATGGCGATTCTCAATTAAGCCCTACTGTTGAGCGTTGGCTTTATACTGGTAAGAATTTGTATAACGCATA
TGATACTAAACAGGCTTTTTCTAGTAATTATGATTCCGGTGTTTATTCTTATTTAACGCCTTATTTATCACACGGTCGGTATTTCAAACCATTAAATTTAGGTCAGAAGATG
AAATTAACTAAAATATATTTGAAAAAGTTTTCTCGCGTTCTTTGTCTTGCGATTGGATTTGCATCAGCATTTACATATAGTTATATAACCCAACCTAAGCCGGAGGTTAAAA
AGGTAGTCTCTCAGACCTATGATTTTGATAAATTCACTATTGACTCTTCTCAGCGTCTTAATCTAAGCTATCGCTATGTTTTCAAGGATTCTAAGGGAAAATTAATTAATAG
CGACGATTTACAGAAGCAAGGTTATTCACTCACATATATTGATTTATGTACTGTTTCCATTAAAAAAGGTAATTCAAATGAAATTGTTAAATGTAATTAATTTTGTTTTCTT
GATGTTTGTTTCATCATCTTCTTTTGCTCAGGTAATTGAAATGAATAATTCGCCTCTGCGCGATTTTGTAACTTGGTATTCAAAGCAATCAGGCGAATCCGTTATTGTTTCT
CCCGATGTAAAAGGTACTGTTACTGTATATTCATCTGACGTTAAACCTGAAAATCTACGCAATTTCTTTATTTCTGTTTTACGTGCAAATAATTTTGATATGGTAGGTTCTA
ACCCTTCCATTATTCAGAAGTATAATCCAAACAATCAGGATTATATTGATGAATTGCCATCATCTGATAATCAGGAATATGATGATAATTCCGCTCCTTCTGGTGGTTTCTT
TGTTCCGCAAAATGATAATGTTACTCAAACTTTTAAAATTAATAACGTTCGGGCAAAGGATTTAATACGAGTTGTCGAATTGTTTGTAAAGTCTAATACTTCTAAATCCTCA
AATGTATTATCTATTGACGGCTCTAATCTATTAGTTGTTAGTGCTCCTAAAGATATTTTAGATAACCTTCCTCAATTCCTTTCAACTGTTGATTTGCCAACTGACCAGATAT
TGATTGAGGGTTTGATATTTGAGGTTCAGCAAGGTGATGCTTTAGATTTTTCATTTGCTGCTGGCTCTCAGCGTGGCACTGTTGCAGGCGGTGTTAATACTGACCGCCTCAC
CTCTGTTTTATCTTCTGCTGGTGGTTCGTTCGGTATTTTTAATGGCGATGTTTTAGGGCTATCAGTTCGCGCATTAAAGACTAATAGCCATTCAAAAATATTGTCTGTGCCA
CGTATTCTTACGCTTTCAGGTCAGAAGGGTTCTATCTCTGTTGGCCAGAATGTCCCTTTTATTACTGGTCGTGTGACTGGTGAATCTGCCAATGTAAATAATCCATTTCAGA
CGATTGAGCGTCAAAATGTAGGTATTTCCATGAGCGTTTTTCCTGTTGCAATGGCTGGCGGTAATATTGTTCTGGATATTACCAGCAAGGCCGATAGTTTGAGTTCTTCTAC
TCAGGCAAGTGATGTTATTACTAATCAAAGAAGTATTGCTACAACGGTTAATTTGCGTGATGGACAGACTCTTTTACTCGGTGGCCTCACTGATTATAAAAACACTTCTCAG
GATTCTGGCGTACCGTTCCTGTCTAAAATCCCTTTAATCGGCCTCCTGTTTAGCTCCCGCTCTGATTCTAACGAGGAAAGCACGTTATACGTGCTCGTCAAAGCAACCATAG
TACGCGCCCTGTAGCGGCGCATTAAGCGCGGCGGGTGTGGTGGTTACGCGCAGCGTGACCGCTACACTTGCCAGCGCCCTAGCGCCCGCTCCTTTCGCTTTCTTCCCTTCCT
TTCTCGCCACGTTCGCCGGCTTTCCCCGTCAAGCTCTAAATCGGGGGCTCCCTTTAGGGTTCCGATTTAGTGCTTTACGGCACCTCGACCCCAAAAAACTTGATTTGGGTGA
TGGTTCACGTAGTGGGCCATCGCCCTGATAGACGGTTTTTCGCCCTTTGACGTTGGAGTCCACGTTCTTTAATAGTGGACTCTTGTTCCAAACTGGAACAACACTCAACCCT
ATCTCGGGCTATTCTTTTGATTTATAAGGGATTTTGCCGATTTCGGAACCACCATCAAACAGGATTTTCGCCTGCTGGGGCAAACCAGCGTGGACCGCTTGCTGCAACTCTC
TCAGGGCCAGGCGGTGAAGGGCAATCAGCTGTTGCCCGTCTCACTGGTGAAAAGAAAAACCACCCTGGCGCCCAATACGCAAACCGCCTCTCCCCGCGCGTTGGCCGATTCA
TTAATGCAGCTGGCACGACAGGTTTCCCGACTGGAAAGCGGGCAGTGAGCGCAACGCAATTAATGTGAGTTAGCTCACTCATTAGGCACCCCAGGCTTTACACTTTATGCTT
CCGGCTCGTATGTTGTGTGGAATTGTGAGCGGATAACAATTTCACACAGGAAACAGCTATGACCATGATTACGAATTCGAGCTCGGTACCCGGGGATCCTCAACTGTGAGGA
GGCTCACGGACGCGAAGAACAGGCACGCGTGCTGGCAGAAACCCCCGGTATGACCGTGAAAACGGCCCGCCGCATTCTGGCCGCAGCACCACAGAGTGCACAGGCGCGCAGT
GACACTGCGCTGGATCGTCTGATGCAGGGGGCACCGGCACCGCTGGCTGCAGGTAACCCGGCATCTGATGCCGTTAACGATTTGCTGAACACACCAGTGTAAGGGATGTTTA
TGACGAGCAAAGAAACCTTTACCCATTACCAGCCGCAGGGCAACAGTGACCCGGCTCATACCGCAACCGCGCCCGGCGGATTGAGTGCGAAAGCGCCTGCAATGACCCCGCT
GATGCTGGACACCTCCAGCCGTAAGCTGGTTGCGTGGGATGGCACCACCGACGGTGCTGCCGTTGGCATTCTTGCGGTTGCTGCTGACCAGACCAGCACCACGCTGACGTTC
TACAAGTCCGGCACGTTCCGTTATGAGGATGTGCTCTGGCCGGAGGCTGCCAGCGACGAGACGAAAAAACGGACCGCGTTTGCCGGAACGGCAATCAGCATCGTTTAACTTT
ACCCTTCATCACTAAAGGCCGCCTGTGCGGCTTTTTTTACGGGATTTTTTTATGTCGATGTACACAACCGCCCAACTGCTGGCGGCAAATGAGCAGAAATTTAAGTTTGATC
CGCTGTTTCTGCGTCTCTTTTTCCGTGAGAGCTATCCCTTCACCACGGAGAAAGTCTATCTCTCACAAATTCCGGGACTGGTAAACATGGCGCTGTACGTTTCGCCGATTGT
TTCCGGTGAGGTTATCCGTTCCCGTGGCGGCTCCACCTCTGAAAGCTTGGCACTGGCCGTCGTTTTACAACGTCGTGACTGGGAAAACCCTGGCGTTACCCAACTTAATCGC
CTTGCAGCACATCCCCCTTTCGCCAGCTGGCGTAATAGCGAAGAGGCCCGCACCGATCGCCCTTCCCAACAGTTGCGCAGCCTGAATGGCGAATGGCGCTTTGCCTGGTTTC
CGGCACCAGAAGCGGTGCCGGAAAGCTGGCTGGAGTGCGATCTTCCTGAGGCCGATACTGTCGTCGTCCCCTCAAACTGGCAGATGCACGGTTACGATGCGCCCATCTACAC
CAACGTGACCTATCCCATTACGGTCAATCCGCCGTTTGTTCCCACGGAGAATCCGACGGGTTGTTACTCGCTCACATTTAATGTTGATGAAAGCTGGCTACAGGAAGGCCAG
ACGCGAATTATTTTTGATGGCGTTCCTATTGGTTAAAAAATGAGCTGATTTAACAAAAATTTAATGCGAATTTTAACAAAATATTAACGTTTACAATTTAAATATTTGCTTA
TACAATCTTCCTGTTTTTGGGGCTTTTCTGATTATCAACCGGGGTACATATGATTGACATGCTAGTTTTACGATTACCGTTCATCGATTCTCTTGTTTGCTCCAGACTCTCA

Supplementary Table 1 | Sequence of scaffold sc8064.

experiment	reference figure	core mix	internal linker mix	external linker mix	internal funct. strands	external funct. strands
Vesicle deformation & buds in cryoEM; octahedral DCVs	Fig. 1D S. Fig. 9, 10	C1	I1	E1	CHOL1	*no linkers*
DCVs from triangles with 3/6/9 cholesterols	Fig. 2A & S. Fig. 11	C1	I10 or I11 or I12	E6	CHOL4	FLUO1
DNA-lipid colocalisation in agarose gel electrophoresis	Fig. 2B	C1	I10	E6	CHOL4	FLUO1
Budding by tonicity	Fig. 3A & S. Fig. 17	C1	I1	E1	CHOL1	*no linkers*
Budding by membrane anchor (b-NAv)	Fig. 3B & S. Fig. 19A	C1	I3	E2	BIOTIN	FLUO1 & bead purification linkers
Budding by lipid mixture	Fig. 3C & S. Fig. 20	C1	I1	E1	CHOL1	*no linkers*
Budding from phase-separated vesicles	Fig. 3D & S. Fig. 21	C1	I1	E3	CHOL1	*FLUO2*
Budding kinetics	Fig. 3E & S. Fig. 23	C1	I1	E1	CHOL1	*no linkers*
Inward budding	Fig. 4B, C, S. Fig. 24	C1	I4	E4	*no linkers*	CHOL1
Bivesicular budding	Fig. 5B, S. Fig. 25	C1	I1	E5	CHOL1	CHOL2 & FLUO1*
Cholesterol position screen	S. Fig. 3	C1	I5 or I6	E1	CHOL2 or CHOL3	*no linkers*
Cholesterol count screen	S. Fig. 4	C1	I7 or I8 or I9	E6	CHOL4	FLUO1
Influence of GVs on triangle assembly	S. Fig. 5	C1	I10 or I11 or I12	E6	CHOL4	FLUO1
Influence of GVs on triangle assembly 2	S. Fig. 6	C1	I10	E6	CHOL4	FLUO1
Budding by $MgCl_2$ concentration	S. Fig. 12	C1	I1	E1	CHOL1	*no linkers*
Dialysis of DCVs into low magnesium buffer	S. Fig. 13	C1	I1	E1	CHOL1	*no linkers*
Budding by vesicle size & lipid quantity	S. Fig. 14	C1	I1	E1	CHOL1	*no linkers*
Assembly as the driving force for DCV budding	S. Fig. 15	C1 or C2	I2	E3	CHOL2	FLUO2
Preassembled shells	S. Fig. 16	C1	I1	E1	CHOL1	*no linkers*
Monomeric triangles on GVs in iso-/hypertonic buffer	S. Fig. 18	C1 or C2	I1	E1	CHOL1	*no linkers*
Budding by membrane anchor (chol.)	S. Fig. 19B	C1	I2	E2	CHOL2	FLUO1 & bead purification linkers
Biased triangle distribution on phase-separated vesicles	S. Fig. 22	C1	I1	E3	CHOL1	*FLUO2*

Supplementary Table 2 | Triangle variants used in this study. For different experiments (see ‘experiment’ and ‘reference figure’ columns), slight variations of the triangular origami structure have been used, differing mostly in the sequence, number and positioning of its linker handles. For simplicity, the final staple mixture was divided into three sub-mixtures comprising a constant part (core mix), staples with linker handle extensions on the shell-inner face (internal linker mix), and staples with linker handle extensions on the shell-outer face (external linker mix). For foldings, these mixtures were mixed according to their staple count (equal staple concentrations in the final mixture). The corresponding complementary functionalised oligonucleotides (see Supplementary Table 3 for sequences), if any, for both faces are listed in additional columns. For experiments not listed, any triangle variant fulfilling the experimental needs (e.g. type of membrane anchor, fluorescent labelling, etc.) can be used. Note that cholesterol-bearing strands were not included in folding reactions but added just before mixing with vesicle solutions (see Methods section). Refer to the following supplementary tables for sequences. *Linker handles for FLUO1 were left single-stranded (no fluorescence labelling was required).

functionalised strands	sequence	modification
CHOL1	CTCCACCTCTCACTCTTT	3'-(cholesterol-TEG)
CHOL2	ATCATCTCACCTTTT	3'-(cholesterol-TEG)
CHOL3	TTTTCCACTCTACTA	5'-(cholesterol-TEG)
CHOL4	ATCATCTCACCTTTT	3'-(cholesterol-prolinol)
FLUO1	CCAAGCTACAGCCTGAAA	3'-(Atto643)
FLUO2	GCCAGTCCGCCTCGCAAA	3'-(Atto643)
BIOTIN	TACGCCGGAAGCAGC	3'-(biotin-TEG)
purification linker handle	*see Supplementary Table 4*	

Supplementary Table 3 | List of functionalised strands.

Strand	sequence	modification
Bead handle	TTTCTATCACCTCCA	5'-(dual biotin)
Origami handle	--TTTGATGTGTAGAGTTGGAGGTGATAG	
Invader strand	CCTTCAATCTTACTATCACCTCCAACTCTACACATC	

Supplementary Table 4 | List of sequences for magnetic bead purification of DNA origami. Bead handles are coupled to Dynabeads M-270 Streptavidin. The stated sequence of the origami handle is part of a staple with the full sequences listed in Supplementary Table 20. Complementary sequence snippets are highlighted. The invader strands can optionally be removed from the origami by its reverse complement.

No.	C1 sequences (non-passivated triangles)
01	GGAAACCACGGTGCGGGCCTCTTCAGCCCAATGTATAAGCAAAAGCCC
02	GCTATTACTAAAATTCGCATTGCTTTAAACAGTT
03	CGCCACCCTCAGAACCGGAATAGCGCAATAATAGGACTGTAGCGCGT
04	AATACCCAACGGAATACAAGATAGCAGCACCG
05	AGTGTACCCCGGTTGCTGCTCCAATTTTCAGGGATAGCA
06	TGTTACTTAGCCGCATTAGACGGAAGACACCAAAGAACT
07	TTAATGCCAGAATCGAGAATATAAACGGTGTACCAACTTTGAAAGAGG
08	GCAGACGGTCAATCATTCCGCGACATAATCAGAAATATTTCTCAGAG
09	AAAATAGCAAAGTCAGAGGAGAAAACTTTTTC
10	AGCCCGAGGAAGATTAGGAACCCATAAATTGTGTCGAAAAAGGGAAC
11	GCCGAACAAAGTTACCAGTAAGACTCAGAGAGATACTGAACA
12	GCCTTTAGCGTCACAAAGGATAAAAATTTTTAGAACCCT
13	TCAAGTTTGGCATGATAAGGAAACAGAGCAAGTTTAGTACACGTTGT
14	AATAAAGCAAATTGTAAACGCGGCCACCCTC
15	TTTTGTCAGGATATCAGGTCATTGCCTGAGAACCCAGCT
16	CCAGTAGCACCGTCACCGACTTGAAACGCTAAATAACATA
17	TGGGTTATATGGAAACAGTACATACCAAGCTTTCAGAGGTGGAGCCG
18	TGTCAATCATATTTAAACGAGGCACAATTTTTTAGTTGC
19	AGAACGCGAGGCGTTTTAGCGAACGGCAGAGG
20	GTCTGGAGAACTAGCAGACAGTCATGTAATACTTTTGAGG
21	CTTGCGGGAGGTTTTGAAGCCTTAAAAAGCCTAAAACAGGGAAGCGG
22	TATTTTGCACCTTCATCAAGAGTAGCGCATAGGCTGGCTG
23	CAAAAATGCATTTTCGTTTGAAATACCGACCGGCTTAGGTATTGAGCG
24	CATATATTAATCAGTAGCGAGGAACCGCCT
25	ACCCCCAGCGATTATATTACCCAAATTCATCAACAGATGA
26	AGCCTTTATTTCAACGCAAATTAAGCTAAAGATT
27	TTTAAATGCAATGCCTGAGTAATGTGTCGGGAGA
28	ATATAAAGTATTTTTCGACAAAAGTTCATTTTTCTTCTGACCTAAA
29	TTAAAGGTGCCAAAGACAAAAGGGCTTTTTACATTCAACCGATTGA
30	AGGTAAATATTGTTTTTCGGAAATTATTCATGCCAGTT
31	TTATTTATCCCCCAATAGCAAGCAAATCAGATTTTTTTAGAAGGCTTATCCG
32	GCTAATGCAGAACGCGCATCAAGAAAACACAAGACTTTTTAAGAACGCGGTA
33	CCACGGGATTTCATTTGATTTTTTTACCTTTTTTAATAACTAT
34	TTTCATCGGCATTTTTTTTCGGACCAGAGCCTTTTTCCACCCAGAA
35	AAATATCAATAAGAAACGATTTTTTTTTTGTTTAACGTACAAAATA
36	AGCCGCCGCAGGTCAGACGATTGGCTTTTTTTGACATTAGCAAGGCCGGA
37	AACGTAGAAAATTTTTACATACATAAAGGCAGTAT
38	ATGTAAATTTTTTCTGATGCAAATCCAATGTTATTTTAGTTAAT
39	GCTCATGGAGCACCAGCAGAAGATAAAACAGAGGTGACAAATCAACA
40	GTAAAGTAATTCTGTCTTACCGCGCAATCCAACATTCCAA
41	TCACCCAAGAGAACAAGCAAGCCGAAGTACCGCACTCATC
42	AATGCGCGAAGAATACGTGGCACAGCACTAAAGCGCGTAA
43	GCCAGCGGGAGGTGTCCAGCATCAATCCCGTAAAAAAAGCCGCACAG
44	TCGGCGAACTCCGTGGTGAAGGGAAAGATGATAACCTATTGTTAATGC
45	TGATTGCTTTGAATAGTACCTTTCCGTTGTAGCCACAGTGCCACGCT
46	CCCTCAGTTTTCATACCCTCAGAAAATAACAGTGCCCGCCTGAACAATTACC
47	TGAGCAAAAGTAGCTCTCACTCCTCAAGAGAAAGTAAGCAGATA
48	AACCACCACCAGGCCACCA
49	AGAAAAATAATCCGCGCTTAATGGGAAAAACATTAGTAA
50	ATTTACGAGCATGTAGAGATAAGTTATAAACAATTCTGAACACCGGA
51	GCTGGCAAGTTGCTTTGACTGCACTCTGTGGT
52	GCTAGGGCAGCTAACTGGTTTCTGCCAGCACGTACCTGCACACGTATA
53	TCAGTATTTAACATCACCATCACGTTAAAGGGGATTCGCCGTTGTGT
54	AAAAACCGTCTATCAGGGCGATGGCCGAGCTCCCACCACACCCGATC
55	TCCTCATTAGGAGTGTGGGAGGGAGAACGGGTGGCTGTCTTTCCTTAT
56	TGGCTTTTAACGGGGTAGGTTGAGACCCTCAGAACCGCCA
57	TAAAGAACGTGGACTCCAACGTCAAATGAGTG
58	CAGTGCCTTGAGTACCCATCCTATTTTGGGGGTGAACCA
59	CTACATTTTTTGTTCCAGTAAGCGTCATACAATCAAGTT
60	ATCAAAATACATGAAAGTATATATCATTTCA
61	AACACCGCCTGCAATCAGCCGCCACCAG
62	GTTAGCATATTTCGGGAAACAAACCTGTTTATCAACAATAAACCAAT
63	CCGAGTAAAAGAGTCTGTCTTGCCTGTCCTCGTTCAGGGCGC
64	GCCTTGCTGGTTTTTTATATCCAGAACAAACTCAA
65	GCGCGGGGAGAGGCGGGAATGCCAACGGCTGTCACTTTTTGCGCGCCTGGAG
66	GCGGCCTTCCCACGCAACTTTTTAGCTTACGGCTGTGCCGGTG
67	CCCCCTGCTTTTTTCAGACGATCCAGCGGCCGGCCAGAATGCGG
68	GCTGCAACAAGGGAAGAAAGTTTTTGAAAGGAGCGGGCCTTGACGG
69	GGAATTGGTCAATAGATAATACATTTTTTTGAGGGCCCTAAAACATCGCC
70	CGCTCACTGCTTTTTCGCTTTCCACGGGTTTTTCGTTTTCACGGTC
71	CGAACGTGGCGAGATAGGGTTGAGTGTTGTTCTTTTTAGTTTGGAACAAGAG
72	GAGAGCCAGCAGCTTTTTAATGTATCTGGTCTTTTTGTTGGGGCGG
73	TCTGACCTATTGGCAGATTCACCAGTTTTTCACACGACCAGTAATA
74	ATTCTGGCCAACTTTTTGAGATAGAACCCTATTTAGAG
75	AACTCCAAAGGTGTTTAGCTATATTTTCATTTGGGGCGTTGTACCAA
76	CAGTTCCTGATTATCTTTTTCTTCGTGGTGCTGGTCTGG
77	CGAGAAACACCAGAACGAGTAGTACACCAACC
78	ACTAATGCTAAAACGACGATATATTCGGTCGCAAAAGGCTGAGGCTTT
79	AGTAACATGGAGCGGATTAGAGCCAGGAAGGTTATCTAAA
80	AATAAAGACGTGGGAATGCCGGACTTGATGTTTAGACTG

81	CAGAAAACGAGAATGACCTTGCATCAAAGTTTTGACCAGACG
82	CAAACCCTCTGAATAATGGAGACAACGTCAG
83	GTGGCATCAATTCACCTCAAATATATATCTTT
84	CCTTATGCGGTGGTTCCGAAATCGGAAAATCCTGTTTGAT
85	ACTATCGATCCTGATAACCGCAATTTGCGTATTGGGCGCCGCCTGGC
86	CTGAAAAGAAAGCGGAATAAATCAGATAGCGTGAGTAACAAGTTTGA
87	AGATACATAACCAAAATAGACAGCTTGATACC
88	GCTGATTGCCCTTCACCAGGGTGGAGATGATGTTATACTT
89	TCCTCATATACCATATCAAAAATCAGTGAGGCCA
90	AACGAGTATGTCTGGAAGTTTCATGGACGTTGACGAGGCA
91	GTCGGGAAACCTGTCGAATAGCCCGAGAAAGGCCACGCTG
92	CCAAAAGGCACAGACAGCCCTCATTCGGCCTCAGGAAGATCGCACTC
93	AGGATTAGACATTATTAATTTTAAAAGTTTGGATTTTAA
94	CTTGAGATGGTTTAATTTCAACTTCGAAAGACTAGTAAGAGCAAGAC
95	CGATGCTGCTCCGGCCAGAGCACATCAGCAGCTGTTTGGAGCAATTCA
96	ATTATCATCATAGTCGGGCAACAGAACTGGCGTGAATTA
97	TTCACCAGTGACACTATCATAACCGGAAGCATCAGAAGC
98	TTACAAACTGCGGAACAAAGGGACGTTTGCCCAGTTGCAGCAAGCGGT
99	AGCATTAACATCCTTTTTATAACATAAAGCTTTTTTAATCGGCGAG
100	ATGACCCAATCACCATCAATATGATTTTTTTTCATTGACCATTAGATACA
101	AAGACTTCAAATTTTTATCGCGTTTTAAAAGAGGA
102	CACTAAAACATTTTTTCATCTTTGGACATTTTTCAACCATCGCCCA
103	TGCAACTAGTCATTTTTGCGGATGGTTTTTTTAGAGCTTAATTGCT
104	GATAGAGAGTTGAGATTTAGTTTTTAATACCACATTCAACGGAACA
105	ATTGTATCTTTTTGTTTATCAGCTTGCTCGGTTGCGCCGACAAT
106	TGCTGTAGCTCATTTTTCATGTTTTAAATAACGAACTA
107	TTTGTATCATCGCCTGATGTACCGTAACAGAGGTGTTTTTATTTCTTAACGA
108	CAGCCAGCAAACTACAACTTTTTCCTGTAGCATTCAGCCTTTA
109	AGGTAGAAAGATCAACGTAACAAAGCTGCTCATTTTTTCAGTGAATAAGGCT
110	ACAAAGTACAATTCCTGAGTTT
111	CGCCACCCTCAGAAGCAGTTGGGTAACGCCAGGGTTTTCCAATAGTGAATT
112	TATCAAAATCATAGGTCTGAGAATTGAGTTACCA
113	TAATTACTAGAAATCAAGAATCCTGAATCTTACCGCCATTTGCAATCAAT
114	ATCTTGACAAGAACCGGATATTCACCAAGCGCGAA
115	GTATAAAGCCAACAGAATAAACACCGGAATCA
116	GTTTAGTAGTTAAATAGCTCAACAAGAATCCTTGAAAACATAGCG
117	ATAAGGCTCAACATCGCCATATGCGTTATACAAATTCTTACCA
118	CCATATTTAACAAGTAATTTACTCCCGATTTCCAGAGCCTAATT
119	AGAGACTATGACTGAAGACGATAACCTTGCTTCTGTAAATCGTC
120	GCTATTAATTAATAACCTCCGTGTGATAACCCTGAACAGCCTTTA
121	ATAGCTTAGATTAGAAGAGTCCAGTCACG
122	TATGTGAGCCTTTTTTTTCCCTTGTAGGGCTTAATTGAGAATCG
123	CTGGTGTGTTCAGCAAATCAGCGGGAGCTAATATCTTCTTTG
124	CCAAGTTACAAAAGAAATTTCTGCTCATTTGCCGCCAGCACATCCCTTACA
125	ATCCCCGGGTACCCACTACTCGAGGTGCCGTAAAGACAATATTGACGCTC
126	GAAATTGTTATCCAGCCTCCTCACAGTTGAGG
127	TTTTTATTTTCATCGTAGGAATCACAGACGACGAC
128	AATAAACAACATCGAAATTAAT
129	GAAGCATAAAGTGGGGTGCCTAAGGGCGCTAAAGGGAGCCCCCG
130	GGTTGCGGTATGATGCCGGGTCGTGCCTGGTACTATGGTGTAGCG
131	GAATTCGTCGTCCGTGGCTCACAAACTGTTGCCCTGCGGCTGGTA
132	ATGGGTAAAGGTTGTCATAAAGTTGGGCG
133	GTTAACGGCGCGCTCTCTTTTTTCGCACTCAATCCGCCGGGCGC
134	ATTGCAGGCATCAGAGCCGGGTCTTCCACACAACATACGAGCCG
135	TTCTTCGAATCCTGTAAAGCATGGTCATAGCTGTTTCCTGTGT
136	TTTGAGGACTAAACCGCTTTTGCGGGATCGTC
137	GAATTGCGAATAATAATTTGGTAATAGTAAATAGTATTATAG
138	GTAGATGGGCGCATCGTAACCGTGAACAACTAAAG
139	ACCCTCAGCAGTAATCATTTCATTATACCAGTCATCCATATAAGAGTACC
140	GCAAAATCCCTTATAAATCAAAAGTGCCAGCTGCA
141	TTAATGAATCGCAGAGCACCGT
142	AACAGTTTCAGCGTAGAAAGGCATCTGCC
143	TTTCACGTTAAAGAAGAGTGAGTTTTGTCGTCTTTCCAGACGTT
144	AGCATCGGGTTAAAGGGACTTTTTGGATTTTGCTAAACAACTTTC
145	CGGGTAAAATACGTACGAAGGAATTGGGAATCTACGTTAATAAA
146	GCAGGGAAACCCACTAATGGAGGGTAGCAACGGCTACAGAGGC
147	TAACGATCTGAAAATTCTGTATGCATGAGGAAGTTTCCATTAAA
148	AGTAAATGAATTTCTCCAAAATGAGGCTTACGATAAAAACGCCAA

Supplementary Table 5 | List of staples in core mix 1 (C1). This staple mixture was used to fold non-passivated triangles capable of self-assembly into shells by shape-complementary base-stacking interactions.

No.	C2 sequences (passivated triangles)
01	GGAAACCACGGTGCGGGCCTCTTCAGCCCAATGTATAAGCAAAAGCCC
02	GCTATTACTAAAATTCGCATTGCTTTAAACAGTT
03	CGCCACCCTCAGAACCGGAATAGCGCAATAATAGGACTGTAGCGCGT
04	AATACCCAACGGAATACAAGATAGCAGCACCG
05	AGTGTACCCCGGTTGCTGCTCCAATTTTCAGGGATAGCA
06	TGTTACTTAGCCGCATTAGACGGAAGACACCAAAGAACT
07	TTAATGCCAGAATCGAGAATATAAACGGTGTACCAACTTTGAAAGAGG
08	GCAGACGGTCAATCATTCCGCGACATAATCAGAAATATTTCTCAGAG
09	AAAATAGCAAAGTCAGAGGAGAAAACTTTTTC
10	AGCCCGAGGAAGATTAGGAACCCATAAATTGTGTCGAAAAAGGGAAC
11	GCCGAACAAAGTTACCAGTAAGACTCAGAGAGATACTGAACA
12	GCCTTTAGCGTCACAAAGGATAAAAATTTTTAGAACCCT
13	TCAAGTTTGGCATGATAAGGAAACAGAGCAAGTTTAGTACACGTTGT
14	AATAAAGCAAATTGTAAACGCGGCCACCCTC
15	TTTTGTCAGGATATCAGGTCATTGCCTGAGAACCCAGCT
16	CCAGTAGCACCGTCACCGACTTGAAACGCTAAATAACATA
17	TGGGTTATATGGAAACAGTACATACCAAGCTTTCAGAGGTGGAGCCG
18	TGTCAATCATATTTAAACGAGGCACAATTTTTTAGTTGC
19	AGAACGCGAGGCGTTTTAGCGAACGGCAGAGG
20	GTCTGGAGAACTAGCAGACAGTCATGTAATACTTTTGAGG
21	CTTGCGGGAGGTTTTGAAGCCTTAAAAAGCCTAAAACAGGGAAGCGG
22	TATTTTGCACCTTCATCAAGAGTAGCGCATAGGCTGGCTG
23	CAAAAATGCATTTTCGTTTGAAATACCGACCGGCTTAGGTATTGAGCG
24	CATATATTAATCAGTAGCGAGGAACCGCCT
25	ACCCCCAGCGATTATATTACCCAAATTCATCAACAGATGA
26	AGCCTTTATTTCAACGCAAATTAAGCTAAAGATT
27	TTTAAATGCAATGCCTGAGTAATGTGTCGGGAGA
28	ATATAAAGTATTTTTCGACAAAAGTTCATTTTTCTTCTGACCTAAA
29	TTAAAGGTGCCAAAGACAAAAGGGCTTTTTACATTCAACCGATTGA
30	AGGTAAATATTGTTTTTCGGAAATTATTCATGCCAGTT
31	TTATTTATCCCCCAATAGCAAGCAAATCAGATTTTTTTAGAAGGCTTATCCG
32	GCTAATGCAGAACGCGCATCAAGAAAACACAAGACTTTTTAAGAACGCGGTA
33	CCACGGGATTTCATTTGATTTTTTTACCTTTTTTAATAACTAT
34	TTTCATCGGCATTTTTTTTCGGACCAGAGCCTTTTTCCACCCAGAA
35	AAATATCAATAAGAAACGATTTTTTTTTTGTTTAACGTACAAAATA
36	AGCCGCCGCAGGTCAGACGATTGGCTTTTTTTGACATTAGCAAGGCCGGA
37	AACGTAGAAAATTTTTACATACATAAAGGCAGTAT
38	ATGTAAATTTTTTCTGATGCAAATCCAATGTTATTTTAGTTAAT
39	GCTCATGGAGCACCAGCAGAAGATAAAACAGAGGTGACAAATCAACA
40	GTAAAGTAATTCTGTCTTACCGCGCAATCCAACATTCCAA
41	TCACCCAAGAGAACAAGCAAGCCGAAGTACCGCACTCATC
42	AATGCGCGAAGAATACGTGGCACAGCACTAAAGCGCGTAA
43	GCCAGCGGGAGGTGTCCAGCATCAATCCCGTAAAAAAAGCCGCACAG
44	TCGGCGAACTCCGTGGTGAAGGGAAAGATGATAACCTATTGTTAATGC
45	TGATTGCTTTGAATAGTACCTTTCCGTTGTAGCCACAGTGCCACGCT
46	CCCTCAGTTTTCATACCCTCAGAAAATAACAGTGCCCGCCTGAACAATTACC
47	TGAGCAAAAGTAGCTCTCACTCCTCAAGAGAAAGTAAGCAGATA
48	AACCACCACCAGGCCACCA
49	AGAAAAATAATCCGCGCTTAATGGGAAAAACATTAGTAA
50	ATTTACGAGCATGTAGAGATAAGTTATAAACAATTCTGAACACCGGA
51	GCTGGCAAGTTGCTTTGACTGCACTCTGTGGT
52	GCTAGGGCAGCTAACTGGTTTCTGCCAGCACGTACCTGCACACGTATA
53	TCAGTATTTAACATCACCATCACGTTAAAGGGGATTCGCCGTTGTGT
54	AAAAACCGTCTATCAGGGCGATGGCCGAGCTCCCACCACACCCGATC
55	TCCTCATTAGGAGTGTGGGAGGGAGAACGGGTGGCTGTCTTTCCTTAT
56	TGGCTTTTAACGGGGTAGGTTGAGACCCTCAGAACCGCCA
57	TAAAGAACGTGGACTCCAACGTCAAATGAGTG
58	CAGTGCCTTGAGTACCCATCCTATTTTGGGGGTGAACCA
59	CTACATTTTTTGTTCCAGTAAGCGTCATACAATCAAGTT
60	ATCAAAATACATGAAAGTATATATCATTTCA
61	AACACCGCCTGCAATCAGCCGCCACCAG
62	GTTAGCATATTTCGGGAAACAAACCTGTTTATCAACAATAAACCAAT
63	CCGAGTAAAAGAGTCTGTCTTGCCTGTCCTCGTTCAGGGCGC
64	GCCTTGCTGGTTTTTTATATCCAGAACAAACTCAA
65	GCGCGGGGAGAGGCGGGAATGCCAACGGCTGTCACTTTTTGCGCGCCTGGAG
66	GCGGCCTTCCCACGCAACTTTTTAGCTTACGGCTGTGCCGGTG
67	CCCCCTGCTTTTTTCAGACGATCCAGCGGCCGGCCAGAATGCGG
68	GCTGCAACAAGGGAAGAAAGTTTTTGAAAGGAGCGGGCCTTGACGG
69	GGAATTGGTCAATAGATAATACATTTTTTTGAGGGCCCTAAAACATCGCC
70	CGCTCACTGCTTTTTCGCTTTCCACGGGTTTTTCGTTTTCACGGTC
71	CGAACGTGGCGAGATAGGGTTGAGTGTTGTTCTTTTTAGTTTGGAACAAGAG
72	GAGAGCCAGCAGCTTTTTAATGTATCTGGTCTTTTTGTTGGGGCGG
73	TCTGACCTATTGGCAGATTCACCAGTTTTTCACACGACCAGTAATA
74	ATTCTGGCCAACTTTTTGAGATAGAACCCTATTTAGAG
75	AACTCCAAAGGTGTTTAGCTATATTTTCATTTGGGGCGTTGTACCAA
76	CAGTTCCTGATTATCTTTTTCTTCGTGGTGCTGGTCTGG
77	CGAGAAACACCAGAACGAGTAGTACACCAACC
78	ACTAATGCTAAAACGACGATATATTCGGTCGCAAAAGGCTGAGGCTTT
79	AGTAACATGGAGCGGATTAGAGCCAGGAAGGTTATCTAAA
80	AATAAAGACGTGGGAATGCCGGACTTGATGTTTAGACTG

81	CAGAAAACGAGAATGACCTTGCATCAAAGTTTTGACCAGACG
82	CAAACCCTCTGAATAATGGAGACAACGTCAG
83	GTGGCATCAATTCACCTCAAATATATATCTTT
84	CCTTATGCGGTGGTTCCGAAATCGGAAAATCCTGTTTGAT
85	ACTATCGATCCTGATAACCGCAATTTGCGTATTGGGCGCCGCCTGGC
86	CTGAAAAGAAAGCGGAATAAATCAGATAGCGTGAGTAACAAGTTTGA
87	AGATACATAACCAAAATAGACAGCTTGATACC
88	GCTGATTGCCCTTCACCAGGGTGGAGATGATGTTATACTT
89	TCCTCATATACCATATCAAAAATCAGTGAGGCCA
90	AACGAGTATGTCTGGAAGTTTCATGGACGTTGACGAGGCA
91	GTCGGGAAACCTGTCGAATAGCCCGAGAAAGGCCACGCTG
92	CCAAAAGGCACAGACAGCCCTCATTCGGCCTCAGGAAGATCGCACTC
93	AGGATTAGACATTATTAATTTTAAAAGTTTGGATTTTAA
94	CTTGAGATGGTTTAATTTCAACTTCGAAAGACTAGTAAGAGCAAGAC
95	CGATGCTGCTCCGGCCAGAGCACATCAGCAGCTGTTTGGAGCAATTCA
96	ATTATCATCATAGTCGGGCAACAGAACTGGCGTGAATTA
97	TTCACCAGTGACACTATCATAACCGGAAGCATCAGAAGC
98	TTACAAACTGCGGAACAAAGGGACGTTTGCCCAGTTGCAGCAAGCGGT
99	AGCATTAACATCCTTTTTATAACATAAAGCTTTTTTAATCGGCGAG
100	ATGACCCAATCACCATCAATATGATTTTTTTTCATTGACCATTAGATACA
101	AAGACTTCAAATTTTTATCGCGTTTTAAAAGAGGA
102	CACTAAAACATTTTTTCATCTTTGGACATTTTTCAACCATCGCCCA
103	TGCAACTAGTCATTTTTGCGGATGGTTTTTTTAGAGCTTAATTGCT
104	GATAGAGAGTTGAGATTTAGTTTTTAATACCACATTCAACGGAACA
105	ATTGTATCTTTTTGTTTATCAGCTTGCTCGGTTGCGCCGACAAT
106	TGCTGTAGCTCATTTTTCATGTTTTAAATAACGAACTA
107	TTTGTATCATCGCCTGATGTACCGTAACAGAGGTGTTTTTATTTCTTAACGA
108	CAGCCAGCAAACTACAACTTTTTCCTGTAGCATTCAGCCTTTA
109	AGGTAGAAAGATCAACGTAACAAAGCTGCTCATTTTTTCAGTGAATAAGGCT
110	ATCTTGACAAGAACCGGATATTCACCAAGCGCGAATTTTT
111	TTTTTTATCAAAATCATAGGTCTGAGAATTGAGTTACCA
112	CGCCACCCTCAGAAGCAGTTGGGTAACGCCAGGGTTTTCCAATAGTGAATTTTTTT
113	TTTTTCGTCACCAGTACTTTCCGGCACCGCTTCTGGTGCCTGCTGCAA
114	TTTTTACAAAGTACAATTCCTGAGTTTTTTTT
115	TTTTTTAATTACTAGAAATCAAGAATCCTGAATCTTACCGCCATTTGCAATCAAT
116	TTTTTCCATATTTAACAAGTAATTTACTCCCGATTTCCAGAGCCTAATT
117	ATAAGGCTCAACATCGCCATATGCGTTATACAAATTCTTACCATTTTT
118	TTTTTGCTATTAATTAATAACCTCCGTGTGATAACCCTGAACAGCCTTTA
119	TATGTGAGCCTTTTTTTTCCCTTGTAGGGCTTAATTGAGAATCGTTTTT
120	TTTTTATAGCTTAGATTAGAAGAGTCCAGTCACG
121	AGAGACTATGACTGAAGACGATAACCTTGCTTCTGTAAATCGTCTTTTT
122	GTTTAGTAGTTAAATAGCTCAACAAGAATCCTTGAAAACATAGCGTTTTT
123	TTTTTCTGGTGTGTTCAGCAAATCAGCGGGAGCTAATATCTTCTTTG
124	TTTTTTACATTTAACAAACGGATAACCTCACCGGAAACAAAGCGGATC
125	TTTTTGAAATTGTTATCCAGCCTCCTCACAGTTGAGGTTTTT
126	CCAAGTTACAAAAGAAATTTCTGCTCATTTGCCGCCAGCACATCCCTTACATTTTT
127	TTTTTAATAAACAACATCGAAATTAATTTTTT
128	TTTTTATCCCCGGGTACCCACTACTCGAGGTGCCGTAAAGACAATATTGACGCTC
129	TTCTTCGAATCCTGTAAAGCATGGTCATAGCTGTTTCCTGTGTTTTTT
130	ATTGCAGGCATCAGAGCCGGGTCTTCCACACAACATACGAGCCGTTTTT
131	TTTTTGGTTGCGGTATGATGCCGGGTCGTGCCTGGTACTATGGTGTAGCG
132	GAATTCGTCGTCCGTGGCTCACAAACTGTTGCCCTGCGGCTGGTATTTTT
133	GTTAACGGCGCGCTCTCTTTTTTCGCACTCAATCCGCCGGGCGCTTTTT
134	TTTTTGAAGCATAAAGTGGGGTGCCTAAGGGCGCTAAAGGGAGCCCCCG
135	TTTTTATGGGTAAAGGTTGTCATAAAGTTGGGCG
136	TTTTTGAATTGCGAATAATAATTTGGTAATAGTAAATAGTATTATAG
137	TTTTTTTAATGAATCGCAGAGCACCGTTTTTT
138	GCAAAATCCCTTATAAATCAAAAGTGCCAGCTGCATTTTT
139	TTTTTACCCTCAGCAGTAATCATTTCATTATACCAGTCATCCATATAAGAGTACC
140	TTTTTTTTGAGGACTAAACCGCTTTTGCGGGATCGTCTTTTT
141	GTAGATGGGCGCATCGTAACCGTGAACAACTAAAGTTTTT
142	GCAGGGAAACCCACTAATGGAGGGTAGCAACGGCTACAGAGGCTTTTT
143	TTTTTAGTAAATGAATTTCTCCAAAATGAGGCTTACGATAAAAACGCCAA
144	TTTTTAACAGTTTCAGCGTAGAAAGGCATCTGCC
145	TTTTTCGGGTAAAATACGTACGAAGGAATTGGGAATCTACGTTAATAAA
146	TAACGATCTGAAAATTCTGTATGCATGAGGAAGTTTCCATTAAATTTTT
147	TTTCACGTTAAAGAAGAGTGAGTTTTGTCGTCTTTCCAGACGTTTTTTT
148	AGCATCGGGTTAAAGGACTTTTTGGATTTTGCTAAACAACTTTCTTTTT

Supplementary Table 6 | List of staples in core mix 2 (C2). This staple mixture was used to fold passivated triangles incapable of self-assembly into shells by shape-complementary base-stacking interactions due to protruding dT nucleotides at the stacking contacts.

No.	I1 sequences (internal mix 1)
01	TGATATAAGTATAGCCAACCAATACAAAGAATTAATTAATATTTTGT
02	CCATGTTTACATAGCTATCTTACAGGAAACAATG
03	AAAACGACGGCCAGTGAATCAATAAAATAGCACTAATATC
04	GGCGATTATGGCGAAAGGGGGATGAATAAATTTTTGTTAAATCAAAA
05	ATCATACAGGCAAGGGGAACGCCATCAGCTCATTTTTT
06	GAGAGGGTCCGTACTCAGGCGAAGCCCTTTTTTTTTAA
07	CAGTCCCGGAATTTGTTTTTGAGAGATAGACTTTACGTACAGCG
08	AAACTTAAAAAGAGACGCAGAAACGAAAGGATTAGGATTATGCCGTC
09	ACATCGACATAAAAAAGCGGGGTCCAGGAACGACGTGCTT
10	CGGCAAACGCGTACGCCAGAATCACGATTTTAGA
11	TCATAGCCCCCTTATCGGATAAGGCGGGGTTTTGCTCA
12	TGAATATACAGTAACAGTACCAGGTAGCGTTTGCATAAGAGGCTGAG
13	GGTCCGTTTTTTCGTTTTTTTCGTCGCTGGCAGCATTGCCGTTC
14	ACGTCAGAGAGAAACAATACTGAGAAGTGTTTTTTTTT
15	CACCTTGCCCTAGGGTTAGAACCACGGAACGCAAACGGC
16	CATTCGCCATGTCATAAATATTCAGCCCAATACT
17	CAATCAAAAAAATCTAAAGCATAGATTTTCAAACAGA
18	AAAATCAGTTCATCAACGTCTGGCCTTCCTGTAATTGCGT
19	ACGTTGGTGGATTGACCGTAATGGATATTATTTGCACGTAAGGTTTA
20	GGGGACGACGACAGTAAGTTAGCGGCGGAATCTGCAAAAG
21	TCAGGCTGCGCAACTTTTTTTTGGGAAGGGCGATGGCAAAGCGC
22	ATAATTCGCATTAAATGTGATTGAATCCCTTTTTCTCA
23	CGTCACCAGTACTTTCCGGCACCGCTTCTGGTGCCTGCTGCAA
24	TACATTTAACAAACGGATAACCTCACCGGAAACAAAGCGGATC
25	CGGTGGTGCCATTAGTGATGAAGGGTAAAGTTAAAGATAGGTC

Supplementary Table 7 | List of staples in internal linker mix 1 (I1).

No.	I2 sequences (internal mix 2)
01	TGATATAAGTATAGCCAACCAATACAAAGAATTAATTAATATTTTGT
02	CCATGTTTACATAGCTATCTTACAGGAAACAATG
03	GCCACCGCCACCCTCAGAGAGCCCAATAATACGAGGAAAGTGTATCA
04	AAAACGACGGCCAGTGAATCAATAAAATAGCACTAATATC
05	GGCGATTATGGCGAAAGGGGGATGAATAAATTTTTGTTAAATCAAAA
06	ATCATACAGGCAAGGGGAACGCCATCAGCTCATTTTTT
07	CAGTCCCGGAATTTGTTTTTGAGAGATAGACTTTACGTACAGCG
08	AAACTTAAAAAGAGACGCAGAAACGAAAGGATTAGGATTATGCCGTC
09	ACATCGACATAAAAAAGCGGGGTCCAGGAACGACGTGCTT
10	CGGCAAACGCGTACGCCAGAATCACGATTTTAGA
11	ACGTCGCGCAGAGGCGAATACAGGAGGCCGACAAATTAATACATCGG
12	TCATAGCCCCCTTATCGGATAAGGCGGGGTTTTGCTCA
13	TGAATATACAGTAACAGTACCAGGTAGCGTTTGCATAAGAGGCTGAG
14	GGTCCGTTTTTTCGTTTTTTTCGTCGCTGGCAGCATTGCCGTTC
15	CACCTTGCCCTAGGGTTAGAACCACGGAACGCAAACGGC
16	CATTCGCCATGTCATAAATATTCAGCCCAATACT
17	ACCCGTCGGATTCTCAGCCAGCTGTCTTTACTGATACTAATAGTAGT
18	CAATCAAAAAAATCTAAAGCATAGATTTTCAAACAGA
19	AAAATCAGTTCATCAACGTCTGGCCTTCCTGTAATTGCGT
20	ACGTTGGTGGATTGACCGTAATGGATATTATTTGCACGTAAGGTTTA
21	GGGGACGACGACAGTAAGTTAGCGGCGGAATCTGCAAAAG
22	TCAGGCTGCGCAACTTTTTTTTGGGAAGGGCGATGGCAAAGCGC
23	CGTCACCAGTACTTTCCGGCACCGCTTCTGGTGCCTGCTGCAA
24	TACATTTAACAAACGGATAACCTCACCGGAAACAAAGCGGATC
25	CGGTGGTGCCATTAGTGATGAAGGGTAAAGTTAAAGATAGGTC
26	GAGAGGGTCCGTACTCAGGCGAAGCCCTTTTTTTTTAATTTTTTTTTTTTTTTTTTTT**AAAAGGTGAGATGAT**
27	ACGTCAGAGAGAAACAATACTGAGAAGTGTTTTTTTTTTTTTTTTTTTTTTTTTTTTT**AAAAGGTGAGATGAT**
28	ATAATTCGCATTAAATGTGATTGAATCCCTTTTTCTCATTTTTTTTTTTTTTTTTTTT**AAAAGGTGAGATGAT**

Supplementary Table 8 | List of staples in internal linker mix 2 (I2). Highlighted sequences are complementary to functionalised oligonucleotides (Supplementary Table 3).

No.	I3 sequences (internal mix 3)
01	TGATATAAGTATAGCCAACCAATACAAAGAATTAATTAATATTTTGT
02	CCATGTTTACATAGCTATCTTACAGGAAACAATG
03	GCCACCGCCACCCTCAGAGAGCCCAATAATACGAGGAAAGTGTATCA
04	AAAACGACGGCCAGTGAATCAATAAAATAGCACTAATATC
05	GGCGATTATGGCGAAAGGGGGATGAATAAATTTTTGTTAAATCAAAA
06	ATCATACAGGCAAGGGGAACGCCATCAGCTCATTTTTT
07	CAGTCCCGGAATTTGTTTTTGAGAGATAGACTTTACGTACAGCG
08	AAACTTAAAAAGAGACGCAGAAACGAAAGGATTAGGATTATGCCGTC
09	ACATCGACATAAAAAAGCGGGGTCCAGGAACGACGTGCTT
10	CGGCAAACGCGTACGCCAGAATCACGATTTTAGA
11	ACGTCGCGCAGAGGCGAATACAGGAGGCCGACAAATTAATACATCGG
12	TCATAGCCCCCTTATCGGATAAGGCGGGGTTTTGCTCA
13	TGAATATACAGTAACAGTACCAGGTAGCGTTTGCATAAGAGGCTGAG
14	GGTCCGTTTTTTCGTTTTTTTCGTCGCTGGCAGCATTGCCGTTC
15	CACCTTGCCCTAGGGTTAGAACCACGGAACGCAAACGGC
16	CATTCGCCATGTCATAAATATTCAGCCCAATACT
17	ACCCGTCGGATTCTCAGCCAGCTGTCTTTACTGATACTAATAGTAGT
18	CAATCAAAAAAATCTAAAGCATAGATTTTCAAACAGA
19	AAAATCAGTTCATCAACGTCTGGCCTTCCTGTAATTGCGT
20	ACGTTGGTGGATTGACCGTAATGGATATTATTTGCACGTAAGGTTTA
21	GGGGACGACGACAGTAAGTTAGCGGCGGAATCTGCAAAAG
22	TCAGGCTGCGCAACTTTTTTTTGGGAAGGGCGATGGCAAAGCGC
23	CGTCACCAGTACTTTCCGGCACCGCTTCTGGTGCCTGCTGCAA
24	TACATTTAACAAACGGATAACCTCACCGGAAACAAAGCGGATC
25	CGGTGGTGCCATTAGTGATGAAGGGTAAAGTTAAAGATAGGTC
26	GAGAGGGTCCGTACTCAGGCGAAGCCCTTTTTTTTTAATTTTTTTTTTTTTTTTTTTT**GCTGCTTCCGGCGTA**
27	ACGTCAGAGAGAAACAATACTGAGAAGTGTTTTTTTTTTTTTTTTTTTTTTTTTTTTT**GCTGCTTCCGGCGTA**
28	ATAATTCGCATTAAATGTGATTGAATCCCTTTTTCTCATTTTTTTTTTTTTTTTTTTT**GCTGCTTCCGGCGTA**

Supplementary Table 9 | List of staples in internal linker mix 3 (I3). Highlighted sequences are complementary to functionalised oligonucleotides (Supplementary Table 3).

No.	I4 sequences (internal mix 4)
01	TGATATAAGTATAGCCAACCAATACAAAGAATTAATTAATATTTTGT
02	CCATGTTTACATAGCTATCTTACAGGAAACAATG
03	GCCACCGCCACCCTCAGAGAGCCCAATAATACGAGGAAAGTGTATCA
04	AAAACGACGGCCAGTGAATCAATAAAATAGCACTAATATC
05	GGCGATTATGGCGAAAGGGGGATGAATAAATTTTTGTTAAATCAAAA
06	ATCATACAGGCAAGGGGAACGCCATCAGCTCATTTTTT
07	GAGAGGGTCCGTACTCAGGCGAAGCCCTTTTTTTTTAA
08	CAGTCCCGGAATTTGTTTTTGAGAGATAGACTTTACGTACAGCG
09	AAACTTAAAAAGAGACGCAGAAACGAAAGGATTAGGATTATGCCGTC
10	ACATCGACATAAAAAAGCGGGGTCCAGGAACGACGTGCTT
11	CGGCAAACGCGTACGCCAGAATCACGATTTTAGA
12	ACGTCGCGCAGAGGCGAATACAGGAGGCCGACAAATTAATACATCGG
13	TCATAGCCCCCTTATCGGATAAGGCGGGGTTTTGCTCA
14	TGAATATACAGTAACAGTACCAGGTAGCGTTTGCATAAGAGGCTGAG
15	GGTCCGTTTTTTCGTTTTTTTCGTCGCTGGCAGCATTGCCGTTC
16	ACGTCAGAGAGAAACAATACTGAGAAGTGTTTTTTTTT
17	CACCTTGCCCTAGGGTTAGAACCACGGAACGCAAACGGC
18	CATTCGCCATGTCATAAATATTCAGCCCAATACT
19	ACCCGTCGGATTCTCAGCCAGCTGTCTTTACTGATACTAATAGTAGT
20	CAATCAAAAAAATCTAAAGCATAGATTTTCAAACAGA
21	AAAATCAGTTCATCAACGTCTGGCCTTCCTGTAATTGCGT
22	ACGTTGGTGGATTGACCGTAATGGATATTATTTGCACGTAAGGTTTA
23	GGGGACGACGACAGTAAGTTAGCGGCGGAATCTGCAAAAG
24	TCAGGCTGCGCAACTTTTTTTTGGGAAGGGCGATGGCAAAGCGC
25	ATAATTCGCATTAAATGTGATTGAATCCCTTTTTCTCA
26	CGTCACCAGTACTTTCCGGCACCGCTTCTGGTGCCTGCTGCAA
27	TACATTTAACAAACGGATAACCTCACCGGAAACAAAGCGGATC
28	CGGTGGTGCCATTAGTGATGAAGGGTAAAGTTAAAGATAGGTC

Supplementary Table 10 | List of staples in internal linker mix 4 (I4).

No.	I5 sequences (internal mix 5)
01	GCCACCGCCACCCTCAGAGAGCCCAATAATACGAGGAAAGTGTATCA
02	ATCATACAGGCAAGGGGAACGCCATCAGCTCATTTTTT
03	GAGAGGGTCCGTACTCAGGCGAAGCCCTTTTTTTTTAA
04	ACGTCGCGCAGAGGCGAATACAGGAGGCCGACAAATTAATACATCGG
05	TCATAGCCCCCTTATCGGATAAGGCGGGGTTTTGCTCA
06	ACGTCAGAGAGAAACAATACTGAGAAGTGTTTTTTTTT
07	ACCCGTCGGATTCTCAGCCAGCTGTCTTTACTGATACTAATAGTAGT
08	CAATCAAAAAAATCTAAAGCATAGATTTTCAAACAGA
09	ATAATTCGCATTAAATGTGATTGAATCCCTTTTTCTCA
10	TGATATAAGTATAGCCAACCAATACAAAGAATTAATTAATATTTTGT
11	TGAATATACAGTAACAGTACCAGGTAGCGTTTGCATAAGAGGCTGAG
12	CACCTTGCCCTAGGGTTAGAACCACGGAACGCAAACGGC
13	AAAATCAGTTCATCAACGTCTGGCCTTCCTGTAATTGCGT
14	CAGTCCCGGAATTTGTTTTTGAGAGATAGACTTTACGTACAGCGCCATGTTTAC
15	GGTCCGTTTTTTCGTTTTTTTCGTCGCTGGCAGCATTGCCGTTCCGGCAAACGC
16	TCAGGCTGCGCAACTTTTTTTTGGGAAGGGCGATGGCAAAGCGCCATTCGCCAT
17	GGGGGATGAATAAATTTTTGTTAAATCAAAA
18	CGTCACCAGTACTTTCCGGCACCGCTT
19	GCAGAAACGAAAGGATTAGGATTATGCCGTC
20	TACATTTAACAAACGGATAACCTCACC
21	CGTAATGGATATTATTTGCACGTAAGGTTTA
22	CGGTGGTGCCATTAGTGATGAAGGGTA
23	**AAAAGGTGAGATGAT**TTTAAAACGACGGCCAGTGAATCAATAAAATAGCACTAATATC
24	**AAAAGGTGAGATGAT**TTTACATCGACATAAAAAAGCGGGGTCCAGGAACGACGTGCTT
25	**AAAAGGTGAGATGAT**TTTGGGGACGACGACAGTAAGTTAGCGGCGGAATCTGCAAAAG
26	**AAAAGGTGAGATGAT**TTTATAGCTATCTTACAGGAAACAATG
27	**AAAAGGTGAGATGAT**TTTGTACGCCAGAATCACGATTTTAGA
28	**AAAAGGTGAGATGAT**TTTGTCATAAATATTCAGCCCAATACT
29	**AAAAGGTGAGATGAT**TTTCTGGTGCCTGCTGCAAGGCGATTATGGCGAAA
30	**AAAAGGTGAGATGAT**TTTGGAAACAAAGCGGATCAAACTTAAAAAGAGAC
31	**AAAAGGTGAGATGAT**TTTAAGTTAAAGATAGGTCACGTTGGTGGATTGAC

Supplementary Table 11 | List of staples in internal linker mix 5 (I5). Highlighted sequences are complementary to functionalised oligonucleotides (Supplementary Table 3).

No.	I6 sequences (internal mix 6)
01	CCATGTTTACATAGCTATCTTACAGGAAACAATG
02	AAAACGACGGCCAGTGAATCAATAAAATAGCACTAATATC
03	GGCGATTATGGCGAAAGGGGGATGAATAAATTTTTGTTAAATCAAAA
04	CAGTCCCGGAATTTGTTTTTGAGAGATAGACTTTACGTACAGCG
05	AAACTTAAAAAGAGACGCAGAAACGAAAGGATTAGGATTATGCCGTC
06	ACATCGACATAAAAAAGCGGGGTCCAGGAACGACGTGCTT
07	CGGCAAACGCGTACGCCAGAATCACGATTTTAGA
08	GGTCCGTTTTTTCGTTTTTTTCGTCGCTGGCAGCATTGCCGTTC
09	CATTCGCCATGTCATAAATATTCAGCCCAATACT
10	ACGTTGGTGGATTGACCGTAATGGATATTATTTGCACGTAAGGTTTA
11	GGGGACGACGACAGTAAGTTAGCGGCGGAATCTGCAAAAG
12	TCAGGCTGCGCAACTTTTTTTTGGGAAGGGCGATGGCAAAGCGC
13	CGTCACCAGTACTTTCCGGCACCGCTTCTGGTGCCTGCTGCAA
14	TGATATAAGTATAGCCAACCAATACAAAGAATTAATTAATATTTTGT
15	TGAATATACAGTAACAGTACCAGGTAGCGTTTGCATAAGAGGCTGAG
16	CACCTTGCCCTAGGGTTAGAACCACGGAACGCAAACGGC
17	AAAATCAGTTCATCAACGTCTGGCCTTCCTGTAATTGCGT
18	TACATTTAACAAACGGATAACCTCACCGGAAACAAAGCGGATC
19	CGGTGGTGCCATTAGTGATGAAGGGTAAAGTTAAAGATAGGTC
20	GTCTTTACTGATACTAATAGTAGT
21	GCCACCGCCACCCTCAGAGAGCCCAATAATACGAGGAAAGTGTATCA**TAGTAGAGTGGAAAA**
22	ATCATACAGGCAAGGGGAACGCCATCAGCTCATTTTTT**TAGTAGAGTGGAAAA**
23	ACGTCGCGCAGAGGCGAATACAGGAGGCCGACAAATTAATACATCGG**TAGTAGAGTGGAAAA**
24	TCATAGCCCCCTTATCGGATAAGGCGGGGTTTTGCTCA**TAGTAGAGTGGAAAA**
25	ACCCGTCGGATTCTCAGCCAGCT**TAGTAGAGTGGAAAA**
26	CAATCAAAAAAATCTAAAGCATAGATTTTCAAACAGA**TAGTAGAGTGGAAAA**
27	GAGAGGGTCCGTACTCAGGCGAAGCCCTTTTTTTTTAA**TAGTAGAGTGGAAAA**
28	ACGTCAGAGAGAAACAATACTGAGAAGTGTTTTTTTTT**TAGTAGAGTGGAAAA**
29	ATAATTCGCATTAAATGTGATTGAATCCCTTTTTCTCA**TAGTAGAGTGGAAAA**

Supplementary Table 12 | List of staples in internal linker mix 6 (I6). Highlighted sequences are complementary to functionalised oligonucleotides (Supplementary Table 3).

No.	I7 sequences (internal mix 7)
01	CCATGTTTACATAGCTATCTTACAGGAAACAATG
02	AAAACGACGGCCAGTGAATCAATAAAATAGCACTAATATC
03	GGCGATTATGGCGAAAGGGGGATGAATAAATTTTTGTTAAATCAAAA
04	ATCATACAGGCAAGGGGAACGCCATCAGCTCATTTTTT
05	GAGAGGGTCCGTACTCAGGCGAAGCCCTTTTTTTTTAA
06	CAGTCCCGGAATTTGTTTTTGAGAGATAGACTTTACGTACAGCG
07	AAACTTAAAAAGAGACGCAGAAACGAAAGGATTAGGATTATGCCGTC
08	ACATCGACATAAAAAAGCGGGGTCCAGGAACGACGTGCTT
09	CGGCAAACGCGTACGCCAGAATCACGATTTTAGA
10	TCATAGCCCCCTTATCGGATAAGGCGGGGTTTTGCTCA
11	GGTCCGTTTTTTCGTTTTTTTCGTCGCTGGCAGCATTGCCGTTC
12	ACGTCAGAGAGAAACAATACTGAGAAGTGTTTTTTTTT
13	CATTCGCCATGTCATAAATATTCAGCCCAATACT
14	CAATCAAAAAAATCTAAAGCATAGATTTTCAAACAGA
15	ACGTTGGTGGATTGACCGTAATGGATATTATTTGCACGTAAGGTTTA
16	GGGGACGACGACAGTAAGTTAGCGGCGGAATCTGCAAAAG
17	TCAGGCTGCGCAACTTTTTTTTGGGAAGGGCGATGGCAAAGCGC
18	ATAATTCGCATTAAATGTGATTGAATCCCTTTTTCTCA
19	CGTCACCAGTACTTTCCGGCACCGCTTCTGGTGCCTGCTGCAA
20	TGATATAAGTATAGCCAACCAATACAAAGAATTAATTAATATTTTGT
21	TGAATATACAGTAACAGTACCAGGTAGCGTTTGCATAAGAGGCTGAG
22	CACCTTGCCCTAGGGTTAGAACCACGGAACGCAAACGGC
23	AAAATCAGTTCATCAACGTCTGGCCTTCCTGTAATTGCGT
24	TACATTTAACAAACGGATAACCTCACCGGAAACAAAGCGGATC
25	CGGTGGTGCCATTAGTGATGAAGGGTAAAGTTAAAGATAGGTC
26	GTCTTTACTGATACTAATAGTAGT
27	GCCACCGCCACCCTCAGAGAGCCCAATAATACGAGGAAAGTGTATCATTTTTTTTTTTTTTTTTT**AAAAGGTGAGATGAT**
28	ACGTCGCGCAGAGGCGAATACAGGAGGCCGACAAATTAATACATCGGTTTTTTTTTTTTTTTTTT**AAAAGGTGAGATGAT**
29	ACCCGTCGGATTCTCAGCCAGCTTTTTTTTTTTTTTTTTTT**AAAAGGTGAGATGAT**

Supplementary Table 13 | List of staples in internal linker mix 7 (I7). Highlighted sequences are complementary to functionalised oligonucleotides (Supplementary Table 3).

No.	I8 sequences (internal mix 8)
01	TGATATAAGTATAGCCAACCAATACAAAGAATTAATTAATATTTTGT
02	TGAATATACAGTAACAGTACCAGGTAGCGTTTGCATAAGAGGCTGAG
03	CACCTTGCCCTAGGGTTAGAACCACGGAACGCAAACGGC
04	AAAATCAGTTCATCAACGTCTGGCCTTCCTGTAATTGCGT
05	CAGTCCCGGAATTTGTTTTTGAGAGATAGACTTTACGTACAGCGCCATGTTTAC
06	GGTCCGTTTTTTCGTTTTTTTCGTCGCTGGCAGCATTGCCGTTCCGGCAAACGC
07	TCAGGCTGCGCAACTTTTTTTTGGGAAGGGCGATGGCAAAGCGCCATTCGCCAT
08	GGGGGATGAATAAATTTTTGTTAAATCAAAA
09	CGTCACCAGTACTTTCCGGCACCGCTT
10	GCAGAAACGAAAGGATTAGGATTATGCCGTC
11	TACATTTAACAAACGGATAACCTCACC
12	CGTAATGGATATTATTTGCACGTAAGGTTTA
13	CGGTGGTGCCATTAGTGATGAAGGGTA
14	GTCTTTACTGATACTAATAGTAGT
15	GCCACCGCCACCCTCAGAGAGCCCAATAATACGAGGAAAGTGTATCATTTTTTTTTTTTTTTTTT**AAAAGGTGAGATGAT**
16	ATCATACAGGCAAGGGGAACGCCATCAGCTCATTTTTTTTTTTTTTTTTTTTTTTT**AAAAGGTGAGATGAT**
17	ACGTCGCGCAGAGGCGAATACAGGAGGCCGACAAATTAATACATCGGTTTTTTTTTTTTTTTTTT**AAAAGGTGAGATGAT**
18	TCATAGCCCCCTTATCGGATAAGGCGGGGTTTGCTCATTTTTTTTTTTTTTTTTTT**AAAAGGTGAGATGAT**
19	ACCCGTCGGATTCTCAGCCAGCTTTTTTTTTTTTTTTTTTT**AAAAGGTGAGATGAT**
20	CAATCAAAAAAATCTAAAGCATAGATTTCAAACAGATTTTTTTTTTTTTTTTTTT**AAAAGGTGAGATGAT**
21	GAGAGGGTCCGTACTCAGGCGAAGCCCTTTTTTTTAATTTTTTTTTTTTTTTTTTT**AAAAGGTGAGATGAT**
22	ACGTCAGAGAGAAACAATACTGAGAAGTGTTTTTTTTTTTTTTTTTTTTTTTTTTT**AAAAGGTGAGATGAT**
23	ATAATTCGCATTAAATGTGATTGAATCCCTTTTTCTCATTTTTTTTTTTTTTTTTT**AAAAGGTGAGATGAT**
24	**AAAAGGTGAGATGAT**TTTAAAACGACGGCCAGTGAATCAATAAAATAGCACTAATATC
25	**AAAAGGTGAGATGAT**TTTACATCGACATAAAAAAGCGGGGTCCAGGAACGACGTGCTT
26	**AAAAGGTGAGATGAT**TTTGGGGACGACGACAGTAAGTTAGCGGCGGAATCTGCAAAAG
27	**AAAAGGTGAGATGAT**TTTATAGCTATCTTACAGGAAACAATG
28	**AAAAGGTGAGATGAT**TTTGTACGCCAGAATCACGATTTTAGA
29	**AAAAGGTGAGATGAT**TTTGTCATAAATATTCAGCCCAATACT
30	**AAAAGGTGAGATGAT**TTTCTGGTGCCTGCTGCAAGGCGATTATGGCGAAA
31	**AAAAGGTGAGATGAT**TTTGGAAACAAAGCGGATCAAACTTAAAAAGAGAC
32	**AAAAGGTGAGATGAT**TTTAAGTTAAAGATAGGTCACGTTGGTGGATTGAC

Supplementary Table 14 | List of staples in internal linker mix 8 (I8). Highlighted sequences are complementary to functionalised oligonucleotides (Supplementary Table 3).

No.	I9 sequences (internal mix 9)
01	CCATGTTTACATAGCTATCTTACAGGAAACAATG
02	AAAACGACGGCCAGTGAATCAATAAAATAGCACTAATATC
03	GGCGATTATGGCGAAAGGGGGATGAATAAATTTTTGTTAAATCAAAA
04	ATCATACAGGCAAGGGGAACGCCATCAGCTCATTTTTT
05	GAGAGGGTCCGTACTCAGGCGAAGCCCTTTTTTTTTAA
06	CAGTCCCGGAATTTGTTTTTGAGAGATAGACTTTACGTACAGCG
07	AAACTTAAAAAGAGACGCAGAAACGAAAGGATTAGGATTATGCCGTC
08	ACATCGACATAAAAAAGCGGGGTCCAGGAACGACGTGCTT
09	CGGCAAACGCGTACGCCAGAATCACGATTTTAGA
10	ACGTCGCGCAGAGGCGAATACAGGAGGCCGACAAATTAATACATCGG
11	TCATAGCCCCCTTATCGGATAAGGCGGGGTTTTGCTCA
12	GGTCCGTTTTTTCGTTTTTTTCGTCGCTGGCAGCATTGCCGTTC
13	ACGTCAGAGAGAAACAATACTGAGAAGTGTTTTTTTTT
14	CATTCGCCATGTCATAAATATTCAGCCCAATACT
15	ACCCGTCGGATTCTCAGCCAGCTGTCTTTACTGATACTAATAGTAGT
16	CAATCAAAAAAATCTAAAGCATAGATTTTCAAACAGA
17	ACGTTGGTGGATTGACCGTAATGGATATTATTTGCACGTAAGGTTTA
18	GGGGACGACGACAGTAAGTTAGCGGCGGAATCTGCAAAAG
19	TCAGGCTGCGCAACTTTTTTTTGGGAAGGGCGATGGCAAAGCGC
20	ATAATTCGCATTAAATGTGATTGAATCCCTTTTTCTCA
21	CGTCACCAGTACTTTCCGGCACCGCTTCTGGTGCCTGCTGCAA
22	TGATATAAGTATAGCCAACCAATACAAAGAATTAATTAATATTTTGT
23	TGAATATACAGTAACAGTACCAGGTAGCGTTTGCATAAGAGGCTGAG
24	CACCTTGCCCTAGGGTTAGAACCACGGAACGCAAACGGC
25	AAAATCAGTTCATCAACGTCTGGCCTTCCTGTAATTGCGT
26	TACATTTAACAAACGGATAACCTCACCGGAAACAAAGCGGATC
27	CGGTGGTGCCATTAGTGATGAAGGGTAAAGTTAAAGATAGGTC
28	GCCACCGCCACCCTCAGAGAGCCCAATAATACGAGGAAAGTGTATCATTTTTTTTTTTTTTTTTT**AAAAGGTGAGATGAT**

Supplementary Table 15 | List of staples in internal linker mix 9 (I9). Highlighted sequences are complementary to functionalised oligonucleotides (Supplementary Table 3).

No.	I10 sequences (internal mix 10)
01	TGATATAAGTATAGCCAACCAATACAAAGAATTAATTAATATTTTGT
02	CCATGTTTACATAGCTATCTTACAGGAAACAATG
03	GCCACCGCCACCCTCAGAGAGCCCAATAATACGAGGAAAGTGTATCA
04	AAAACGACGGCCAGTGAATCAATAAAATAGCACTAATATC
05	GGCGATTATGGCGAAAGGGGGATGAATAAATTTTTGTTAAATCAAAA
06	ATCATACAGGCAAGGGGAACGCCATCAGCTCATTTTTT
07	CAGTCCCGGAATTTGTTTTTGAGAGATAGACTTTACGTACAGCG
08	AAACTTAAAAAGAGACGCAGAAACGAAAGGATTAGGATTATGCCGTC
09	ACATCGACATAAAAAAGCGGGGTCCAGGAACGACGTGCTT
10	CGGCAAACGCGTACGCCAGAATCACGATTTTAGA
11	ACGTCGCGCAGAGGCGAATACAGGAGGCCGACAAATTAATACATCGG
12	TCATAGCCCCCTTATCGGATAAGGCGGGGTTTTGCTCA
13	TGAATATACAGTAACAGTACCAGGTAGCGTTTGCATAAGAGGCTGAG
14	GGTCCGTTTTTTCGTTTTTTTCGTCGCTGGCAGCATTGCCGTTC
15	CACCTTGCCCTAGGGTTAGAACCACGGAACGCAAACGGC
16	CATTCGCCATGTCATAAATATTCAGCCCAATACT
17	ACCCGTCGGATTCTCAGCCAGCTGTCTTTACTGATACTAATAGTAGT
18	CAATCAAAAAAATCTAAAGCATAGATTTTCAAACAGA
19	AAAATCAGTTCATCAACGTCTGGCCTTCCTGTAATTGCGT
20	ACGTTGGTGGATTGACCGTAATGGATATTATTTGCACGTAAGGTTTA
21	GGGGACGACGACAGTAAGTTAGCGGCGGAATCTGCAAAAG
22	TCAGGCTGCGCAACTTTTTTTTGGGAAGGGCGATGGCAAAGCGC
23	CGTCACCAGTACTTTCCGGCACCGCTTCTGGTGCCTGCTGCAA
24	TACATTTAACAAACGGATAACCTCACCGGAAACAAAGCGGATC
25	CGGTGGTGCCATTAGTGATGAAGGGTAAAGTTAAAGATAGGTC
26	GAGAGGGTCCGTACTCAGGCGAAGCCCTTTTTTTTTAATTTTTTTTTTTTTT**AAAAGGTGAGATGAT**
27	ACGTCAGAGAGAAACAATACTGAGAAGTGTTTTTTTTTTTTTTTTTTTTTTT**AAAAGGTGAGATGAT**
28	ATAATTCGCATTAAATGTGATTGAATCCCTTTTTCTCATTTTTTTTTTTTTT**AAAAGGTGAGATGAT**

Supplementary Table 16 | List of staples in internal linker mix 10 (I10). Highlighted sequences are complementary to functionalised oligonucleotides (Supplementary Table 3).

No.	I11 sequences (internal mix 11)
01	TGATATAAGTATAGCCAACCAATACAAAGAATTAATTAATATTTTGT
02	CCATGTTTACATAGCTATCTTACAGGAAACAATG
03	AAAACGACGGCCAGTGAATCAATAAAATAGCACTAATATC
04	GGCGATTATGGCGAAAGGGGGATGAATAAATTTTTGTTAAATCAAAA
05	GAGAGGGTCCGTACTCAGGCGAAGCCCTTTTTTTTTAA
06	CAGTCCCGGAATTTGTTTTTGAGAGATAGACTTTACGTACAGCG
07	AAACTTAAAAAGAGACGCAGAAACGAAAGGATTAGGATTATGCCGTC
08	ACATCGACATAAAAAAGCGGGGTCCAGGAACGACGTGCTT
09	CGGCAAACGCGTACGCCAGAATCACGATTTTAGA
10	TGAATATACAGTAACAGTACCAGGTAGCGTTTGCATAAGAGGCTGAG
11	GGTCCGTTTTTTCGTTTTTTTTCGTCGCTGGCAGCATTGCCGTTC
12	ACGTCAGAGAGAAACAATACTGAGAAGTGTTTTTTTTT
13	CACCTTGCCCTAGGGTTAGAACCACGGAACGCAAACGGC
14	CATTCGCCATGTCATAAATATTCAGCCCAATACT
15	AAAATCAGTTCATCAACGTCTGGCCTTCCTGTAATTGCGT
16	ACGTTGGTGGATTGACCGTAATGGATATTATTTGCACGTAAGGTTTA
17	GGGGACGACGACAGTAAGTTAGCGGCGGAATCTGCAAAAG
18	TCAGGCTGCGCAACTTTTTTTTGGGAAGGGCGATGGCAAAGCGC
19	ATAATTCGCATTAAATGTGATTGAATCCCTTTTTCTCA
20	CGTCACCAGTACTTTCCGGCACCGCTTCTGGTGCCTGCTGCAA
21	TACATTTAACAAACGGATAACCTCACCGGAAACAAAGCGGATC
22	CGGTGGTGCCATTAGTGATGAAGGGTAAAGTTAAAGATAGGTC
23	GTCTTTACTGATACTAATAGTAGT
24	GCCACCGCCACCCTCAGAGAGCCCAATAATACGAGGAAAGTGTATCATTTTTTTTTTTTTT**AAAAGGTGAGATGAT**
25	ATCATACAGGCAAGGGGAACGCCATCAGCTCATTTTTTTTTTTTTTTTTTTT**AAAAGGTGAGATGAT**
26	ACGTCGCGCAGAGGCGAATACAGGAGGCCGACAAATTAATACATCGGTTTTTTTTTTTTTT**AAAAGGTGAGATGAT**
27	TCATAGCCCCCTTATCGGATAAGGCGGGGTTTGCTCATTTTTTTTTTTTTTT**AAAAGGTGAGATGAT**
28	ACCCGTCGGATTCTCAGCCAGCTTTTTTTTTTTTTTT**AAAAGGTGAGATGAT**
29	CAATCAAAAAAATCTAAAGCATAGATTTTCAAACAGATTTTTTTTTTTTTT**AAAAGGTGAGATGAT**

Supplementary Table 17 | List of staples in internal linker mix 11 (I11). Highlighted sequences are complementary to functionalised oligonucleotides (Supplementary Table 3).

No.	I12 sequences (internal mix 12)
01	TGATATAAGTATAGCCAACCAATACAAAGAATTAATTAATATTTTGT
02	CCATGTTTACATAGCTATCTTACAGGAAACAATG
03	AAAACGACGGCCAGTGAATCAATAAAATAGCACTAATATC
04	GGCGATTATGGCGAAAGGGGGATGAATAAATTTTTGTTAAATCAAAA
05	CAGTCCCGGAATTTGTTTTTGAGAGATAGACTTTACGTACAGCG
06	AAACTTAAAAAGAGACGCAGAAACGAAAGGATTAGGATTATGCCGTC
07	ACATCGACATAAAAAAGCGGGGTCCAGGAACGACGTGCTT
08	CGGCAAACGCGTACGCCAGAATCACGATTTTAGA
09	TGAATATACAGTAACAGTACCAGGTAGCGTTTGCATAAGAGGCTGAG
10	GGTCCGTTTTTTCGTTTTTTTCGTCGCTGGCAGCATTGCCGTTC
11	CACCTTGCCCTAGGGTTAGAACCACGGAACGCAAACGGC
12	CATTCGCCATGTCATAAATATTCAGCCCAATACT
13	AAAATCAGTTCATCAACGTCTGGCCTTCCTGTAATTGCGT
14	ACGTTGGTGGATTGACCGTAATGGATATTATTTGCACGTAAGGTTTA
15	GGGGACGACGACAGTAAGTTAGCGGCGGAATCTGCAAAAG
16	TCAGGCTGCGCAACTTTTTTTTGGGAAGGGCGATGGCAAAGCGC
17	CGTCACCAGTACTTTCCGGCACCGCTTCTGGTGCCTGCTGCAA
18	TACATTTAACAAACGGATAACCTCACCGGAAACAAAGCGGATC
19	CGGTGGTGCCATTAGTGATGAAGGGTAAAGTTAAAGATAGGTC
20	GTCTTTACTGATACTAATAGTAGT
21	GCCACCGCCACCCTCAGAGAGCCCAATAATACGAGGAAAGTGTATCATTTTTTTTTTTTTTT**AAAAGGTGAGATGAT**
22	ATCATACAGGCAAGGGGAACGCCATCAGCTCATTTTTTTTTTTTTTTTTTTT**AAAAGGTGAGATGAT**
23	ACGTCGCGCAGAGGCGAATACAGGAGGCCGACAAATTAATACATCGGTTTTTTTTTTTTTT**AAAAGGTGAGATGAT**
24	TCATAGCCCCCTTATCGGATAAGGCGGGGTTTTGCTCATTTTTTTTTTTTTT**AAAAGGTGAGATGAT**
25	ACCCGTCGGATTCTCAGCCAGCTTTTTTTTTTTTTTT**AAAAGGTGAGATGAT**
26	CAATCAAAAAAATCTAAAGCATAGATTTTCAAACAGATTTTTTTTTTTTTT**AAAAGGTGAGATGAT**
27	GAGAGGGTCCGTACTCAGGCGAAGCCCTTTTTTTAATTTTTTTTTTTTTTTT**AAAAGGTGAGATGAT**
28	ACGTCAGAGAGAAACAATACTGAGAAGTGTTTTTTTTTTTTTTTTTTTTTTT**AAAAGGTGAGATGAT**
29	ATAATTCGCATTAAATGTGATTGAATCCCTTTTTCTCATTTTTTTTTTTTTT**AAAAGGTGAGATGAT**

Supplementary Table 18 | List of staples in internal linker mix 12 (I12). Highlighted sequences are complementary to functionalised oligonucleotides (Supplementary Table 3).

No.	E1 sequences (external mix 1)
01	CTTATTACGTGGCAACATATAAAACATATGGTCAGAGAGACGAGCGTC
02	CAAAATCAAACGTCACAGAAAATTGAAACGCAGAGAATTAAACCCACA
03	AAACATTCAAAAACATGAACGGTCGAACTGACAGACCAG
04	AATCGTAACAAACAAGGGAGAGGGTAGCTATTTTTGAGAAGGCCGGA
05	TTTAATGGAGCCAGTAATAAGAGAGTATTCTAAACAGCCATA
06	CAATGAAACCATCAAGGGTGAGAAGATCTACAAAGGCATTAGAGCCAG
07	TTACCAGCGAATTATCACCATTACTATTCACAATTTTTCAAATAAA
08	ATACCGGGCACATTAATTGCGTTGTCCACTATGGAAAGCCGG
09	TAGTCTTTATTAAAAAAATCGTCTTTGCAACACGCCGCTAAGAATCAG
10	CCCTCAGCCCCTGCCACTGGTAACAATAATCATTAAACC
11	TAAGTTTTGATGATACAAAGCCAGAATGGAAAGCGCAGTTTGACAGG
12	TACCGAACGAACCCGCCGCCAGCACTCTGAATTTACCTGAATGGCTAT
13	AGTAGAAGATATTACCGCCAGCCAGAAATGGAGTCACGCTTCGGAACC
14	TTATTTACGAAAGCGTAACTGATAATTTAGAAGTTTTTATTAGACT
15	CGCATAACAAGAGGCAAAAGAATATGCCCTGAACATTATTAC
16	AAAAGATTTTCGAGCTTCAAAGCGCTCCTTTTAAGGAATTGGAAGAAA
17	GTTGAAATCAATATAAAAGAAACCCTGAGAGCAGCAGGC
18	ATGGTCAATAACCAGCACTAACAACTTTGCCCGAACGGTTGATTCCCA
19	ATTCTGCGTTTCGCAATTTAATTGAACCAGACCCTCGTTTCCAGAGGG
20	CACCAGAATATCATTTAATTCGACAACTCGTATTAAATCCTAATAGA
21	GATAAGAGAAGTACGGGATTTAGTACCGTTCTATTTTTCTGATAAA

Supplementary Table 19 | List of staples in external linker mix 1 (E1).

No.	E2 sequences (external mix 2)
01	CAAAATCAAACGTCACAGAAAATTGAAACGCAGAGAATTAAACCCACA
02	CAATGAAACCATCAAGGGTGAGAAGATCTACAAAGGCATTAGAGCCAG
03	TAGTCTTTATTAAAAAAATCGTCTTTGCAACACGCCGCTAAGAATCAG
04	TACCGAACGAACCCGCCGCCAGCACTCTGAATTTACCTGAATGGCTAT
05	ATGGTCAATAACCAGCACTAACAACTTTGCCCGAACGGTTGATTCCCA
06	ATTCTGCGTTTCGCAATTTAATTGAACCAGACCCTCGTTTCCAGAGGG
07	AATCGTAACAAACAAGGGAGAGG
08	TTACCAGCGAATTATCACCATTAC
09	TAAGTTTTGATGATACAAAGCCA
10	TTATTTACGAAAGCGTAACTGATA
11	CACCAGAATATCATTTAATTCGA
12	GATAAGAGAAGTACGGGATTTAGT
13	**TTTCAGGCTGTAGCTTGG**TTTGTAGCTATTTTTGAGAAGGCCGGA
14	**TTTCAGGCTGTAGCTTGG**TTTTATTCACAATTTTTCAAATAAA
15	**TTTCAGGCTGTAGCTTGG**TTTGAATGGAAAGCGCAGTTTGACAGG
16	**TTTCAGGCTGTAGCTTGG**TTTATTTAGAAGTTTTTATTAGACT
17	**TTTCAGGCTGTAGCTTGG**TTTCAACTCGTATTAAATCCTAATAGA
18	**TTTCAGGCTGTAGCTTGG**TTTACCGTTCTATTTTTCTGATAAA
19	AAACATTCAAAAACATGAACGGTCGAACTGACAGACCAG**TTTCAGGCTGTAGCTTGG**TTT
20	TTTAATGGAGCCAGTAATAAGAGAGTATTCTAAACAGCCATA**TTTCAGGCTGTAGCTTGG**TTT
21	CCCTCAGCCCCTGCCACTGGTAACAATAATCATTAAACC**TTTCAGGCTGTAGCTTGG**TTT
22	ATACCGGGCACATTAATTGCGTTGTCCACTATGGAAAGCCGG**TTTCAGGCTGTAGCTTGG**TTT
23	GTTGAAATCAATATAAAAGAAACCCTGAGAGCAGCAGGC**TTTCAGGCTGTAGCTTGG**TTT
24	CGCATAACAAGAGGCAAAAGAATATGCCCTGAACATTATTAC**TTTCAGGCTGTAGCTTGG**TTT
25	CTTATTACGTGGCAACATATAAAACATATGGTCAGAGAGACGAGCGTCTTT**GATGTGTAGAGT****TGGAGGTGATAG**
26	AGTAGAAGATATTACCGCCAGCCAGAAATGGAGTCACGCTTCGGAACCTTT**GATGTGTAGAGT****TGGAGGTGATAG**
27	AAAAGATTTTCGAGCTTCAAAGCGCTCCTTTTAAGGAATTGGAAGAAATTT**GATGTGTAGAGT****TGGAGGTGATAG**

Supplementary Table 20 | List of staples in external linker mix 2 (E2). Highlighted sequences are complementary to functionalised oligonucleotides (Supplementary Tables 3 & 4).

No.	E3 sequences (external mix 3)
01	CAAAATCAAACGTCACAGAAAATTGAAACGCAGAGAATTAAACCCACA
02	CAATGAAACCATCAAGGGTGAGAAGATCTACAAAGGCATTAGAGCCAG
03	TAGTCTTTATTAAAAAAATCGTCTTTGCAACACGCCGCTAAGAATCAG
04	TACCGAACGAACCCGCCGCCAGCACTCTGAATTTACCTGAATGGCTAT
05	ATGGTCAATAACCAGCACTAACAACTTTGCCCGAACGGTTGATTCCCA
06	ATTCTGCGTTTCGCAATTTAATTGAACCAGACCCTCGTTTCCAGAGGG
07	AATCGTAACAAACAAGGGAGAGG
08	TTACCAGCGAATTATCACCATTAC
09	TAAGTTTTGATGATACAAAGCCA
10	TTATTTACGAAAGCGTAACTGATA
11	CACCAGAATATCATTTAATTCGA
12	GATAAGAGAAGTACGGGATTTAGT
13	**TTTGCGAGGCGGACTGGC**TTTGTAGCTATTTTTGAGAAGGCCGGA
14	**TTTGCGAGGCGGACTGGC**TTTTATTCACAATTTTTCAAATAAA
15	**TTTGCGAGGCGGACTGGC**TTTGAATGGAAAGCGCAGTTTGACAGG
16	**TTTGCGAGGCGGACTGGC**TTTATTTAGAAGTTTTTATTAGACT
17	**TTTGCGAGGCGGACTGGC**TTTCAACTCGTATTAAATCCTAATAGA
18	**TTTGCGAGGCGGACTGGC**TTTACCGTTCTATTTTTCTGATAAA
19	AAACATTCAAAAACATGAACGGTCGAACTGACAGACCAG**TTTGCGAGGCGGACTGGC**TTT
20	CTTATTACGTGGCAACATATAAAACATATGGTCAGAGAGACGAGCGTC**TTTGCGAGGCGGACTGGC**TTT
21	TTTAATGGAGCCAGTAATAAGAGAGTATTCTAAACAGCCATA**TTTGCGAGGCGGACTGGC**TTT
22	CCCTCAGCCCCTGCCACTGGTAACAATAATCATTAAACC**TTTGCGAGGCGGACTGGC**TTT
23	AGTAGAAGATATTACCGCCAGCCAGAAATGGAGTCACGCTTCGGAACC**TTTGCGAGGCGGACTGGC**TTT
24	ATACCGGGCACATTAATTGCGTTGTCCACTATGGAAAGCCGG**TTTGCGAGGCGGACTGGC**TTT
25	GTTGAAATCAATATAAAAGAAACCCTGAGAGCAGCAGGC**TTTGCGAGGCGGACTGGC**TTT
26	AAAAGATTTTCGAGCTTCAAAGCGCTCCTTTTAAGGAATTGGAAGAAA**TTTGCGAGGCGGACTGGC**TTT
27	CGCATAACAAGAGGCAAAAGAATATGCCCTGAACATTATTAC**TTTGCGAGGCGGACTGGC**TTT

Supplementary Table 21 | List of staples in external linker mix 3 (E3). Highlighted sequences are complementary to functionalised oligonucleotides (Supplementary Table 3).

No.	E4 sequences (external mix 4)
01	CTTATTACGTGGCAACATATAAAACATATGGTCAGAGAGACGAGCGTC
02	AAACATTCAAAAACATGAACGGTCGAACTGACAGACCAG
03	AATCGTAACAAACAAGGGAGAGGGTAGCTATTTTTGAGAAGGCCGGA
04	TTTAATGGAGCCAGTAATAAGAGAGTATTCTAAACAGCCATA
05	TTACCAGCGAATTATCACCATTACTATTCACAATTTTTCAAATAAA
06	ATACCGGGCACATTAATTGCGTTGTCCACTATGGAAAGCCGG
07	CCCTCAGCCCCTGCCACTGGTAACAATAATCATTAAACC
08	TAAGTTTTGATGATACAAAGCCAGAATGGAAAGCGCAGTTTGACAGG
09	AGTAGAAGATATTACCGCCAGCCAGAAATGGAGTCACGCTTCGGAACC
10	TTATTTACGAAAGCGTAACTGATAATTTAGAAGTTTTTATTAGACT
11	CGCATAACAAGAGGCAAAAGAATATGCCCTGAACATTATTAC
12	AAAAGATTTTCGAGCTTCAAAGCGCTCCTTTTAAGGAATTGGAAGAAA
13	GTTGAAATCAATATAAAAGAAACCCTGAGAGCAGCAGGC
14	CACCAGAATATCATTTAATTCGACAACTCGTATTAAATCCTAATAGA
15	GATAAGAGAAGTACGGGATTTAGTACCGTTCTATTTTTCTGATAAA
16	AGAGCCAGCAAAATCAAACGTCACAGAAAATTGAAACGCAGAGAATTAAACCCACA
17	ATGGCTATTAGTCTTTATTAAAAAAATCGTCTTTGCAACACGCCGCTAAGAATCAG
18	GATTCCCAATTCTGCGTTTCGCAATTTAATTGAACCAGACCCTCGTTTCCAGAGGG
19	CAATGAAACCATCAAGGGTGAGAAGATCTACAAAGGCATTTTTTTTTTTTTTTTTTTTTT**AAAGAGTGAGAGGTGGAG**
20	TACCGAACGAACCCGCCGCCAGCACTCTGAATTTACCTGATTTTTTTTTTTTTTTTTTTT**AAAGAGTGAGAGGTGGAG**
21	ATGGTCAATAACCAGCACTAACAACTTTGCCCGAACGGTTTTTTTTTTTTTTTTTTTTTT**AAAGAGTGAGAGGTGGAG**

Supplementary Table 22 | List of staples in external linker mix 4 (E4). Highlighted sequences are complementary to functionalised oligonucleotides (Supplementary Table 3).

No.	E5 sequences (external mix 5)
01	CAAAATCAAACGTCACAGAAAATTGAAACGCAGAGAATTAAACCCACA
02	CAATGAAACCATCAAGGGTGAGAAGATCTACAAAGGCATTAGAGCCAG
03	TAGTCTTTATTAAAAAAATCGTCTTTGCAACACGCCGCTAAGAATCAG
04	TACCGAACGAACCCGCCGCCAGCACTCTGAATTTACCTGAATGGCTAT
05	ATGGTCAATAACCAGCACTAACAACTTTGCCCGAACGGTTGATTCCCA
06	ATTCTGCGTTTCGCAATTTAATTGAACCAGACCCTCGTTTCCAGAGGG
07	AATCGTAACAAACAAGGGAGAGG
08	TTACCAGCGAATTATCACCATTAC
09	TAAGTTTTGATGATACAAAGCCA
10	TTATTTACGAAAGCGTAACTGATA
11	CACCAGAATATCATTTAATTCGA
12	GATAAGAGAAGTACGGGATTTAGT
13	**AAAAGGTGAGATGAT**TTTGTAGCTATTTTTGAGAAGGCCGGA
14	**AAAAGGTGAGATGAT**TTTTATTCACAATTTTTCAAATAAA
15	**AAAAGGTGAGATGAT**TTTGAATGGAAAGCGCAGTTTGACAGG
16	**AAAAGGTGAGATGAT**TTTATTTAGAAGTTTTTATTAGACT
17	**AAAAGGTGAGATGAT**TTTCAACTCGTATTAAATCCTAATAGA
18	**AAAAGGTGAGATGAT**TTTACCGTTCTATTTTTCTGATAAA
19	AAACATTCAAAAACATGAACGGTCGAACTGACAGACCAG**TTTCAGGCTGTAGCTTGG**TTT
20	CTTATTACGTGGCAACATATAAAACATATGGTCAGAGAGACGAGCGTC**TTTCAGGCTGTAGCTTGG**TTT
21	TTTAATGGAGCCAGTAATAAGAGAGTATTCTAAACAGCCATA**TTTCAGGCTGTAGCTTGG**TTT
22	CCCTCAGCCCCTGCCACTGGTAACAATAATCATTAAACC**TTTCAGGCTGTAGCTTGG**TTT
23	AGTAGAAGATATTACCGCCAGCCAGAAATGGAGTCACGCTTCGGAACC**TTTCAGGCTGTAGCTTGG**TTT
24	ATACCGGGCACATTAATTGCGTTGTCCACTATGGAAAGCCGG**TTTCAGGCTGTAGCTTGG**TTT
25	GTTGAAATCAATATAAAAGAAACCCTGAGAGCAGCAGGC**TTTCAGGCTGTAGCTTGG**TTT
26	AAAAGATTTTCGAGCTTCAAAGCGCTCCTTTTAAGGAATTGGAAGAAA**TTTCAGGCTGTAGCTTGG**TTT
27	CGCATAACAAGAGGCAAAAGAATATGCCCTGAACATTATTAC**TTTCAGGCTGTAGCTTGG**TTT

Supplementary Table 23 | List of staples in external linker mix 5 (E5). Highlighted sequences are complementary to functionalised oligonucleotides (Supplementary Table 3).

No.	E6 sequences (external mix 6)
01	CTTATTACGTGGCAACATATAAAACATATGGTCAGAGAGACGAGCGTC
02	CAAAATCAAACGTCACAGAAAATTGAAACGCAGAGAATTAAACCCACA
03	AAACATTCAAAAACATGAACGGTCGAACTGACAGACCAG
04	TTTAATGGAGCCAGTAATAAGAGAGTATTCTAAACAGCCATA
05	CAATGAAACCATCAAGGGTGAGAAGATCTACAAAGGCATTAGAGCCAG
06	ATACCGGGCACATTAATTGCGTTGTCCACTATGGAAAGCCGG
07	TAGTCTTTATTAAAAAAATCGTCTTTGCAACACGCCGCTAAGAATCAG
08	CCCTCAGCCCCTGCCACTGGTAACAATAATCATTAAACC
09	TACCGAACGAACCCGCCGCCAGCACTCTGAATTTACCTGAATGGCTAT
10	AGTAGAAGATATTACCGCCAGCCAGAAATGGAGTCACGCTTCGGAACC
11	CGCATAACAAGAGGCAAAAGAATATGCCCTGAACATTATTAC
12	AAAAGATTTTCGAGCTTCAAAGCGCTCCTTTTAAGGAATTGGAAGAAA
13	GTTGAAATCAATATAAAAGAAACCCTGAGAGCAGCAGGC
14	ATGGTCAATAACCAGCACTAACAACTTTGCCCGAACGGTTGATTCCCA
15	ATTCTGCGTTTCGCAATTTAATTGAACCAGACCCTCGTTTCCAGAGGG
16	AATCGTAACAAACAAGGGAGAGG
17	TTACCAGCGAATTATCACCATTAC
18	TAAGTTTTGATGATACAAAGCCA
19	TTATTTACGAAAGCGTAACTGATA
20	CACCAGAATATCATTTAATTCGA
21	GATAAGAGAAGTACGGGATTTAGT
22	**TTTCAGGCTGTAGCTTGG**TTTGTAGCTATTTTTGAGAAGGCCGGA
23	**TTTCAGGCTGTAGCTTGG**TTTTATTCACAATTTTTCAAATAAA
24	**TTTCAGGCTGTAGCTTGG**TTTGAATGGAAAGCGCAGTTTGACAGG
25	**TTTCAGGCTGTAGCTTGG**TTTATTTAGAAGTTTTTATTAGACT
26	**TTTCAGGCTGTAGCTTGG**TTTCAACTCGTATTAAATCCTAATAGA
27	**TTTCAGGCTGTAGCTTGG**TTTACCGTTCTATTTTTCTGATAAA

Supplementary Table 24 | List of staples in external linker mix 6 (E6). Highlighted sequences are complementary to functionalised oligonucleotides (Supplementary Table 3).

Osmolality difference	Glycine (mM)	$MgCl_2$ (mM)	NaCl (mM)	Total volume (µl)
**Isotonic ctrl.**	0	60	219.5	30
**-78%**	0	25	26.3	30
**-58%**	100	30	26.3	30
**-42%**	200	35	26.3	30
**-22%**	300	45	26.3	30
**-4**	400	50	26.3	30
**+12%**	500	55	26.3	30
**+33%**	600	65	26.3	30
**+54%**	700	75	26.3	30

Supplementary Table 25 | Tonicity screen sample compositions. Composition of the samples shown in Fig. 3a. The magnesium optimum for triangle assembly increased with increasing glycine concentrations. Concentrations above or below the optima presented in this table result in incomplete or impaired assembly. The isotonic control sample was conducted as a standard budding reaction, where triangles were tethered to the vesicles at high-salt conditions (5 mM $MgCl_2$, 300 mM NaCl). Addition of isotonic magnesium solution to trigger assembly lowered the overall NaCl concentration to 219.5 mM.

	TRIS (mM)	EDTA (mM)	$MgCl_2$ (mM)	NaCl (mM)	CsCl (mM)	Sucrose (mM)	Glucose (mM)	Glycine (mM)	pH
**Folding Buffer**	5	1	20	5					8.0
**Sodium Buffer**	5	1	5	305					8.0
**Caesium Buffer**	5	1	5	5	320				8.0
**Sucrose Buffer**	10	1				470			8.0
**Imaging Buffer A**	5	1	5	5		280			8.0
**Imaging Buffer B**	5	1	5	5			280		8.0
**Isotonic assembly buffer**	5	1	210	5					8.0
**Glycine buffer**	5	1	5					1000	6.8 (unadjusted)

Supplementary Table 26 | Buffer compositions

Supplementary References

1. Kanwa, N., Gavrilovic, S., Brüggenthies, G. A., Qutbuddin, Y. & Schwille, P. Inducing Lipid Domains in Membranes by Self-Assembly of DNA Origami. *Advanced Materials Interfaces* **10**, 2202500 (2023).
