# Supplementary material for: Programmable DNA shell scaffolds for directional membrane budding
Source: Nat Commun. 2025 Oct 9;16:8972. doi: 10.1038/s41467-025-64298-x (PMC12511405; doi:10.1038/s41467-025-64298-x)
Supplement: Supplementary file 2 — Description of Additional Supplementary Files [file 41467_2025_64298_MOESM2_ESM.pdf]

Description of Additional Supplementary Files

File name: Supplementary Movie 1
Description: Cryogenic tilt-series of a DNA-shell-coated vesicle. Shown is a scarred DNA-shell-coated vesicle at tilt angles from -45° to + 45° in steps of 2.5°. The visibility of the scar changes with the tilt angle. For tomography of this particle, refer to Supplementary Figure 17.
