# Supplementary material for: Programmable DNA shell scaffolds for directional membrane budding
Source: Nat Commun. 2025 Oct 9;16:8972. doi: 10.1038/s41467-025-64298-x (PMC12511405; doi:10.1038/s41467-025-64298-x)
Supplement: Supplementary file 4 — Reporting Summary [file 41467_2025_64298_MOESM4_ESM.pdf]

nature portfolio

Corresponding author(s): Hendrik Dietz

Last updated by author(s): Sep 11, 2025

Reporting Summary

Nature Portfolio wishes to improve the reproducibility of the work that we publish. This form provides structure for consistency and transparency in reporting. For further information on Nature Portfolio policies, see our Editorial Policies and the Editorial Policy Checklist.

Statistics

For all statistical analyses, confirm that the following items are present in the figure legend, table legend, main text, or Methods section.

n/a	Confirmed	
☐	☒	The exact sample size (*n*) for each experimental group/condition, given as a discrete number and unit of measurement
☐	☒	A statement on whether measurements were taken from distinct samples or whether the same sample was measured repeatedly
☒	☐	The statistical test(s) used AND whether they are one- or two-sided<br>*Only common tests should be described solely by name; describe more complex techniques in the Methods section.*
☒	☐	A description of all covariates tested
☒	☐	A description of any assumptions or corrections, such as tests of normality and adjustment for multiple comparisons
☐	☒	A full description of the statistical parameters including central tendency (e.g. means) or other basic estimates (e.g. regression coefficient) AND variation (e.g. standard deviation) or associated estimates of uncertainty (e.g. confidence intervals)
☒	☐	For null hypothesis testing, the test statistic (e.g. *F*, *t*, *r*) with confidence intervals, effect sizes, degrees of freedom and *P* value noted<br>*Give P values as exact values whenever suitable.*
☒	☐	For Bayesian analysis, information on the choice of priors and Markov chain Monte Carlo settings
☒	☐	For hierarchical and complex designs, identification of the appropriate level for tests and full reporting of outcomes
☒	☐	Estimates of effect sizes (e.g. Cohen's *d*, Pearson's *r*), indicating how they were calculated

Our web collection on statistics for biologists contains articles on many of the points above.

Software and code

Policy information about availability of computer code

Data collection	EPU v1.2 up to v2.6 (Thermo Fisher Scientific), Tomography 5 (Thermo Fisher Scientific), SerialEM v3.5.6, Invitrogen EVOS M7000 Cell Imaging System v2.4.1468.172
Data analysis	ImageJ/Fiji v2.16.0/1.54p, Kappa (ImageJ plugin) v2.0.0, IMOD Etomo v4.11.24, Prism v10.1.1 (Graphpad), caDNAno 2

For manuscripts utilizing custom algorithms or software that are central to the research but not yet described in published literature, software must be made available to editors and reviewers. We strongly encourage code deposition in a community repository (e.g. GitHub). See the Nature Portfolio guidelines for submitting code & software for further information.

Data

Policy information about availability of data

All manuscripts must include a data availability statement. This statement should provide the following information, where applicable:

- Accession codes, unique identifiers, or web links for publicly available datasets
- A description of any restrictions on data availability
- For clinical datasets or third party data, please ensure that the statement adheres to our policy

The data generated in this study are provided within the paper, Supplementary Information files and available from the corresponding author upon request. Unedited gel scans are provided as sourceData1 (pdf). Unedited gel scans, plots of band intensities and raw intensity data underlying Supplementary Figure 14 are provided as sourceData2 (excel). Source Data are provided with this paper.

Research involving human participants, their data, or biological material

Policy information about studies with human participants or human data. See also policy information about sex, gender (identity/presentation), and sexual orientation and race, ethnicity and racism.

Reporting on sex and gender	The research did not involve human participants.
Reporting on race, ethnicity, or other socially relevant groupings	The research did not involve human participants.
Population characteristics	The research did not involve human participants.
Recruitment	The research did not involve human participants.
Ethics oversight	The research did not involve human participants.

Note that full information on the approval of the study protocol must also be provided in the manuscript.

Field-specific reporting

Please select the one below that is the best fit for your research. If you are not sure, read the appropriate sections before making your selection.

[x] Life sciences [ ] Behavioural & social sciences [ ] Ecological, evolutionary & environmental sciences

For a reference copy of the document with all sections, see nature.com/documents/nr-reporting-summary-flat.pdf

Life sciences study design

All studies must disclose on these points even when the disclosure is negative.

Sample size	The study was explorative and focused on establishing and characterising a DNA-based budding machinery. Most experiments aimed to prove if budding is possible (mostly by TEM), and which factors influence the process (mostly gel electrophoresis & TEM). As the system is artificial in nature, no sampling was performed.<br>For fluorescence microscopy experiments, multiple fields of view were observed and acquired to obtain representative data for the individual sample. Any limitations to described effects are mentioned in figure captions or the main text.
Data exclusions	For the quantitative analysis of particle subspecies, particles with unclear morphology were excluded as stated in the figure caption. The number of excluded particles is mentioned. For analysis of DCVs by gel electrophoresis, any material in the gel pockets is not considered and for visualisation purposes, only the area beneath the pockets was used as reference for auto-levelling (thus oversaturating the pockets). This is stated in the methods section and the caption for Fig 2b which shows the first agarose gel in a main figure.
Replication	All attempts at replication were successful. The findings discussed in this study were reproduced at least two times. For the quantitative analysis of DCV yield by lipid quantity & vesicle size, each conditions was tested in three independent experiments, each using a fresh batch of lipid vesicles.
Randomization	Randomisation was not required in this proof-of-concept study, where the majority of results were not used for statistical analysis. All samples are defined in their composition and were treated identically (where possible). No living organisms have been used.<br>TEM images for the quantitative analysis of particle subspecies were acquired automatically, and the first 100 micrographs of each image stack was analysed. Particles were picked by hand due to the heterogenous nature of the particles.
Blinding	Blinding was not required in this proof-of-concept study, where the majority of results were not used for statistical analysis to justify blinding.

Reporting for specific materials, systems and methods

We require information from authors about some types of materials, experimental systems and methods used in many studies. Here, indicate whether each material, system or method listed is relevant to your study. If you are not sure if a list item applies to your research, read the appropriate section before selecting a response.

Materials & experimental systems

n/a	Involved in the study
x	Antibodies
x	Eukaryotic cell lines
x	Palaeontology and archaeology
x	Animals and other organisms
x	Clinical data
x	Dual use research of concern
x	Plants

Methods

n/a	Involved in the study
x	ChIP-seq
x	Flow cytometry
x	MRI-based neuroimaging

Plants

Seed stocks	n/a
Novel plant genotypes	n/a
Authentication	n/a
