# Supplementary material for: Programmable DNA shell scaffolds for directional membrane budding
Source: Nat Commun. 2025 Oct 9;16:8972. doi: 10.1038/s41467-025-64298-x (PMC12511405; doi:10.1038/s41467-025-64298-x)
Supplement: Supplementary file 6 — Source Data [file 41467_2025_64298_MOESM6_ESM.zip › sourceData1.pdf]

Source Data

Figure 2b

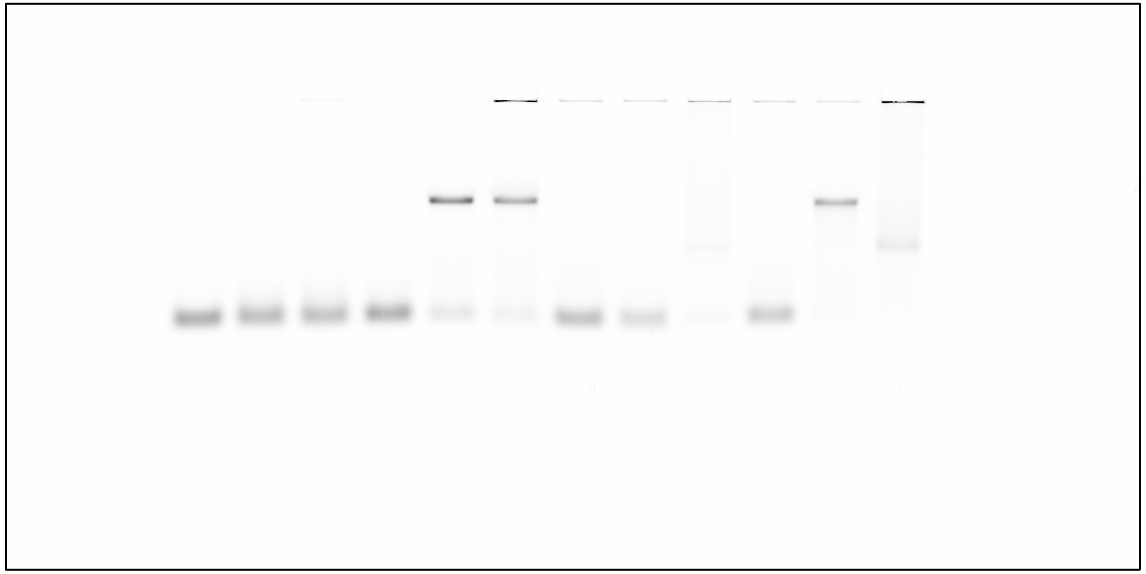

DNA-Atto643

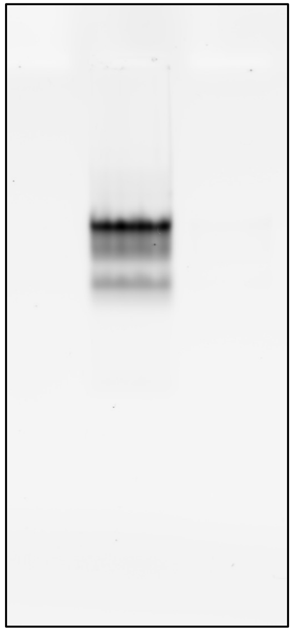

EtBr

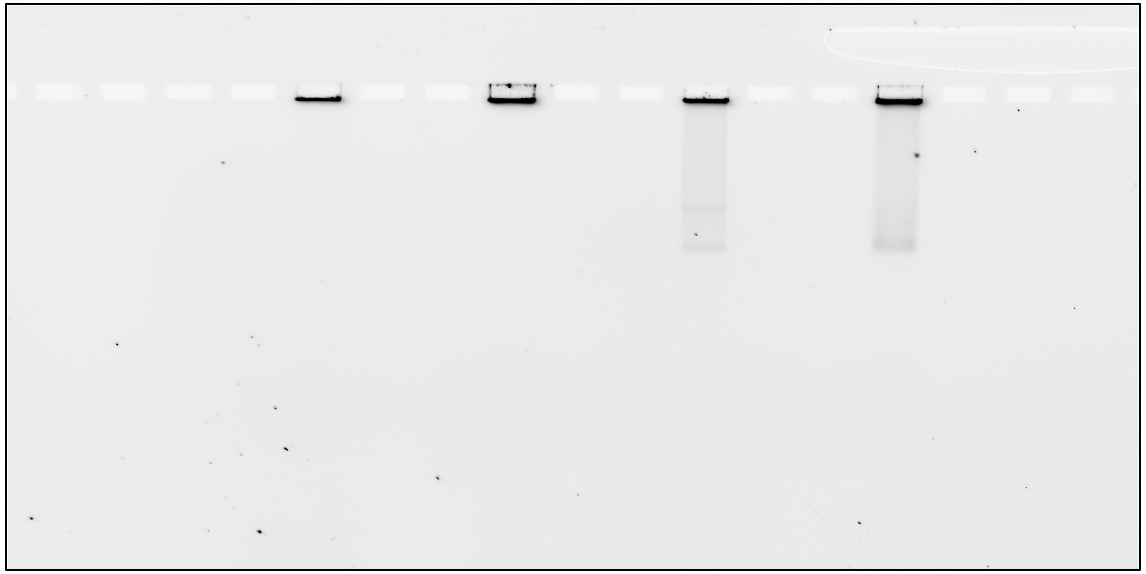

DOPE-Atto488

M. T. Pinner & H. Dietz, *Programmable DNA shell scaffolds for directional membrane budding,* Nat. Commun. 2025

Source Data

Figure 3a

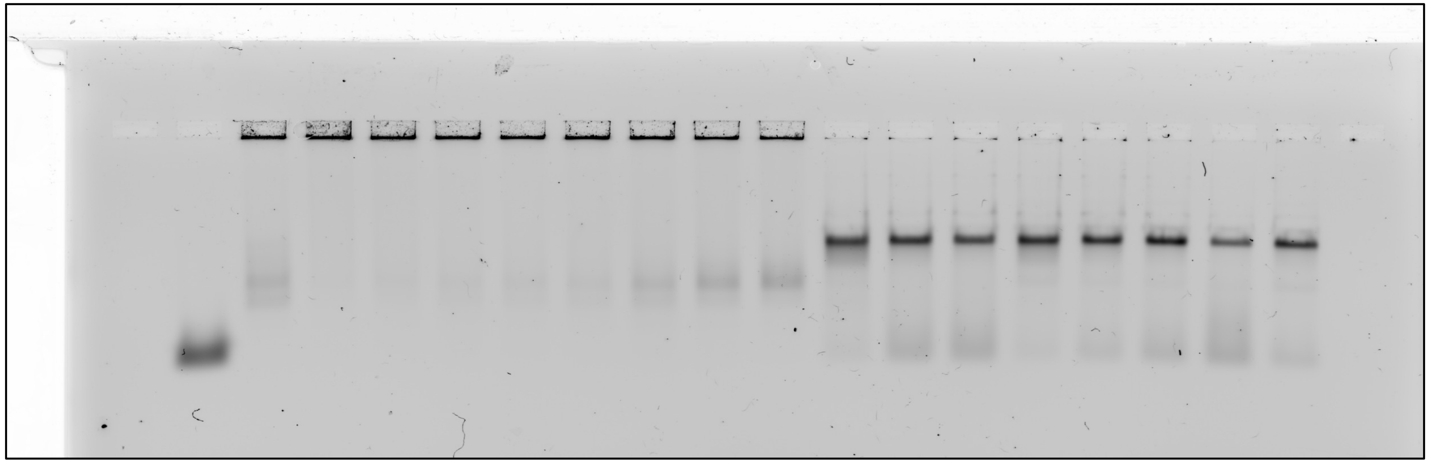

EtBr

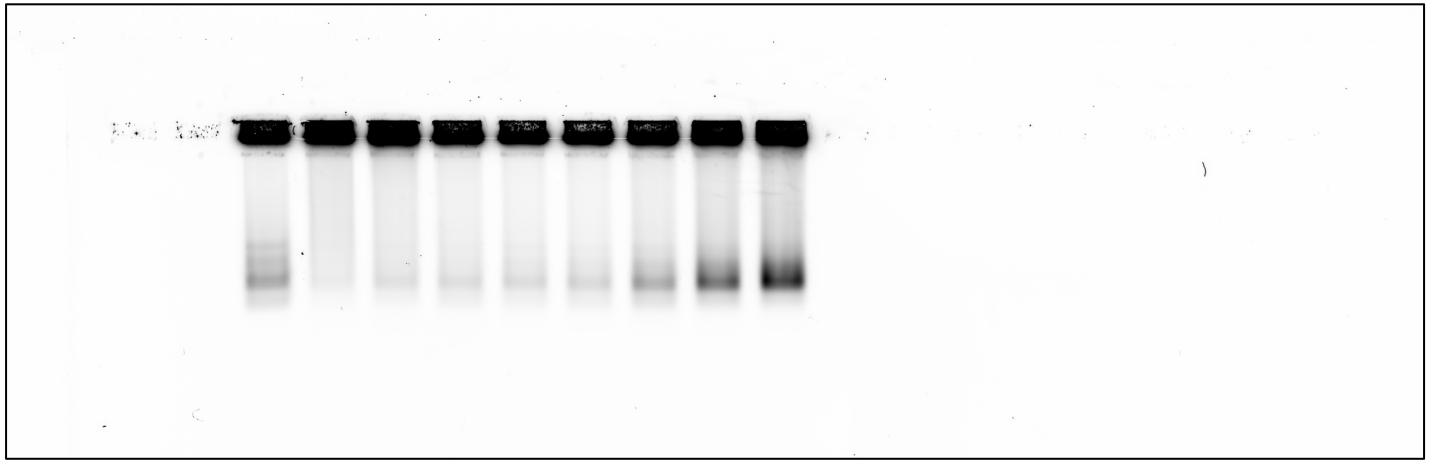

DOPE-Atto643

Source Data
Figure 3b

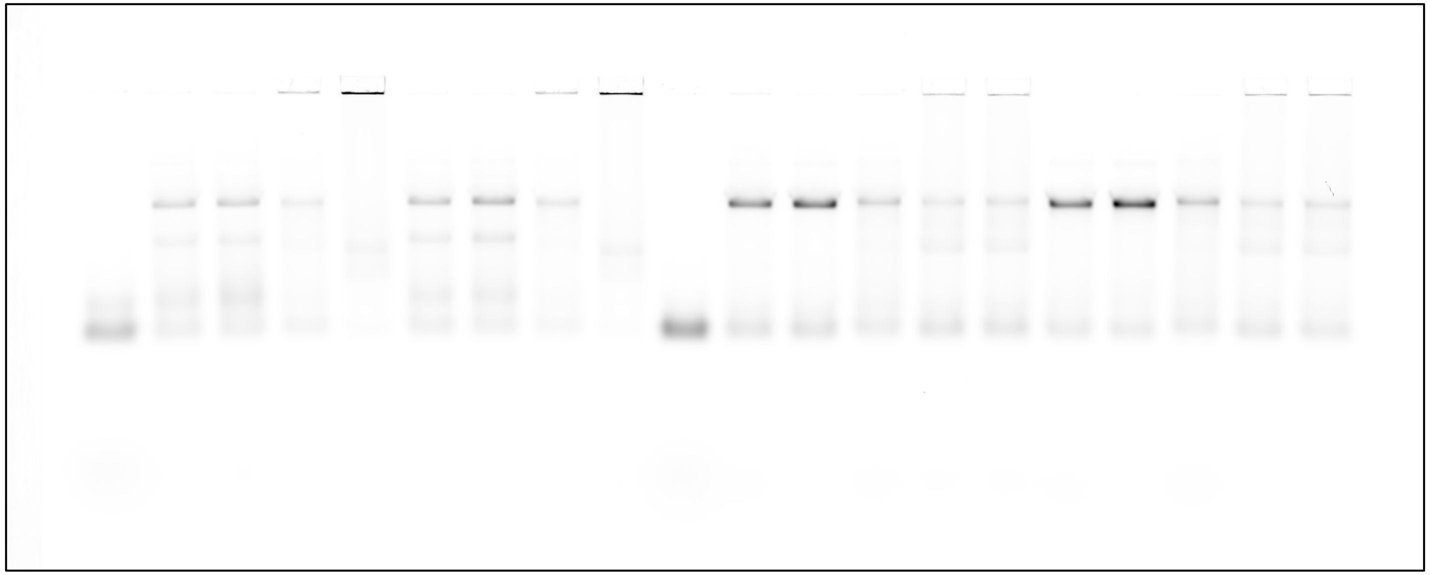

DNA-Atto643

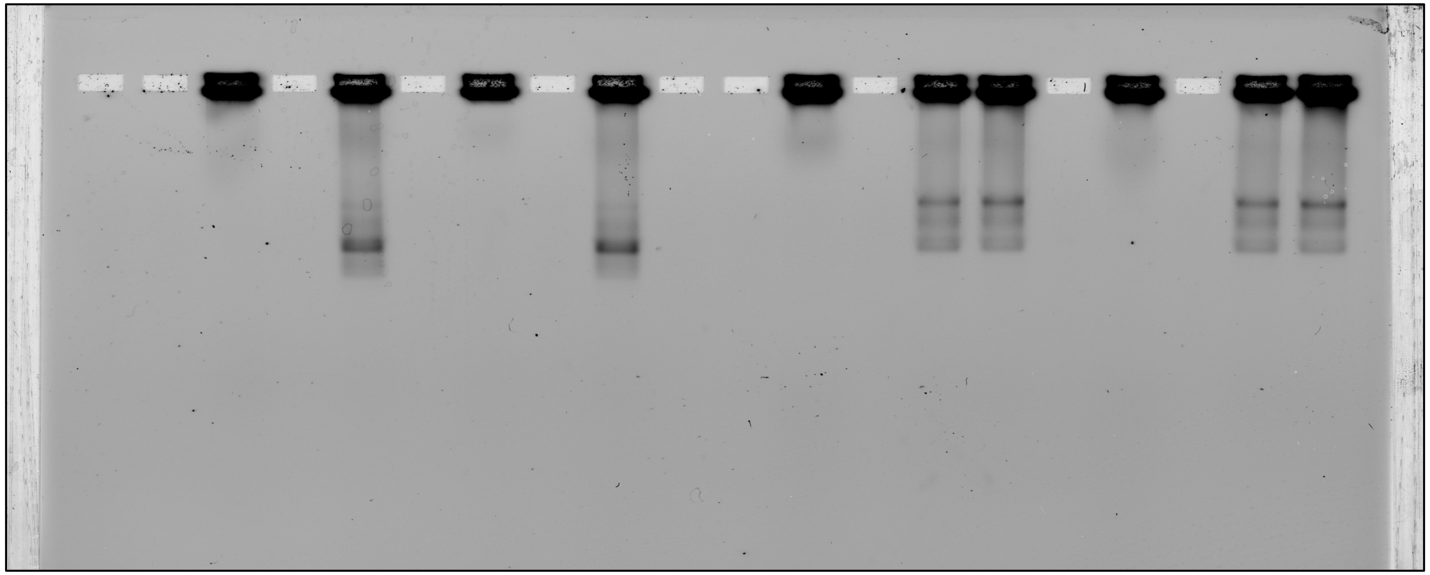

DOPE-Atto488

M. T. Pinner & H. Dietz, *Programmable DNA shell scaffolds for directional membrane budding,* Nat. Commun. 2025

Source Data

Figure 3c

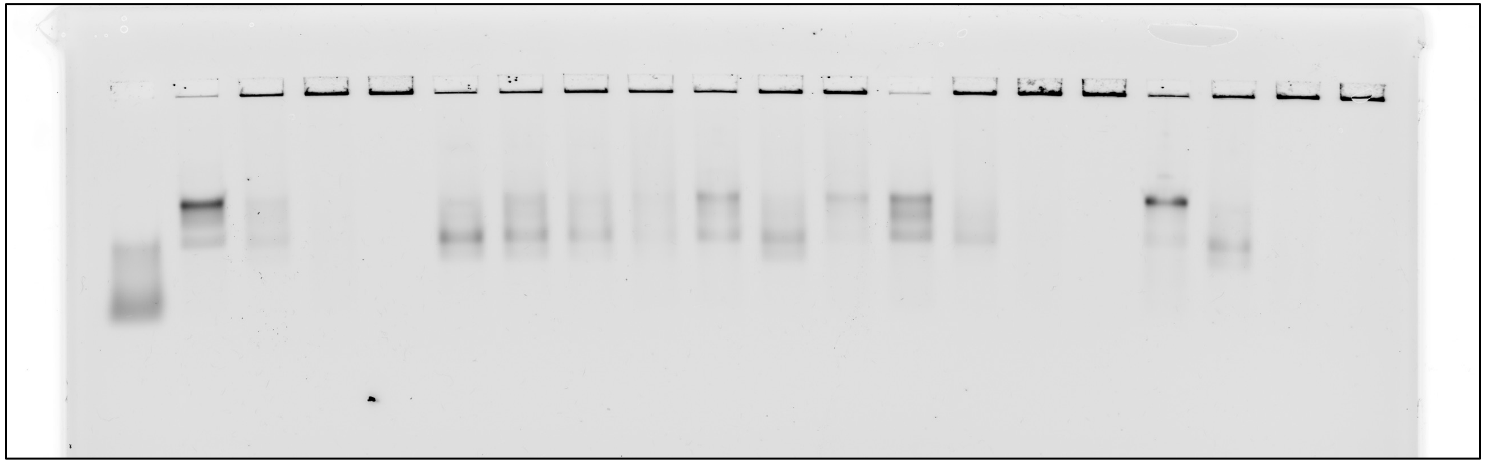

EtBr

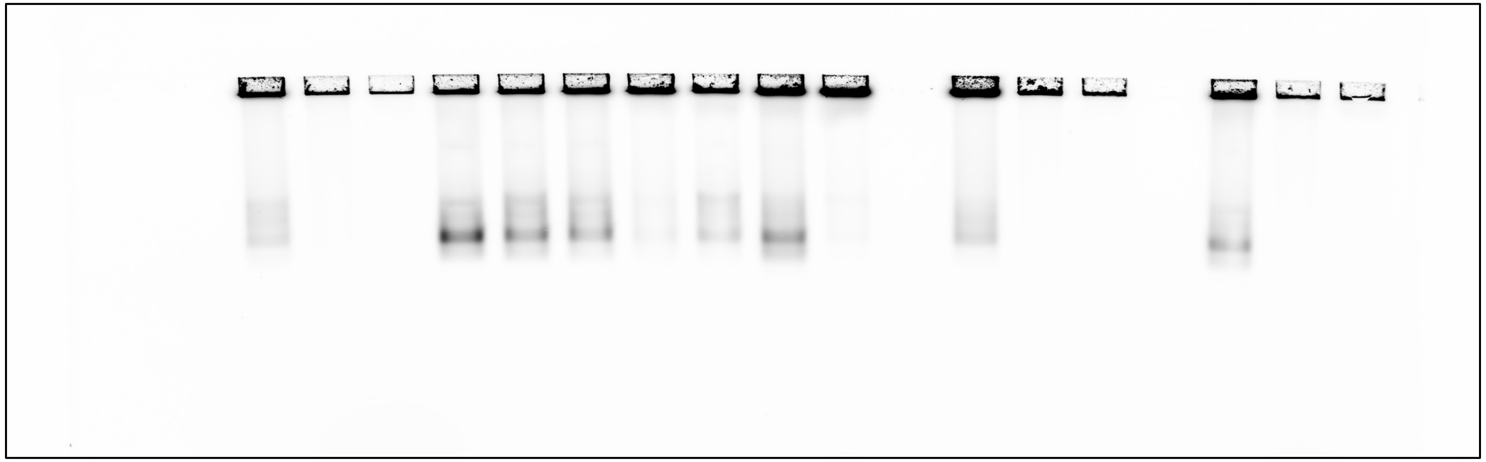

DOPE-Atto643

M. T. Pinner & H. Dietz, *Programmable DNA shell scaffolds for directional membrane budding,* Nat. Commun. 2025

Source Data

Figure 3d

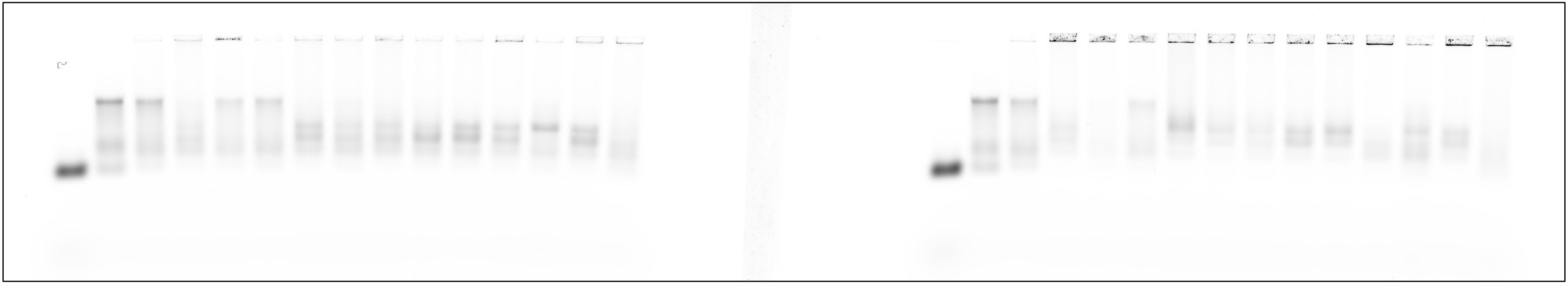

DNA-Atto643

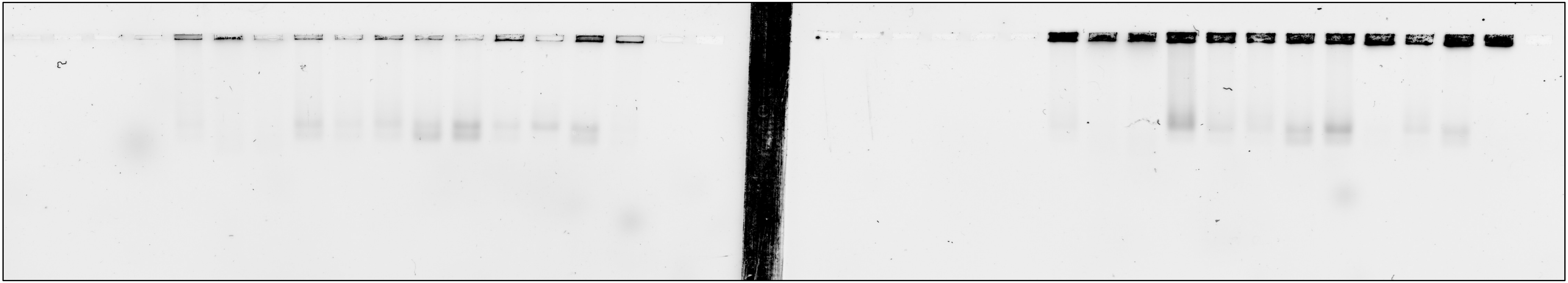

LissRhod-PE

M. T. Pinner & H. Dietz, *Programmable DNA shell scaffolds for directional membrane budding,* Nat. Commun. 2025

Source Data

Figure 3e (1)

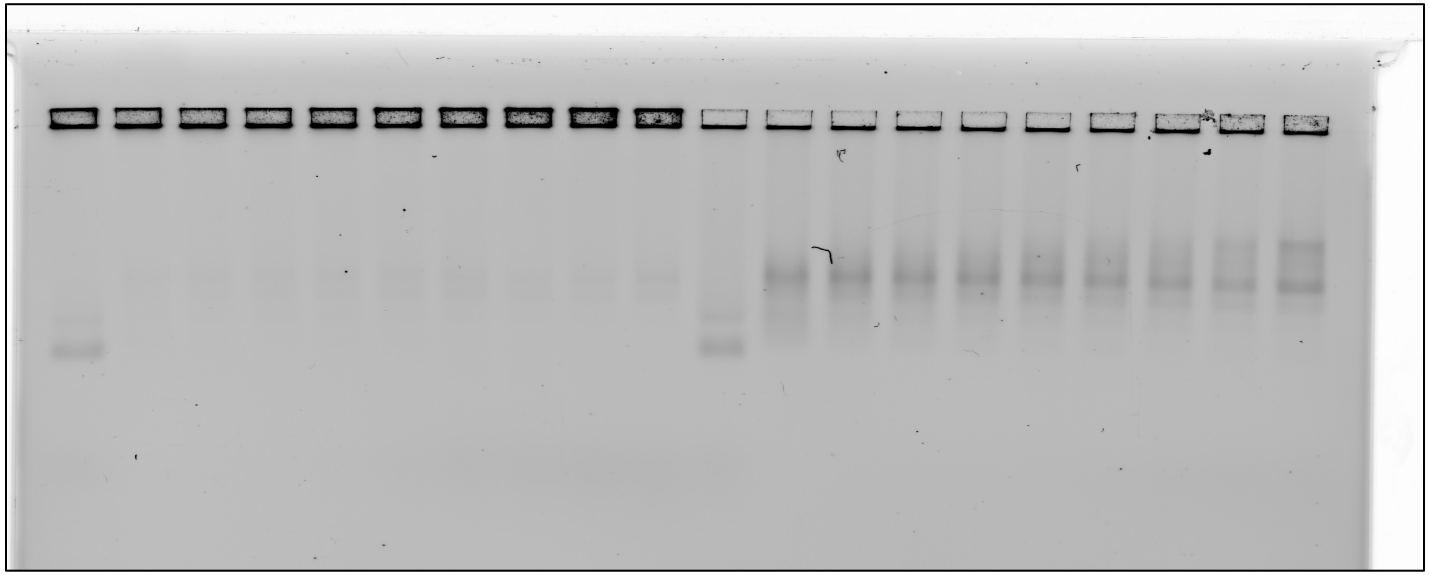

EtBr

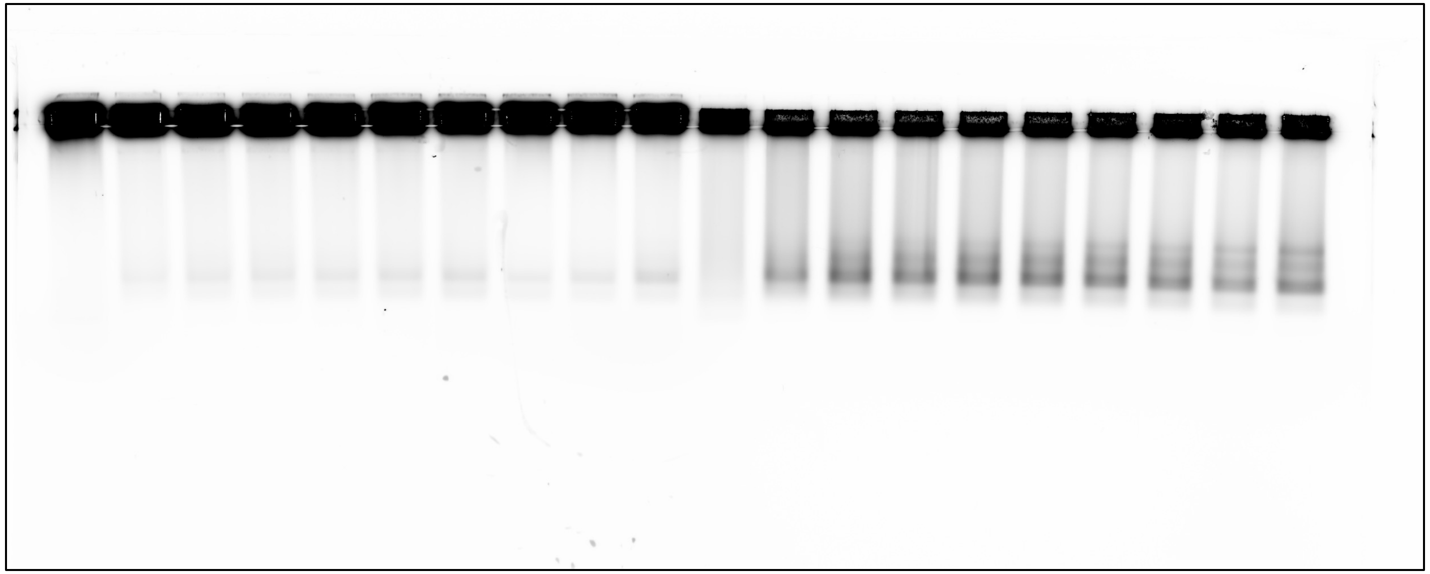

DOPE-Atto643

M. T. Pinner & H. Dietz, *Programmable DNA shell scaffolds for directional membrane budding,* Nat. Commun. 2025

Source Data

Figure 3e (2)

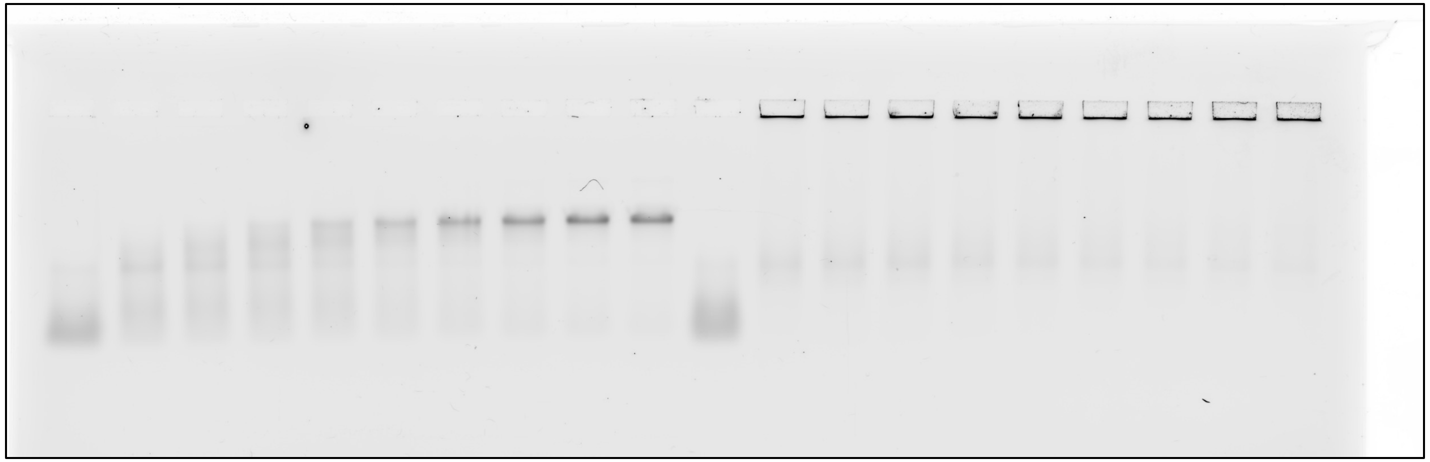

EtBr

M. T. Pinner & H. Dietz, *Programmable DNA shell scaffolds for directional membrane budding,* Nat. Commun. 2025

Source Data

Supplementary Figure 3b

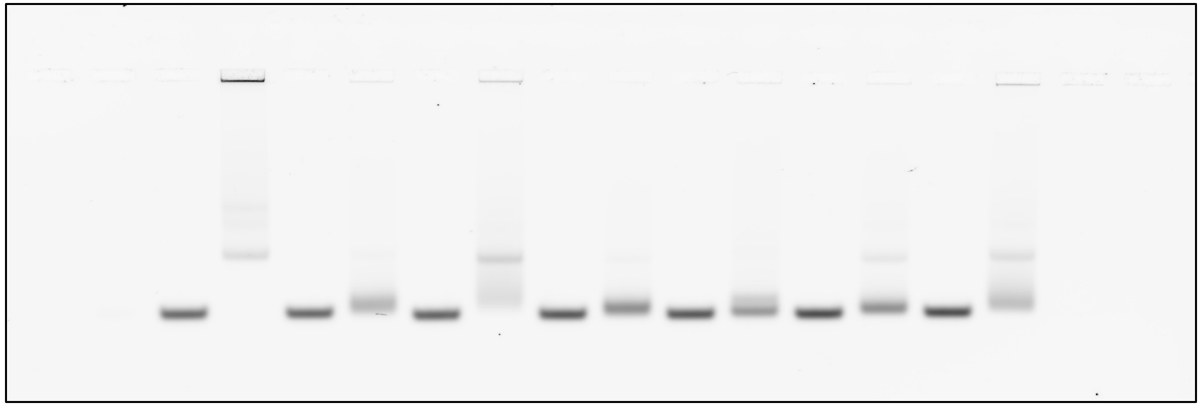

EtBr

M. T. Pinner & H. Dietz, *Programmable DNA shell scaffolds for directional membrane budding,* Nat. Commun. 2025

Source Data

Supplementary Figure 3c

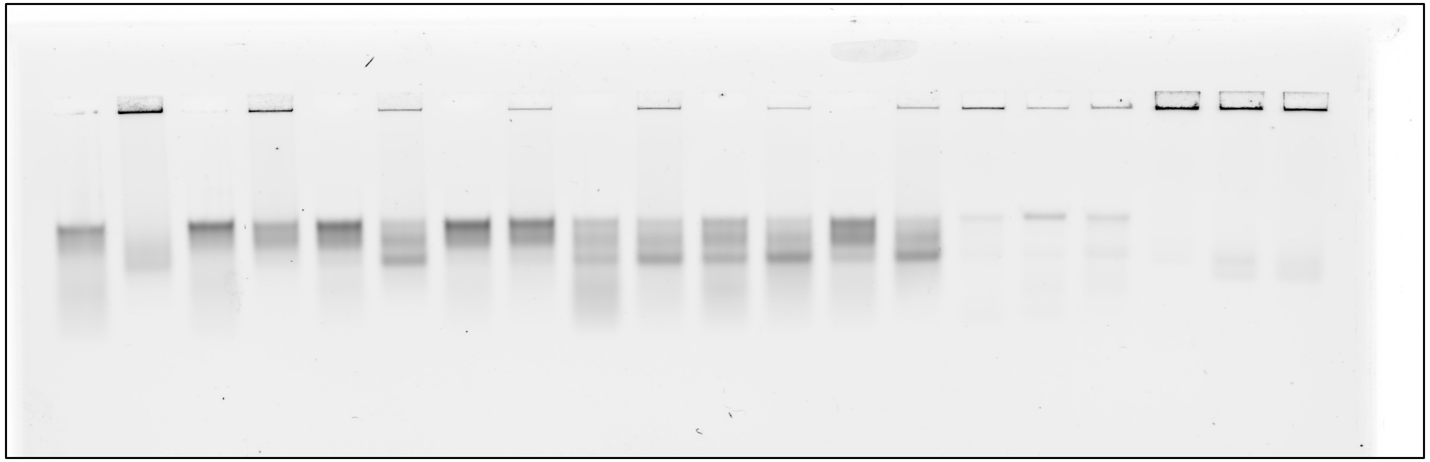

EtBr

Source Data

Supplementary Figure 4b

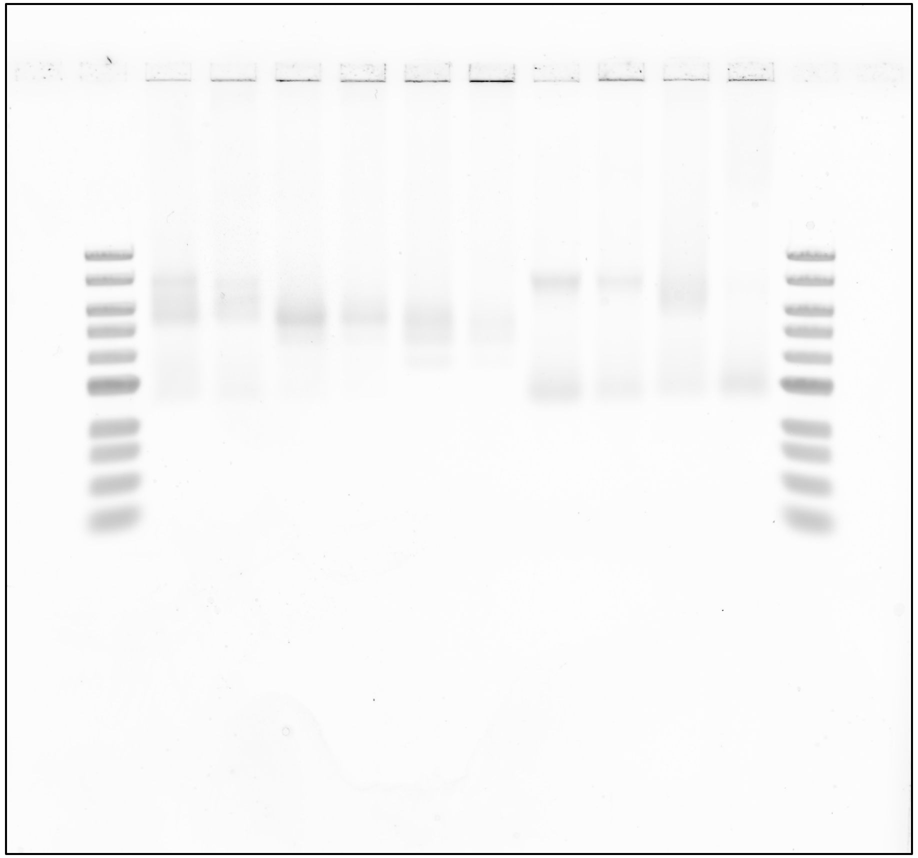

EtBr

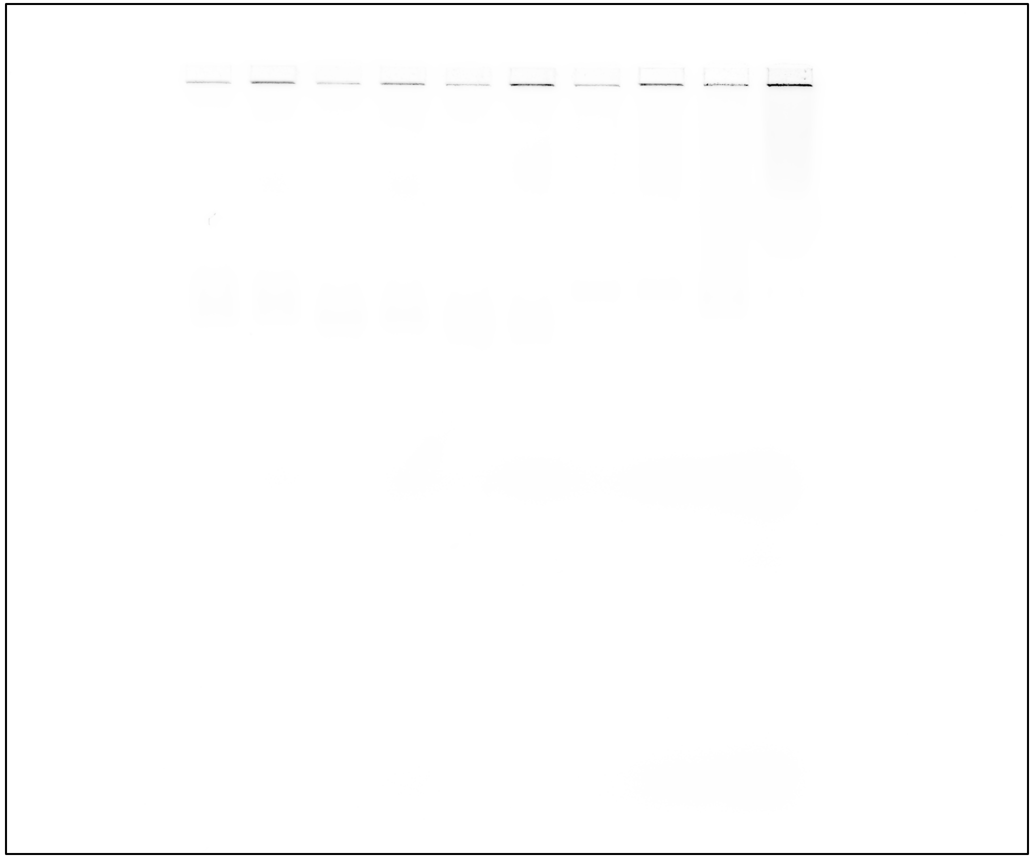

DOPE-Atto488

M. T. Pinner & H. Dietz, *Programmable DNA shell scaffolds for directional membrane budding,* Nat. Commun. 2025

Source Data

Supplementary Figure 5

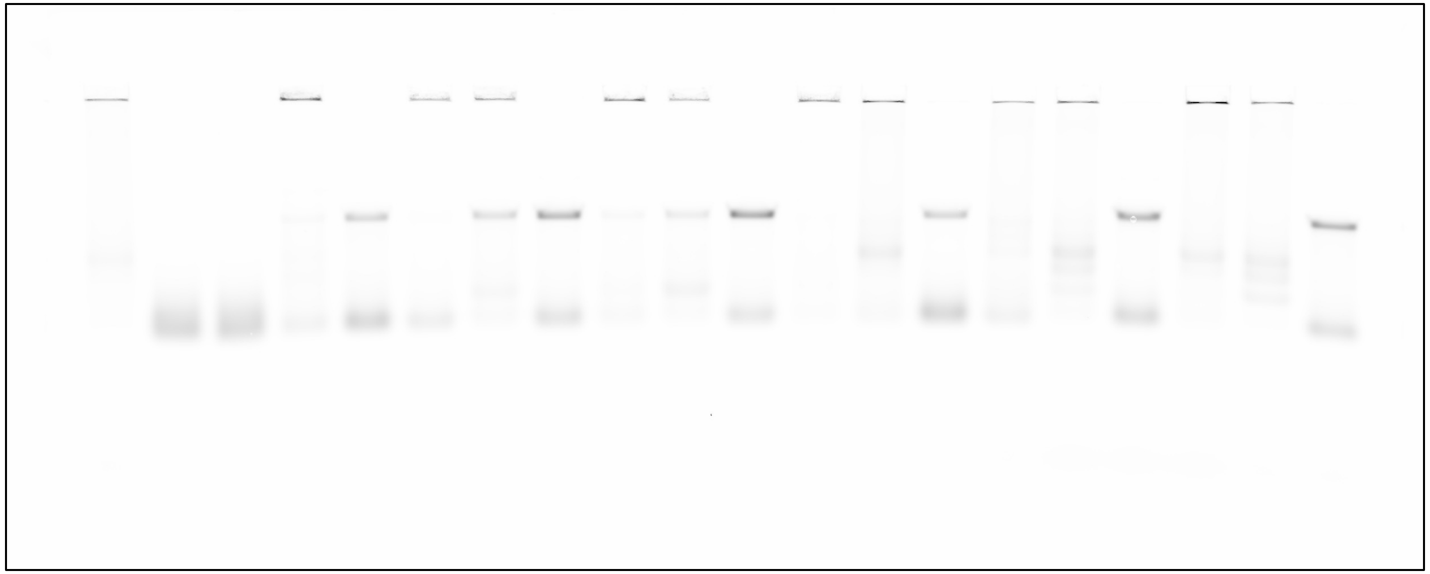

DNA-Atto643

M. T. Pinner & H. Dietz, *Programmable DNA shell scaffolds for directional membrane budding,* Nat. Commun. 2025

Source Data

Supplementary Figure 6

a

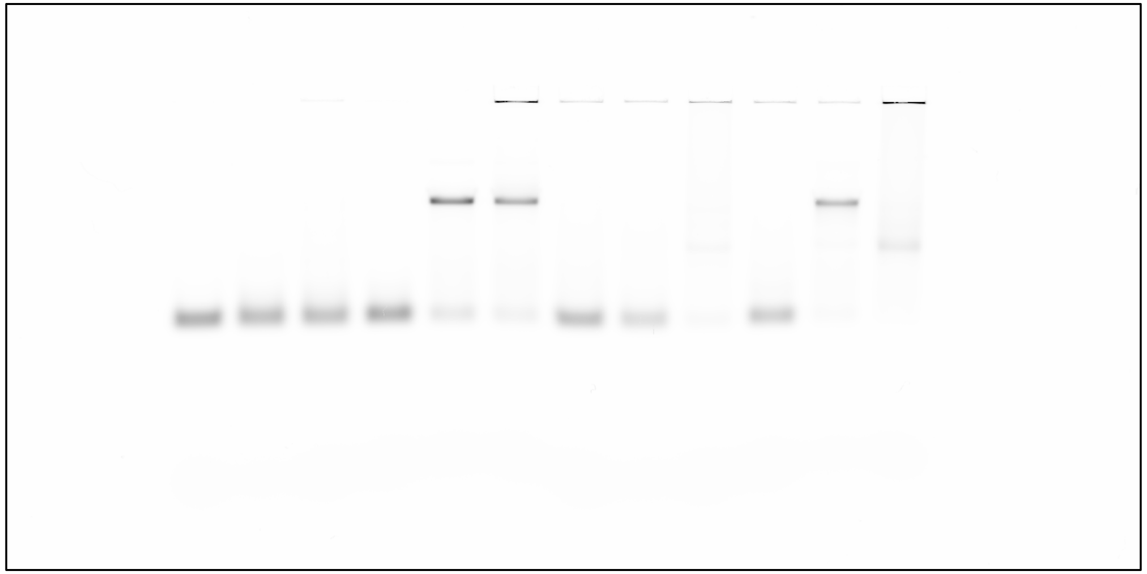

DNA-Atto643

b

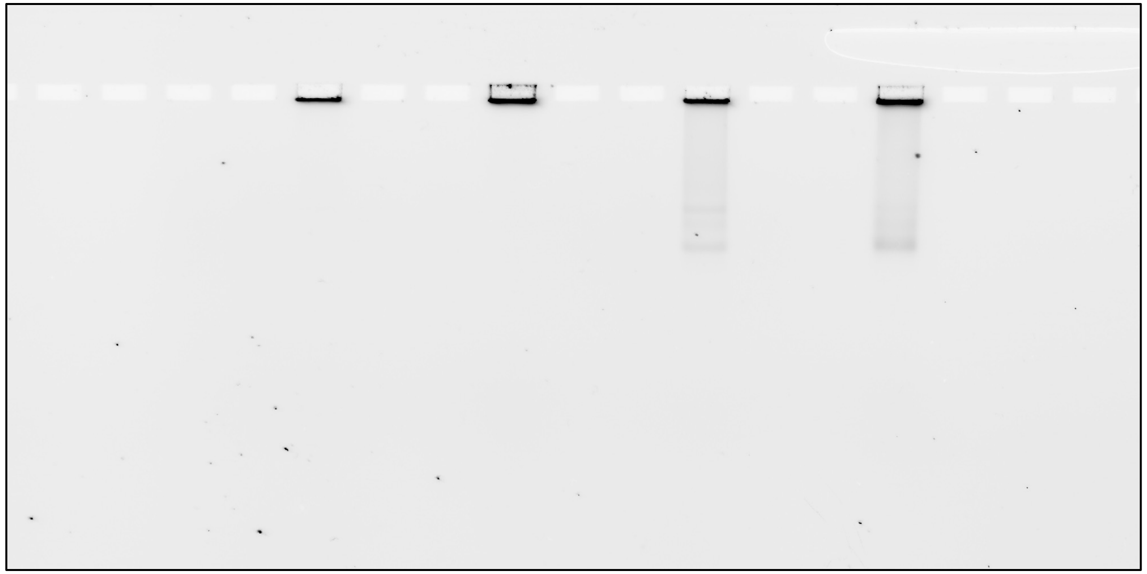

DOPE-Atto488

Source Data

Supplementary Figure 12 a, b

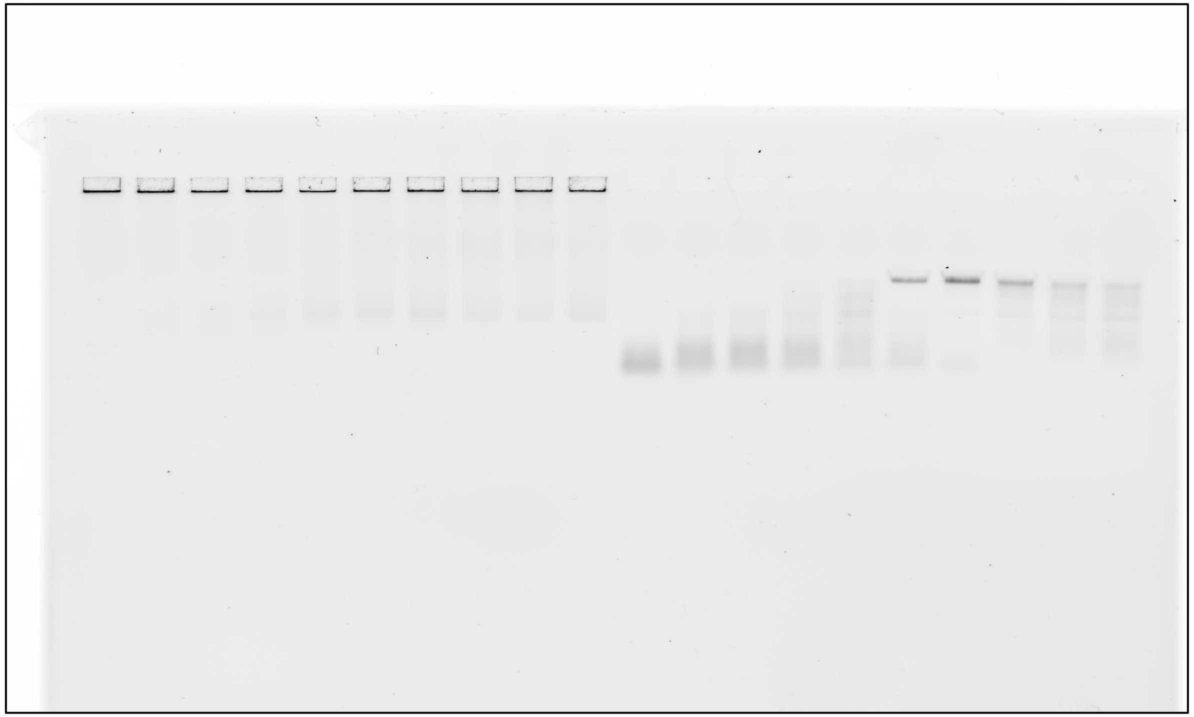

EtBr

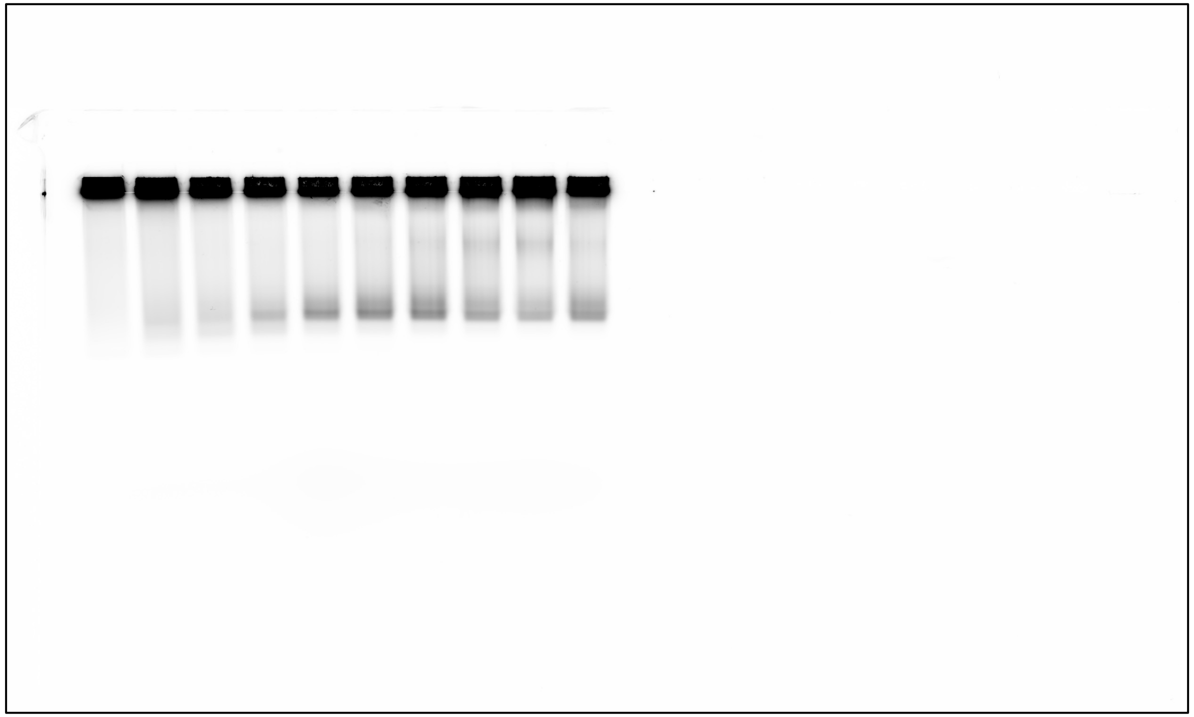

DOPE-Atto643

M. T. Pinner & H. Dietz, *Programmable DNA shell scaffolds for directional membrane budding,* Nat. Commun. 2025

Source Data

Supplementary Figure 13 a, b

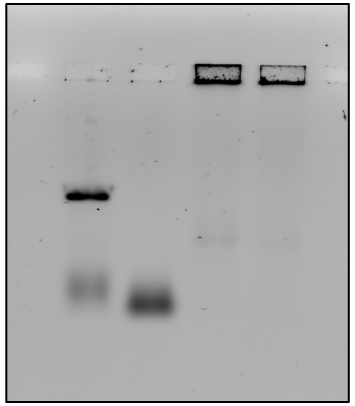

EtBr

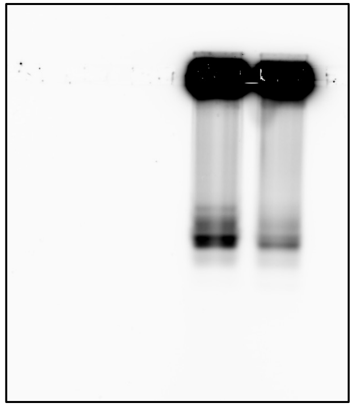

DOPE-Atto643

M. T. Pinner & H. Dietz, *Programmable DNA shell scaffolds for directional membrane budding,* Nat. Commun. 2025

Source Data

Supplementary Figure 14 b (left)

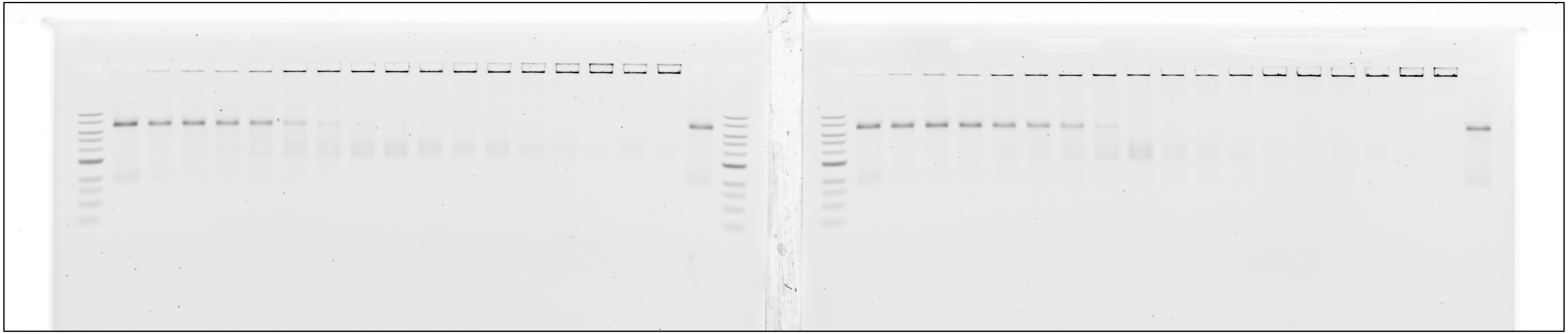

EtBr

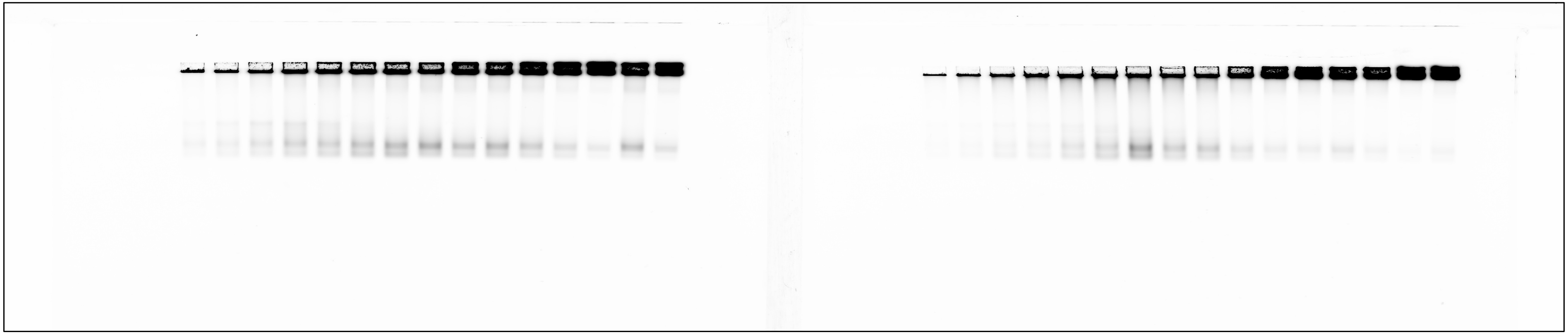

DOPE-Atto643

M. T. Pinner & H. Dietz, *Programmable DNA shell scaffolds for directional membrane budding,* Nat. Commun. 2025

Source Data

Supplementary Figure 14 b (right)

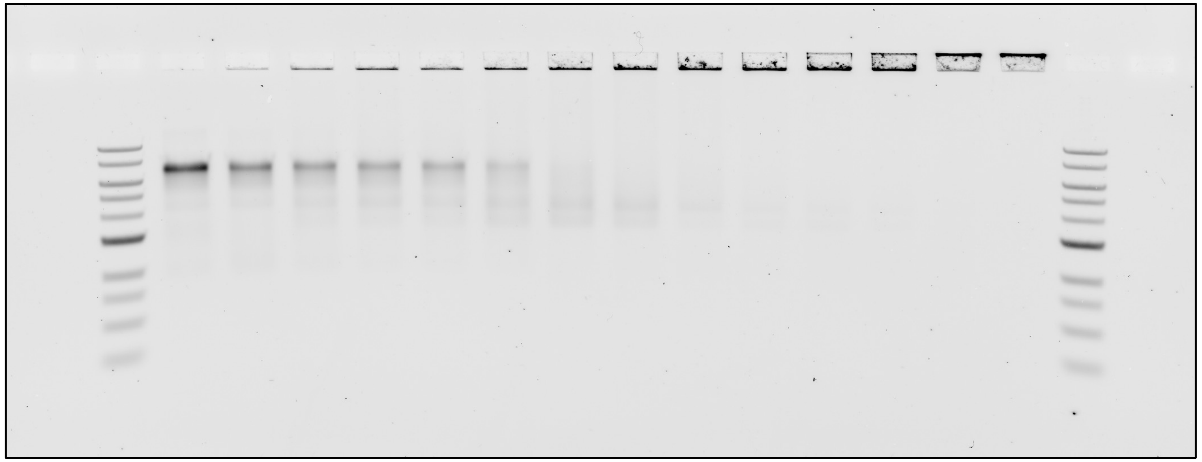

EtBr

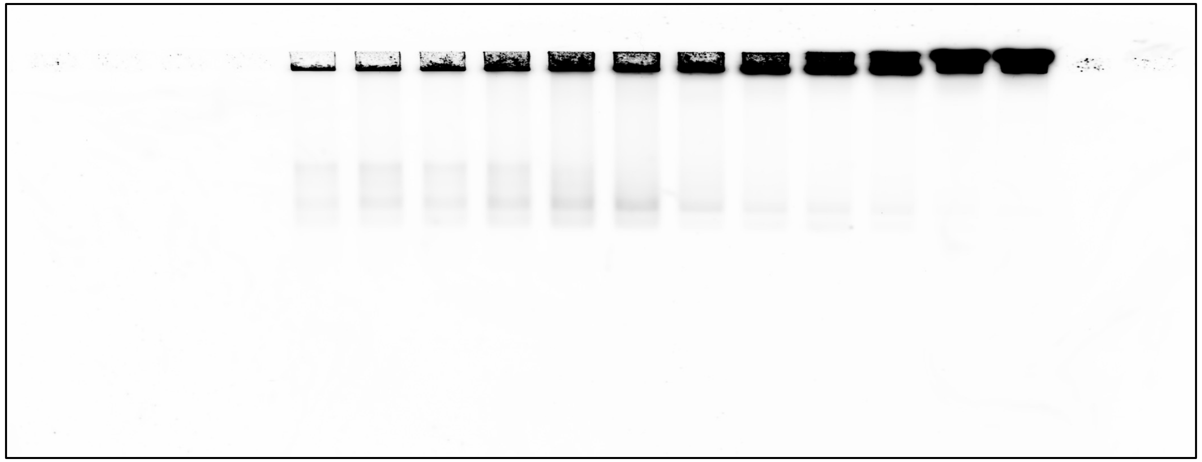

DOPE-Atto643

M. T. Pinner & H. Dietz, *Programmable DNA shell scaffolds for directional membrane budding,* Nat. Commun. 2025

Source Data

Supplementary Figure 15

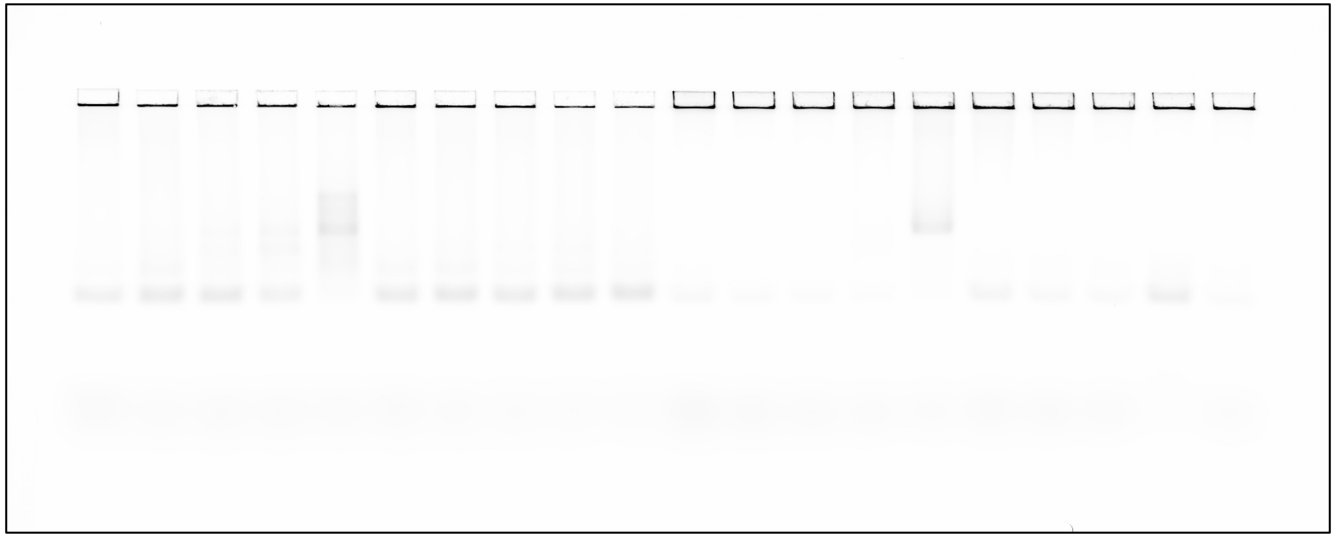

DNA-Atto643

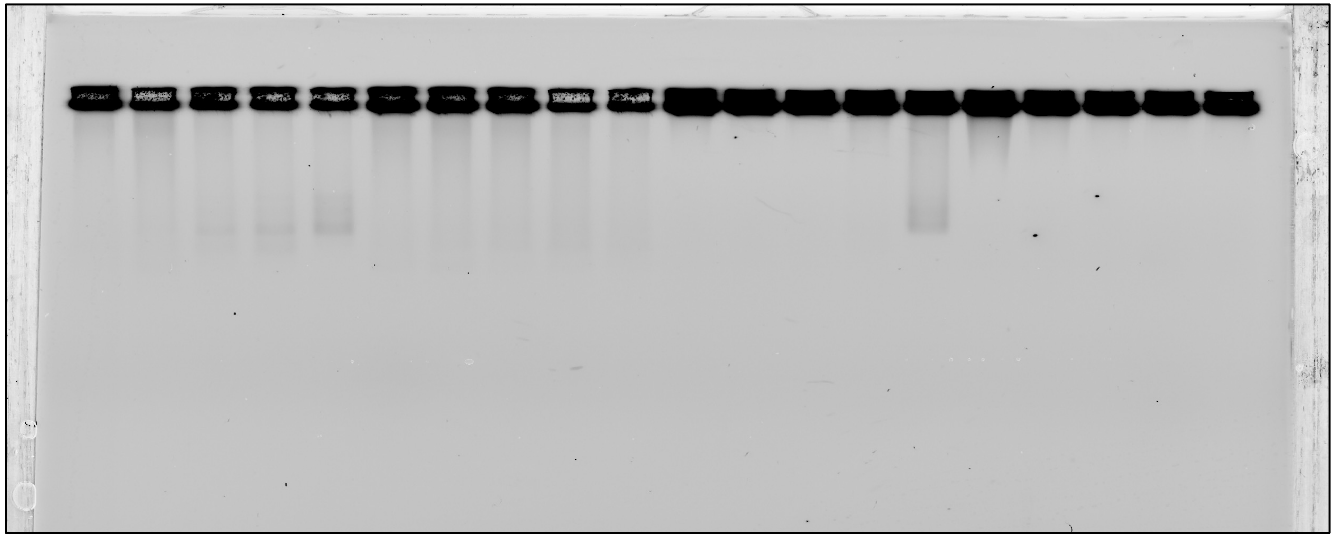

DOPE-Atto488

M. T. Pinner & H. Dietz, *Programmable DNA shell scaffolds for directional membrane budding,* Nat. Commun. 2025

Source Data

Supplementary Figure 16

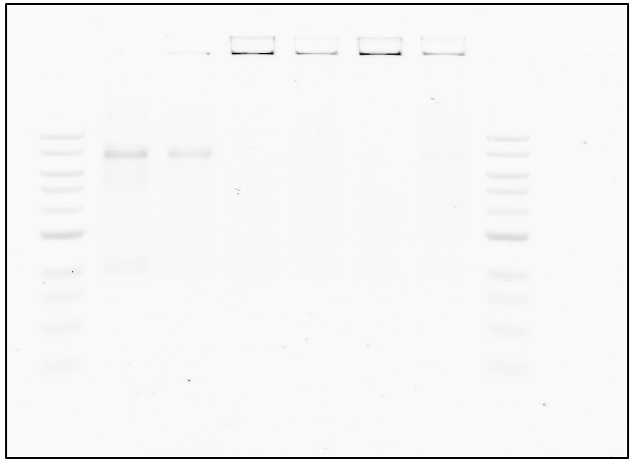

EtBr

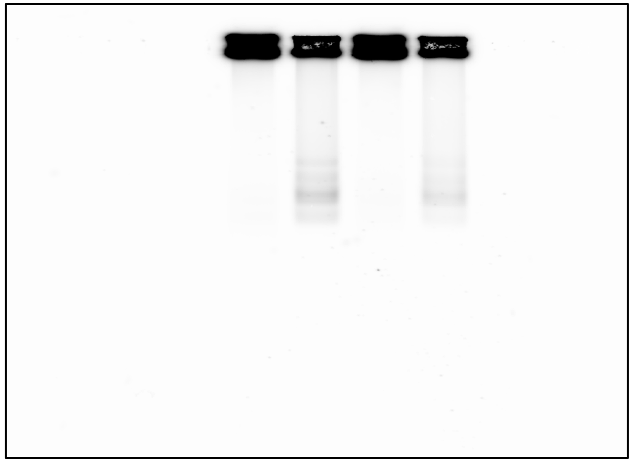

DOPE-Atto643

Source Data

Supplementary Figure 17
(identical to Figure 3a)

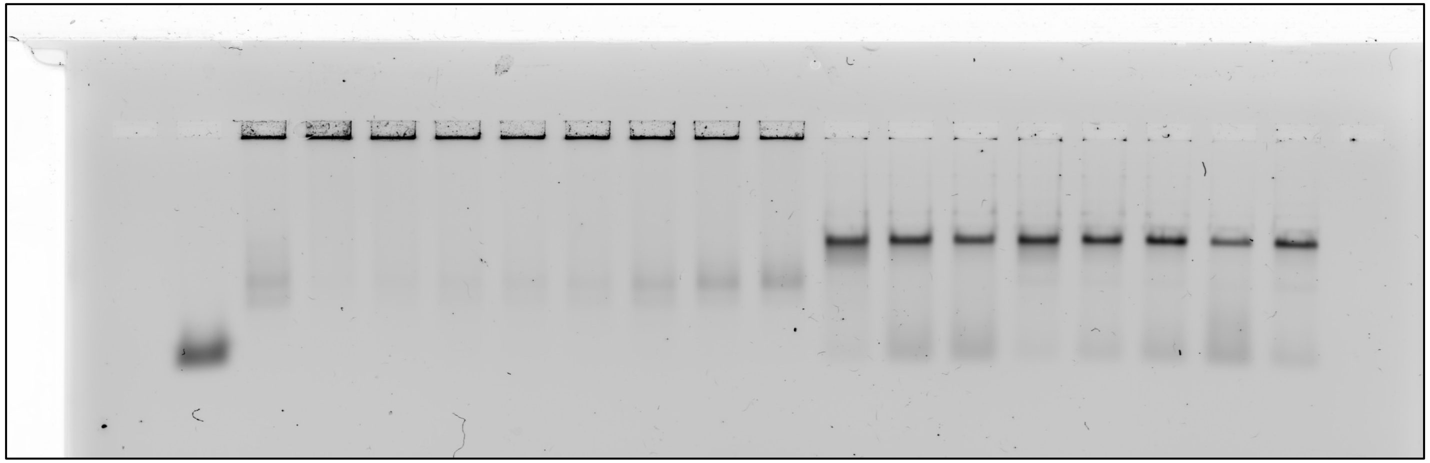

EtBr

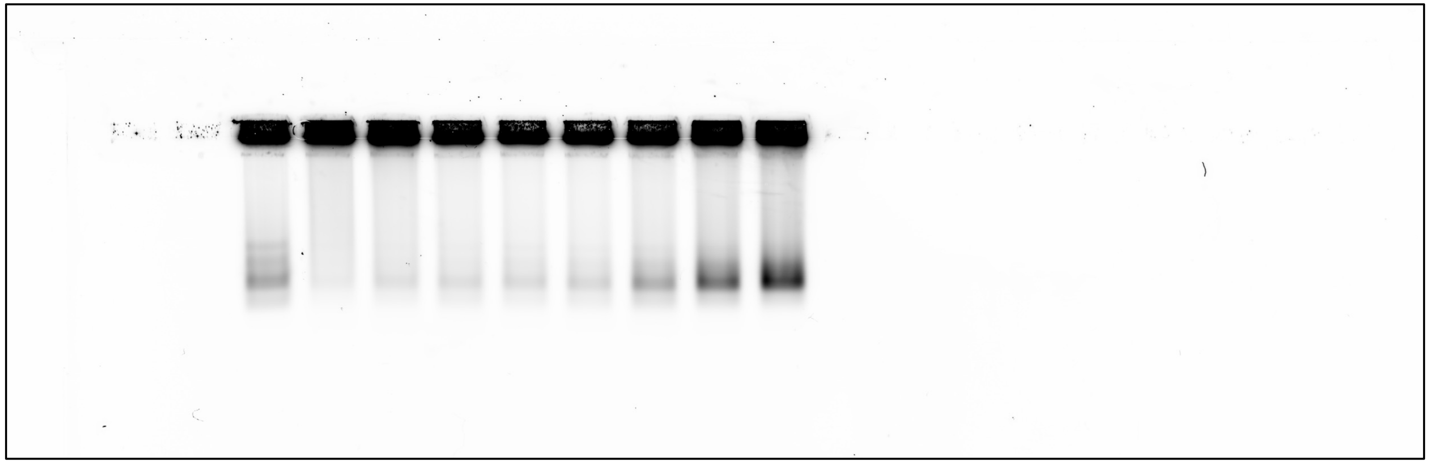

DOPE-Atto643

Source Data
Supplementary Figure 18

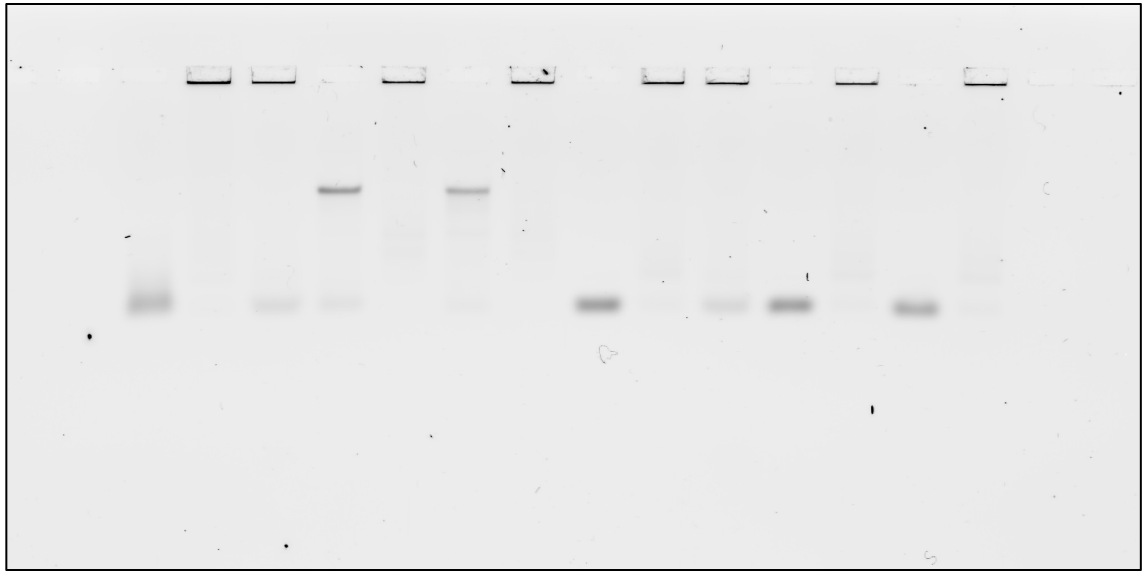

EtBr

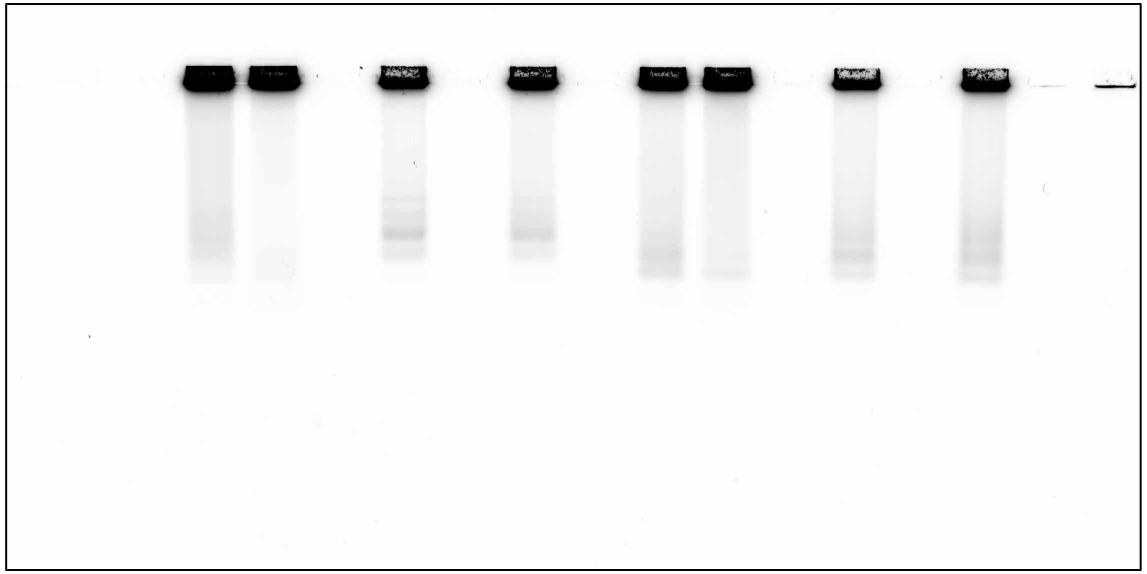

DOPE-Atto643

Source Data

Supplementary Figure 19
(identical to Figure 3b)

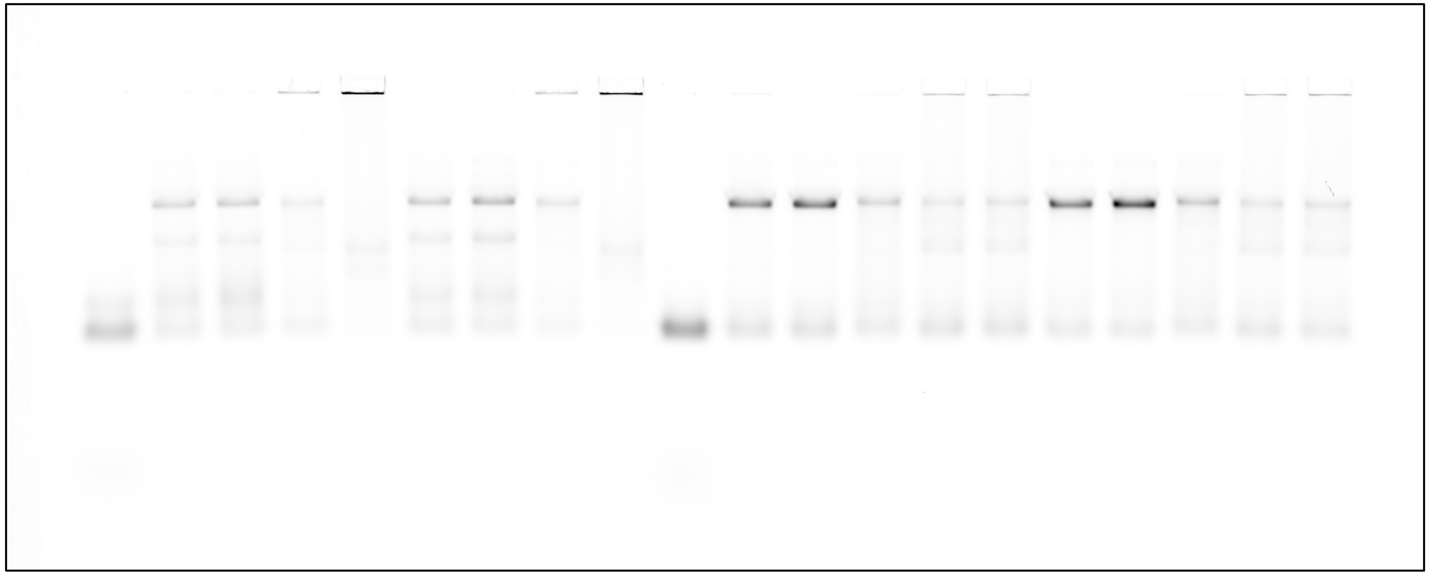

DNA-Atto643

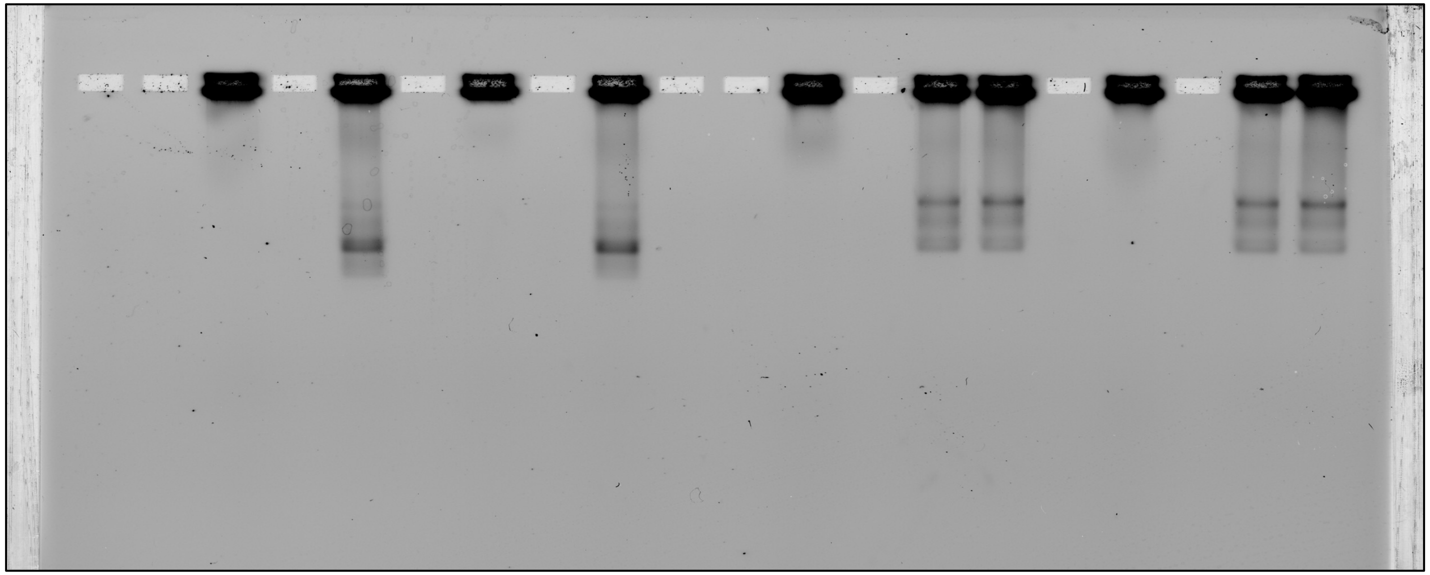

DOPE-Atto488

M. T. Pinner & H. Dietz, *Programmable DNA shell scaffolds for directional membrane budding,* Nat. Commun. 2025

Source Data

Supplementary Figure 20

(identical to Figure 3c)

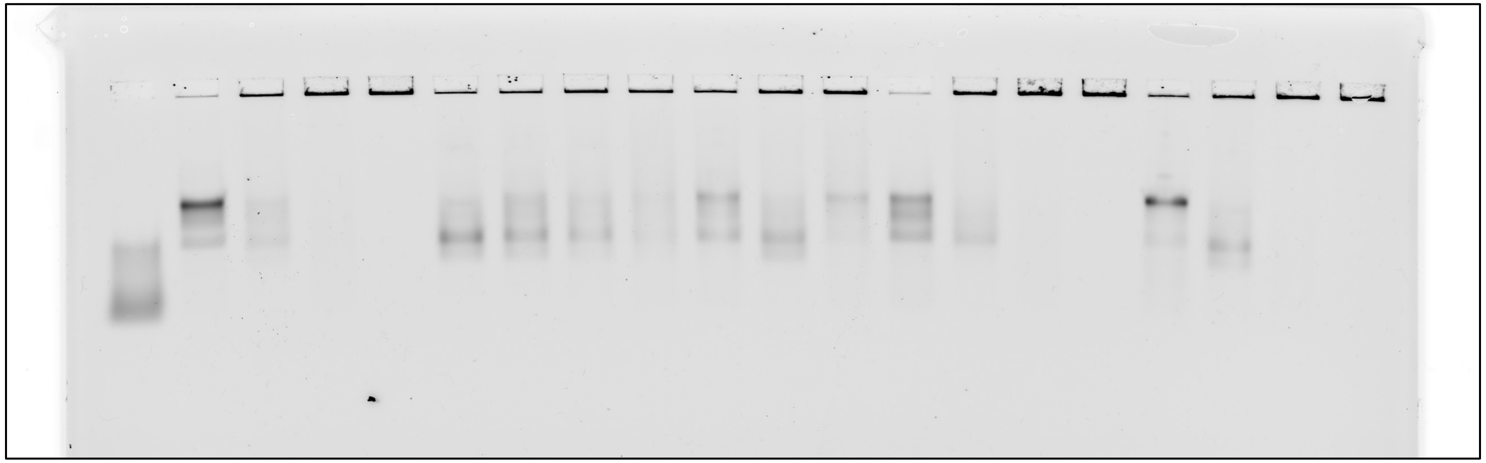

EtBr

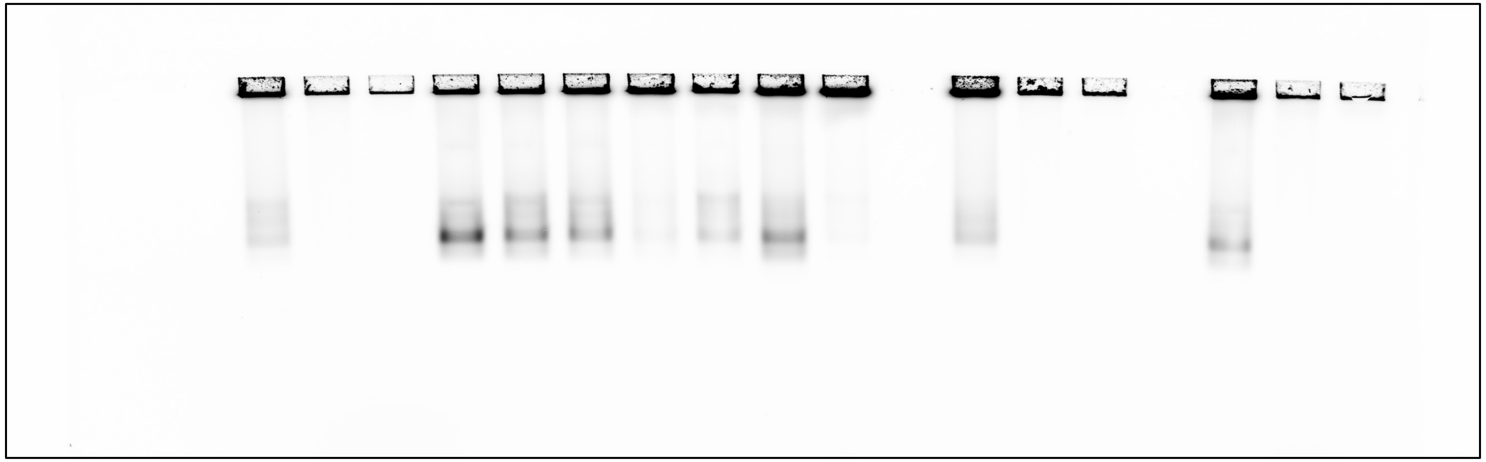

DOPE-Atto643

Source Data

Supplementary Figure 21

(identical to Figure 3d)

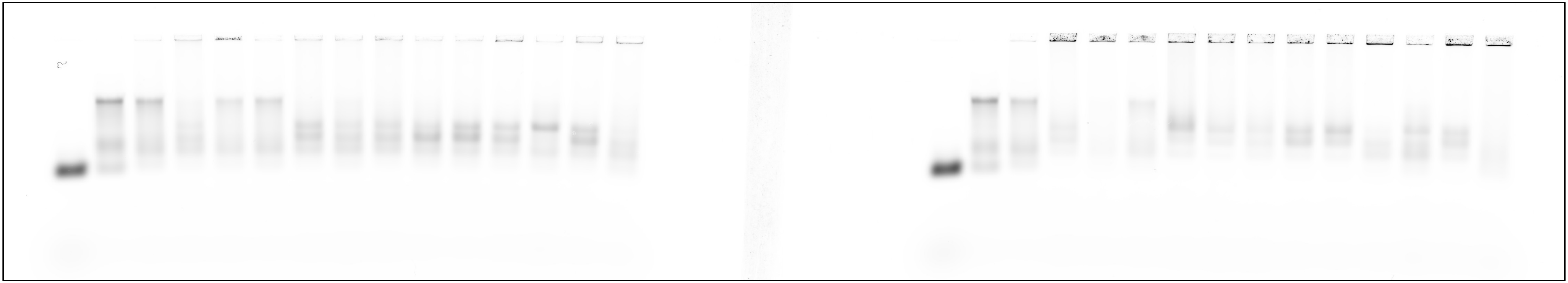

DNA-Atto643

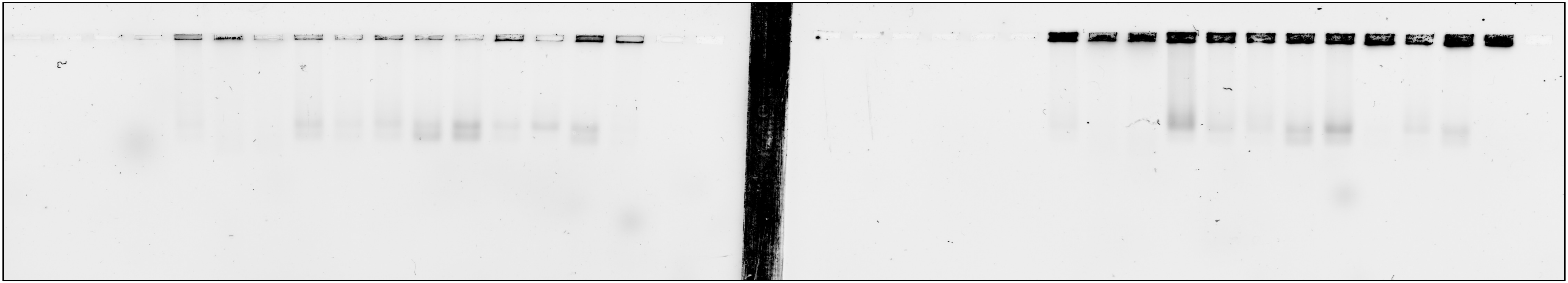

LissRhod-PE

M. T. Pinner & H. Dietz, *Programmable DNA shell scaffolds for directional membrane budding,* Nat. Commun. 2025

Source Data

Supplementary Figure 23

(identical to Figure 3e (1))

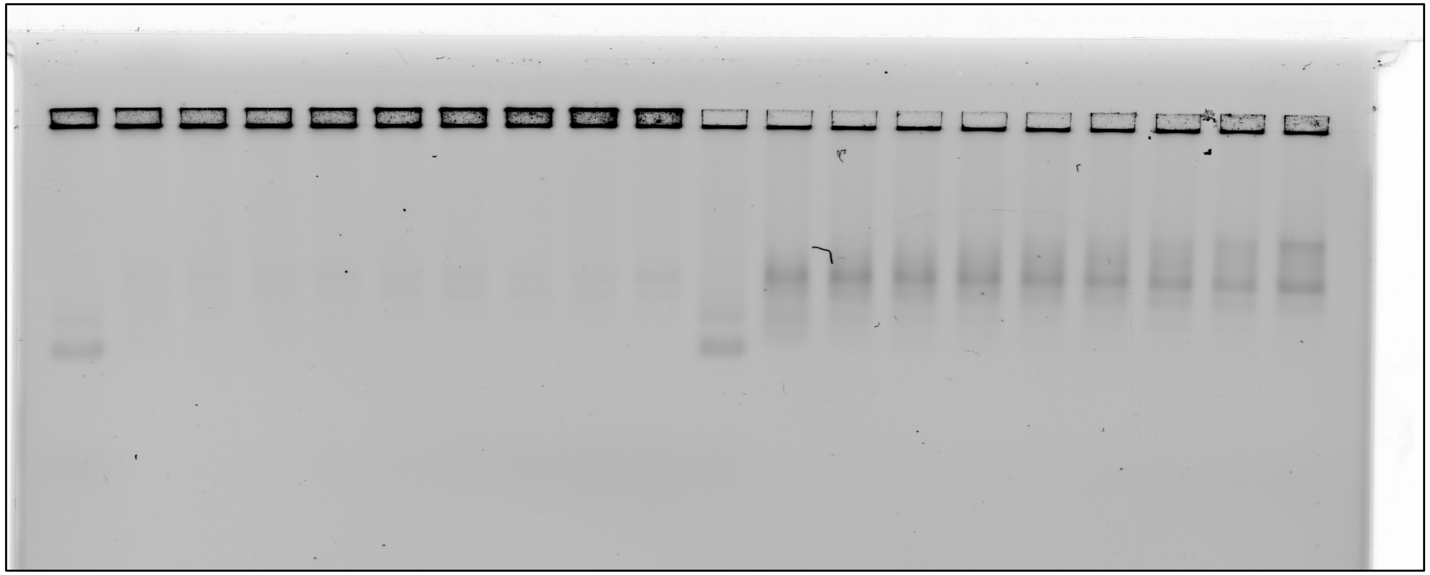

EtBr

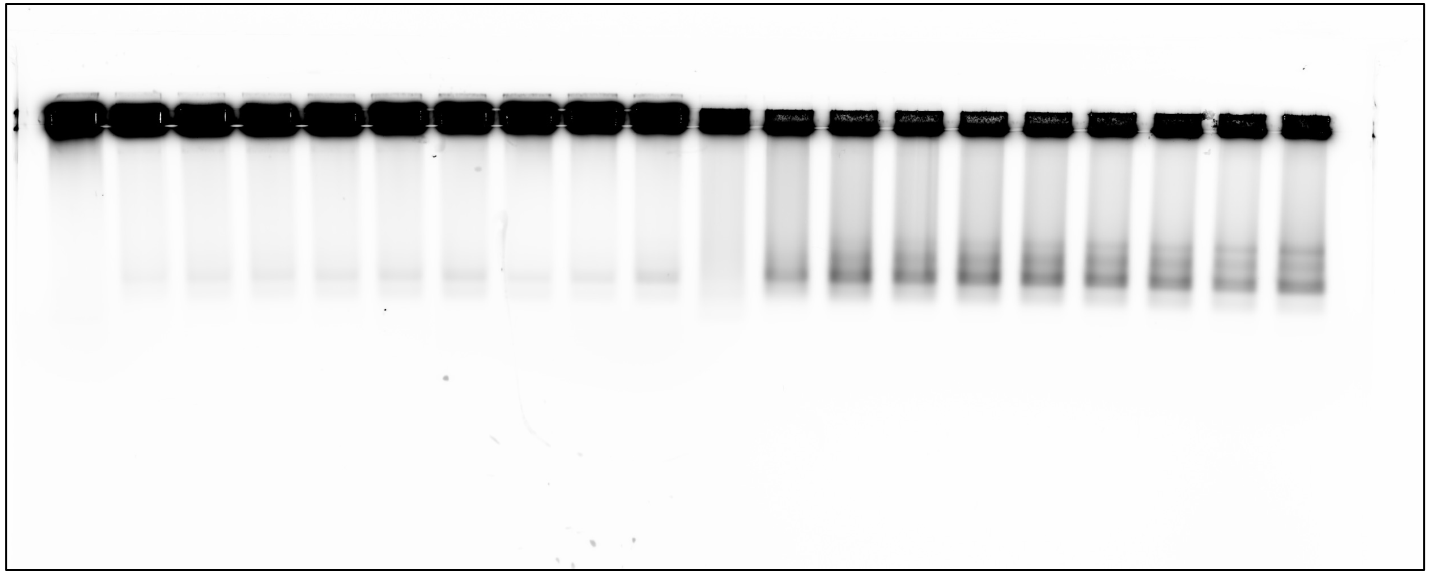

DOPE-Atto643

M. T. Pinner & H. Dietz, *Programmable DNA shell scaffolds for directional membrane budding,* Nat. Commun. 2025
